# Genetically Determined Platelet Count and Risk of Cardiovascular Disease: Mendelian Randomization Study

**DOI:** 10.1161/ATVBAHA.118.311804

**Published:** 2018-10-11

**Authors:** Dipender Gill, Grace Monori, Marios K. Georgakis, Ioanna Tzoulaki, Mike Laffan

**Affiliations:** 1 From the Department of Biostatistics and Epidemiology (D.G., G.M.), School of Public Health, Imperial College London, United Kingdom; 2 MRC-PHE Centre for Environment (I.T.), School of Public Health, Imperial College London, United Kingdom; 3 Institute for Stroke and Dementia Research, University Hospital of Ludwig-Maximilians-University (LMU), Munich, Germany (M.K.G.); 4 Department of Hygiene and Epidemiology, University of Ioannina Medical School, Greece (I.T.); 5 Centre for Haematology, Imperial College London, United Kingdom (M.L.).

**Keywords:** blood platelet, cardiovascular diseases, coronary artery disease, myocardial infarction, stroke

## Abstract

**Objective—:**

Cardiovascular disease, including coronary artery disease (CAD) and ischemic stroke, is the leading cause of death worldwide. This Mendelian randomization study uses genetic variants as instruments to investigate whether there is a causal effect of genetically determined platelet count on CAD and ischemic stroke risk.

**Approach and Results—:**

A genome-wide association study of 166 066 subjects was used to identify instruments and genetic association estimates for platelet count. Genetic association estimates for CAD and ischemic stroke were obtained from genome-wide association studies, including 60 801 CAD cases and 123 504 controls, and 60 341 ischemic stroke cases and 454 450 controls, respectively. The inverse-variance weighted meta-analysis of ratio method Mendelian randomization estimates was the main method used to obtain estimates for the causal effect of genetically determined platelet count on risk of cardiovascular outcomes. We found no significant Mendelian randomization effect of genetically determined platelet count on risk of CAD (odds ratio of CAD per SD unit increase in genetically determined platelet count, 1.01; 95% CI, 0.98–1.04; *P*=0.60). However, higher genetically determined platelet count was causally associated with an increased risk of ischemic stroke (odds ratio, 1.07; 95% CI, 1.04–1.11; *P*<1×10^−5^), including all major ischemic stroke subtypes. Similar results were obtained in sensitivity analyses more robust to the inclusion of pleiotropic genetic variants.

**Conclusions—:**

This Mendelian randomization study found evidence that higher genetically determined platelet count is causally associated with higher risk of ischemic stroke.

## Abstract 2

Supplemental Digital Content is available in the text.

**Highlights**

- Cardiovascular disease is the leading cause of death worldwide.
- This study used Mendelian randomization analysis to test for a causal association between genetically determined platelet count and coronary artery disease, myocardial infarction, ischemic stroke, and ischemic stroke subtype risk.
- Genetically determined platelet count was associated with a higher risk of ischemic stroke and its subtypes.
- This study highlights the importance of platelet count as a risk factor for ischemic stroke and may be used to inform further studies aiming to harness this mechanism for clinical benefit.

Cardiovascular disease (CVD) is the leading cause of death worldwide, with an age-standardized mortality rate of 278 per 100 000 population per year.^1^ The term CVD encompasses a range of conditions, of which coronary artery disease (CAD) and stroke are the most common, with a global prevalence of 154 and 80 million people, respectively,^2^ and together accounting for 85% of CVD deaths.^1^ Therefore, they are a significant public health concern.^1,2^ Platelets are involved in thrombosis, with the 150 000 to 450 000 platelets found per milliliter of blood in healthy individuals forming the basis of blood clots.^3^ Furthermore, platelet aggregation is implicated in CVD risk, with antiplatelet therapy a core component of secondary prevention, as well as primary prevention in high-risk individuals.^4^ Despite the key role of platelets in the pathogenesis of acute CVD events, there is conflicting evidence from observational studies on the association between platelet count and risk of CVD.^5–10^ Greater understanding of this relationship would offer further insight into the pathophysiology of these conditions, which may in turn guide research efforts toward disease prevention.

The Mendelian randomization (MR) approach uses genetic variants associated with an exposure of interest as instruments to investigate the effect of varying that exposure on an outcome (Figure 1).^11,12^ Based on the assumption that the genetic variants used as instruments only associate with the outcome through the exposure, MR is devoid of bias from confounding environmental factors and reverse causation in the way that observational studies can be.^11^ The MR study design is, therefore, comparable to the random allocation of an intervention, or exposure, in a randomized controlled trial.^11^ Two-sample MR uses genetic associations with the exposure and outcome, respectively, from 2 separate population groups on the assumption that these populations are homogeneous,^13^ and can increase the potential scope for the application of the MR methodology.^14^ Genetic variants that may be used as instruments in MR analysis include single-nucleotide polymorphisms (SNPs) that are selected based on their association with the exposure of interest,^15^ which in our present study is platelet count.

**Figure 1.**
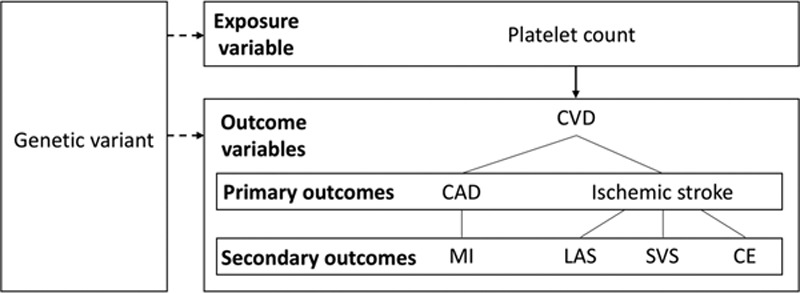
Schematic depiction of our study design, detailing the primary and secondary outcome variables investigated. The dashed arrows represent the associations used in a Mendelian randomization study. The solid arrow represents the Mendelian randomization (MR) causal effect inferred in the results of a Mendelian randomization study. CAD indicates coronary artery disease; CE, cardioembolic; CVD, cardiovascular disease; LAS, large artery stroke; MI, myocardial infarction; and SVS, small vessel stroke.

In this study, we used the MR technique to investigate for a causal effect of genetically determined platelet count on risk of CAD and ischemic stroke. Furthermore, we investigated the causal effect of genetically determined platelet count on risk of myocardial infarction (MI) and ischemic stroke subtypes (large-artery atherosclerotic stroke, small vessel stroke, and cardioembolic stroke) in secondary analyses. The primary and secondary outcomes studied are depicted in Figure 1.

## Materials and Methods

All the data used for this work are available within the article and its online-only Data Supplement.

### SNP-Platelet Count Genetic Association Estimates

Genetic association estimates for platelet count were taken from a genome-wide association study meta-analysis performed using the INTERVAL and UK Biobank studies (including the UK Biobank Lung Exome Variant Evaluation study), which included 166 066 subjects of European ancestry.^16^ Platelet count was measured in a total of 83 565 (45 372 women; 38 193 men) subjects in the UK Biobank study, 43 562 subjects (21 702 women; 21 860 men) in the UK Biobank Lung Exome Variant Evaluation study, and 38 939 (19 661 females; 19 278 males) in the INTERVAL study.^16^ Adjustments were made for age, sex, body mass index, alcohol consumption, and smoking status. The SD of platelet count was 55.3×10^−9^/L (55.3/nL) in women and 51.1×10^−9^/L (51.1/nL) in males for the UK Biobank cohort.^16^ This meta-analysis found 219 SNPs with minor allele frequency >0.01 that were related to platelet count at genome-wide significance (*P* value <5×10^−8^) that had available rsID codes (Table I in the online-only Data Supplement).

The strength of the instruments was assessed using the F statistic, calculated using the equation F=R^2^(n−2)/(1−R^2^), where R^2^ is the proportion of the variability in genetically determined platelet count accounted for by the SNP and n is the sample size.^17^ An F statistic of >10 indicates a relatively low risk of weak instrument bias in MR analyses.^17^

### Association Estimates for SNP-CVD Relationships

The SNP-cardiovascular risk association estimates were obtained from 2 separate genome-wide association study meta-analyses.^18,19^ For CAD and MI, the Coronary Artery Disease Genome-Wide Replication and Meta-Analysis plus Coronary Artery Disease Genetics Consortium’s 1000 genomes-based meta-analysis included data from individuals of European, Hispanic, African American, and South and East Asian ancestry, for 60 801 cases of CAD, of which MI made up ≈70% of cases, and 123 504 control subjects.^18^ Case subjects were defined as those with a documented diagnosis of CAD, such as acute coronary syndrome (including MI), chronic stable angina, or >50% stenosis of at least 1 coronary vessel, as well as those who had undergone percutaneous coronary revascularization or coronary artery bypass grafting.^18^

The MEGASTROKE (Multiancestry Genome-wide Association Study of Stroke) consortium amalgamated data from 29 studies, including data from individuals of European, Latin American, African and East, South and Mixed Asian descent, with 60 341 cases of ischemic stroke, comprising of 9006 cases of cardioembolic stroke, 6688 cases of large-artery atherosclerotic stroke, 11 710 cases of small vessel stroke, and 454 450 control subjects.^19^ Cases of stroke were diagnosed based on the World Health Organization diagnostic criteria of sudden onset of neurological changes lasting at least 24 hours of presumed vascular origin, with subtypes defined using the TOAST criteria.^19^

### Mendelian Randomization Estimates

The association between genetically determined platelet count and CVD risk attributable to each SNP was estimated using the Wald method, which calculates the ratio between the SNP-CVD risk and SNP-platelet count estimates.^14^ The Delta method was used to calculate the SE (second order weights), as this approach acknowledges uncertainty in the genetic association estimates.^14^ The fixed-effect inverse-variance weighted (IVW) meta-analysis method was used to combine MR estimates for the individual SNPs in the main analysis.^14^ The exposure examined was genetically determined platelet count, and the primary outcomes were CAD risk and ischemic stroke risk. Secondary outcomes studied were MI risk, and risk of ischemic stroke subtypes, including large-artery atherosclerotic stroke, cardioembolic stroke, and small vessel stroke. For the 2 primary outcomes, a statistical significance threshold of *P*<0.025 was used after applying a stringent Bonferroni correction for multiple testing. For secondary outcomes, a threshold of *P*<0.05 was used, as these analyses were only performed to further replicate the findings of the primary analyses.

### Investigation of Pleiotropy

Pleiotropy in this context refers to multiple effects of a genetic variant. When SNPs used as instruments in MR analysis are pleiotropic, they may affect the outcome independently of the exposure under consideration.^11^ For example, SNPs used as instruments for platelet count may affect CVD risk through effects independent of platelet count. This can lead to confounding and bias of MR estimates.^11^ Therefore, it is important to investigate for pleiotropy when conducting an MR analysis. To do this, we used several approaches. First, we measured the heterogeneity in MR estimates produced by different SNPs in the fixed-effect IVW meta-analysis using the I^2^ index (I^2^_MR_) and the Cochran Q statistic, based on the principle that if there were no pleiotropic effects occurring then estimates between different SNPs should vary only by chance and, therefore, heterogeneity would be low.^20^ We defined I^2^_MR_>25% and Cochran Q-derived *P* value <0.05 as indicative of excessive heterogeneity and thus pleiotropy.

Furthermore, secondary phenotypes associated with all the instrument SNPs studied were also searched for using PhenoScanner (www.phenoscanner.medschl.cam.ac.uk/phenoscanner).^21^ PhenoScanner is an online database that includes data from publicly available genome-wide association studies with over 10 million SNPs. The secondary SNP associations reaching genome-wide significance (*P* value <5×10^−8^) with linkage disequilibrium r^2^>0.8 were considered as potentially exerting pleiotropy, and sensitivity MR analyses using the fixed-effect IVW method that excluded these SNPs were performed. The details of the 39 SNPs excluded for their potentially pleiotropic effects are shown in Table II in the online-only Data Supplement.

MR-Egger was used as a further statistical sensitivity analysis. This is based on the Egger test used to investigate small-study bias in meta-analyses.^22^ It assumes that any direct effects of the SNPs on the outcome (ie, pleiotropic effects) are independent of their association with platelet count (instrument strength independent of direct effect assumption).^22^ MR-Egger offers a test for pleiotropy that is biasing the MR estimate and also produces MR estimates that are adjusted for this. If the value for the MR-Egger intercept is 0 (tested here using a *P*-value threshold of ≥0.05), then this indicates that there is no evidence of directional pleiotropy being present. Heterogeneity between the individual SNP-platelet count estimates (quantified using an I^2^ index, I^2^_GX_) was used to calculate potential bias in MR-Egger introduced because of measurement error, with an I^2^_GX_ of >95% considered low risk.^23^ We also calculated the Rucker’s Q′ statistic that measures heterogeneity in the MR-Egger analysis, and tested whether it differed significantly from the Cochran’s Q result, as a significantly lower Q′ statistic is indicative of the MR-Egger approach providing a model with better fit for examining the particular association.^24^

In addition, the MR pleiotropy residual sum and outlier (MR-PRESSO) test was used in our sensitivity analyses to test for pleiotropy.^25^ MR-PRESSO detects pleiotropic effects of the SNPs by comparing the residuals for each SNP in the zero-intercept regression line of the SNP-outcome estimates by the SNP-exposure estimates with that expected in the absence of pleiotropic effects.^25^ MR-PRESSO analysis is then again performed after removing the outliers detected, hence correcting for pleiotropic effects.^25^ It is based on the assumption that at least 50% of the SNPs are valid instruments.^25^

The weighted median estimator MR statistical sensitivity test does not rely on the instrument strength is independent of direct effect assumption, and is valid when <50% of the information for the analysis comes from invalid instruments.^26^ Therefore, we also performed weighted median MR analysis, which takes the median estimate from the SNP-platelet count association estimates ranked in order of magnitude and weighted for their precision, with a parametric bootstrap method used to calculate the 95% CIs.^22,26^

Finally, we also performed weighted mode-based estimator MR analysis, which generates an overall MR result from the greatest number of SNPs that produce similar MR estimates, and is reliable when this group of SNPs is valid.^27^ This approach is based on the rationale that the largest number of SNPs producing similar MR estimates may be less likely to be biased because of possible pleiotropic effects.^27^ *P* value thresholds of *P*<0.05 were used in all sensitivity analyses to ascertain statistical significance when replicating the findings of the main analysis.

The data from the meta-analyses used in this study are publicly available, and ethical approval was obtained in the original constituent studies.^16,18,19^ Data were analyzed using the statistical program R (version 3.4.3; The R Foundation for Statistical Computing).

## Results

### SNP-Platelet Count and SNP-CVD Associations

The SNP-platelet count associations for the instrument SNPs identified in the INTERVAL and UK Biobank studies are shown in Table I in the online-only Data Supplement. The instruments had F statistics ranging from 37 to 1526 and were, therefore, at low risk of weak instrument bias (Table I in the online-only Data Supplement).^17^

### Genetically Determined Platelet Count-CVD Risk MR Estimates

#### IVW-MR Analysis

The IVW-MR estimates for individual SNPs are reported in Tables III through VIII in the online-only Data Supplement for CAD, MI, ischemic stroke, cardioembolic stroke, large-artery atherosclerotic stroke, and small vessel stroke, respectively. All MR estimates are reported as odds ratio (OR) of CVD outcome per SD unit increase in genetically determined platelet count. The main IVW-MR analysis did not find higher genetically determined platelet count to be associated with risk of CAD (OR, 1.01; 95% CI, 0.98–1.04; *P*=0.60) or MI (OR, 1.03; 95% CI, 0.99–1.07; *P*=0.12). However, higher genetically determined platelet count was causally associated with a detrimental effect on ischemic stroke risk (OR, 1.07; 95% CI, 1.04–1.11; *P*<1×10^−5^). For the ischemic stroke subtypes, higher genetically determined platelet count was associated with an increased risk of cardioembolic stroke (OR, 1.12; 95% CI, 1.05–1.19; *P*=5×10^−4^), large-artery atherosclerotic stroke (OR, 1.09; 95% CI, 1.01–1.17; *P*=0.03), and small vessel stroke (OR, 1.08; 95% CI, 1.01–1.15 *P*=0.02).

There was evidence of heterogeneity between individual SNP-MR estimates in all analyses performed using the IVW approach (Table X in the online-only Data Supplement), suggestive of potential pleiotropy. Funnel and radial plots to visually evaluate this pleiotropy are provided in Figures I through VI in the online-only Data Supplement. To investigate whether pleiotropic effects were responsible for our results, IVW-MR analysis was conducted after excluding SNPs with potentially pleiotropic effects, as identified by searching the PhenoScanner database^21^ and detailed in Table II in the online-only Data Supplement. After this, we found that there was still no association of genetically determined platelet count with risk of CAD (OR, 1.01; 95% CI, 0.95–1.06; *P*=0.85) or risk of MI (OR, 1.05; 95% CI, 1.00–1.11; *P*=0.07). However, the association between genetically determined platelet count and risk of ischemic stroke remained significant (OR, 1.05; 95% CI, 1.01–1.10; *P*=0.02). Although the MR estimates for risk of cardioembolic stroke (OR, 1.09; 95% CI, 1.00–1.19; *P*=0.05) and large-artery atherosclerotic stroke (OR, 1.06; 95% CI, 0.96–1.18; *P*=0.25) remained similar to the main analysis, they were no longer statistically significant, likely because of wider CIs. The relationship between genetically determined platelet count and increased risk of small vessel stroke remained statistically significant (OR, 1.09; 95% CI, 1.00–1.18; *P*=0.04).

#### MR-Egger

MR-Egger found that a higher genetically determined platelet count was not associated with an increased risk of CAD (OR, 1.11; 95% CI, 0.99–1.24; *P*=0.08) or MI (OR, 1.11; 95% CI, 0.99–1.25; *P*=0.08). As with the main IVW analysis, higher genetically determined platelet count was associated with an increased risk of ischemic stroke (OR, 1.13; 95% CI, 1.03–1.23; *P*=0.01). For the ischemic stroke subtypes, MR-Egger revealed that higher genetically determined platelet count was associated with an increased risk of cardioembolic stroke (OR, 1.19; 95% CI, 1.03–1.39; *P*=0.02), but not large-artery atherosclerotic stroke (OR, 1.18; 95% CI, 0.98–1.43; *P*=0.08), or small vessel stroke (OR, 1.10; 95% CI, 0.93–1.29; *P*=0.26). While the effect estimates remained similar to the main IVW-MR analysis, any loss of statistical significance was attributable to wider CIs. The MR-Egger intercepts, which measure pleiotropy that may bias the main IVW-MR estimates, did not reach statistical significance for any of the CVD subtypes (CAD: *P*=0.05; MI: *P*=0.13; ischemic stroke: *P*=0.21; cardioembolic stroke: *P*=0.36; large-artery atherosclerotic stroke: *P*=0.33; small vessel stroke: *P*=0.84). The I^2^ index (I^2^_GX_) was calculated to test for heterogeneity between the individual SNP-platelet count estimates and was 99%, suggesting therefore there was a low risk of bias with MR-Egger because of measurement error.^23^ Similarly to the IVW analyses, there was evidence of heterogeneity in all MR-Egger analyses, as indicated by the Rucker’s Q′ statistics (Table X in the online-only Data Supplement). However, only for CAD and MI was the Rucker’s Q′ significantly lower than the Cochran’s Q to indicate a better fit for the MR-Egger approach than the IVW method.

#### Mendelian Randomization Pleiotropy Residual Sum and Outlier

Genetically determined platelet count was not associated with an increased risk of CAD in the main MR-PRESSO analysis (OR risk per SD unit increase in genetically determined platelet count: 1.00; 95% CI, 0.95–1.06; *P*=0.92) or outlier-corrected analysis (OR, 1.01; 95% CI, 0.96–1.05; *P*=0.78). Nor was genetically determined platelet count associated with risk of MI in the main MR-PRESSO analysis (OR, 1.03; 95% CI, 0.97–1.09; *P*=0.36) or the outlier-corrected analysis (OR, 1.02; 95% CI, 0.98–1.07; *P*=0.33). In concordance with the main IVW-MR analysis, genetically determined platelet count was associated with an increased risk of ischemic stroke in both the main MR-PRESSO analysis (OR, 1.07; 95% CI, 1.03–1.12; *P*=7×10^-4^) and the outlier-corrected analysis (OR, 1.05; 95% CI, 1.02–1.09; *P*=5×10^−3^). Of the ischemic stroke subtypes, genetically determined platelet count was associated with an increased risk of cardioembolic stroke in the main MR-PRESSO analysis (OR, 1.12; 95% CI, 1.04–1.20; *P*=2×10^−3^). No outliers were detected for cardioembolic stroke. Genetically determined platelet count was not associated with an increased risk of large-artery atherosclerotic stroke in the main MR-PRESSO analysis (OR, 1.09; 95% CI, 1.00–1.19; *P*=0.06) or the outlier-corrected analysis (OR, 1.06; 95% CI, 0.97–1.16; *P*=0.19). Genetically determined platelet count was associated with an increased risk of small vessel stroke in the main MR-PRESSO analysis (OR, 1.08; 95% CI, 1.00–1.17; *P*=0.04) but not the outlier-corrected analysis (OR, 1.06; 95% CI, 0.99–1.14; *P*=0.12). The SNPs excluded in each outlier-corrected MR-PRESSO analysis are presented in Table IX in the online-only Data Supplement. The distortion *P* value was 0.92 for CAD, 0.88 for MI, 0.12 for ischemic stroke, 0.32 for large-artery atherosclerotic stroke, and 0.39 for small vessel stroke, suggesting that any outlier SNPs were unlikely to be significantly biasing the estimates.

#### Weighted Median MR

Weighted median MR analyses showed that genetically determined platelet count was not associated with risk of CAD (OR, 1.04; 95% CI, 0.97–1.10; *P*=0.27), or MI (OR, 1.02; 95% CI, 0.95–1.09; *P*=0.54). However, higher genetically determined platelet count was associated with an increased risk of ischemic stroke (OR, 1.08; 95% CI, 1.03–1.14; *P*=3×10^−3^). For the ischemic stroke subtypes, higher genetically determined platelet count was associated with an increased risk of cardioembolic stroke (OR, 1.16; 95% CI, 1.03–1.30; *P*=0.01), but not risk of large-artery atherosclerotic stroke (OR, 1.07; 95% CI, 0.94–1.23; *P*=0.30), or small vessel stroke (OR, 1.09; 95% CI, 0.98–1.21; *P*=0.13). As with the other sensitivity analyses, although the estimate remained similar, the size of the CIs increased.

### Weighted Mode-Based Estimator MR

Finally, the weighted-mode based MR also produced similar MR estimates to the main analysis, but with wider 95% CIs. There was no evidence of an MR effect of genetically determined platelet count on risk of CAD (OR, 0.99; 95% CI, 0.91–1.08; *P*=0.85) or MI (OR, 1.01; 95% CI, 0.91–1.11; *P*=0.91). However, for ischemic stroke (OR, 1.07; 95% CI, 1.00–1.15; *P*=0.06) and its subtypes (cardioembolic stroke: OR, 1.18; 95% CI, 0.99–1.40; *P*=0.07; large-artery atherosclerotic stroke: OR, 1.04; 95% CI, 0.87–1.25; *P*=0.65; and small vessel stroke: OR, 1.14; 95% CI, 0.98–1.33; *P*=0.09), there was a suggestion of increased risk with higher genetically determined platelet count, but this did not reach statistical significance.

**Figure 2.**
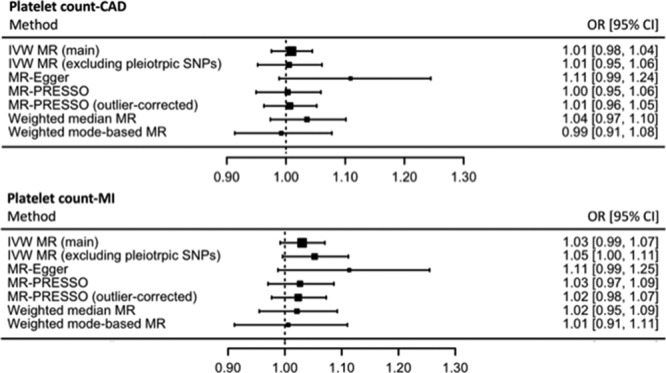
Summary Mendelian randomization (MR) estimates derived from the main inverse-variance weighted (IVW), IVW excluding potentially pleiotropic single-nucleotide polymorphisms (SNPs), MR-Egger, MR pleiotropy residual sum and outlier (PRESSO), outlier-corrected MR-PRESSO, weighted median and weighted mode-based estimator methods for coronary artery disease (CAD), and myocardial infarction (MI). OR indicates odds ratio.

**Figure 3.**
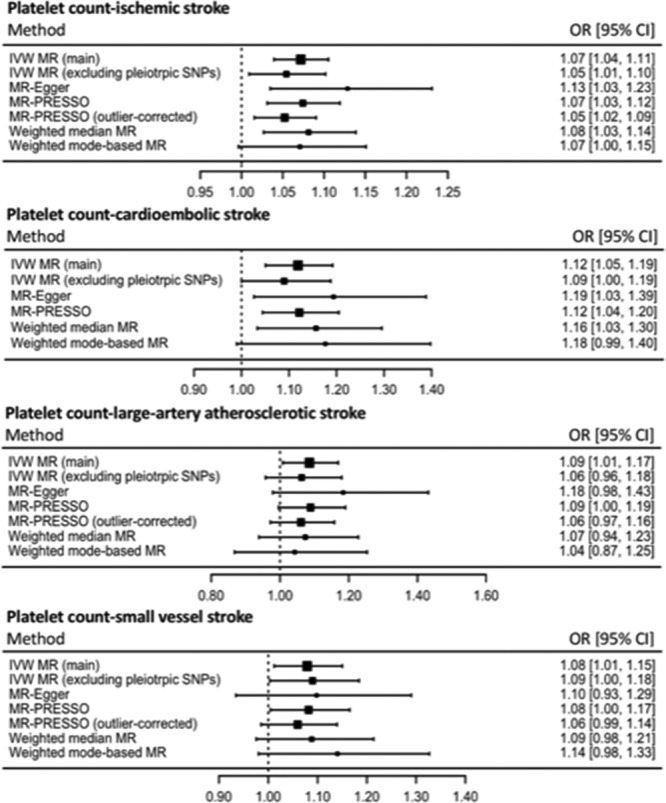
Summary MR estimates derived from the main inverse-variance weighted (IVW), IVW excluding potentially pleiotropic single-nucleotide polymorphisms (SNPs), Mendelian randomization (MR)-Egger, MR pleiotropy residual sum and outlier (PRESSO), outlier-corrected MR-PRESSO, weighted median and weighted mode-based estimator methods for ischemic stroke, cardioembolic stroke, large-artery atherosclerotic stroke, and small vessel stroke. OR indicates odds ratio.

Summary results of the MR estimates on CVD risk derived from the main IVW, IVW excluding potentially pleiotropic SNPs, MR-Egger, MR-PRESSO, outlier-corrected MR-PRESSO, weighted median, and weighted mode-based estimator methods are presented in Figure 2 for CAD and MI, and Figure 3 for ischemic stroke, cardioembolic stroke, large-artery atherosclerotic stroke, and small vessel stroke.

## Discussion

This study investigated the effect of genetically determined platelet count on risk of CAD and ischemic stroke using an MR approach. We report evidence that a higher genetically determined platelet count was associated with increased risk of ischemic stroke, but not CAD nor MI. Both MI and ischemic stroke typically result from occlusion to blood flow by a thrombus,^28,29^ and therefore platelets have a critical role in the pathogenesis of both diseases.^28^ Given this commonality, it is somewhat unexpected that we find that evidence for higher genetically determined platelet count being associated with an increased risk of ischemic stroke, but not CAD or MI. Studies investigating the composition of thrombi in acute MI found that platelets accounted for 17%,^30^ while this was between 30% and 50% for ischemic stroke.^31^ The greater relative contribution of platelets to the thrombi in ischemic stroke compared with MI may go some way to explaining the findings of our study, with a higher platelet count having more effect on diseases where platelets make up a larger proportion of the thrombus. Additionally, some of the discrepancy in the findings between CAD and ischemic stroke may relate to the caliber of the respective vessels, with coronary arteries generally having a larger diameter than cerebral arteries,^32,33^ with potential implications on the pathophysiology of thrombus formation. Furthermore, it may be that our investigation for the association between platelet count and risk of CAD or MI-lacked sufficient statistical power. Indeed, for the analyses of MI, there was some suggestion of increased risk with higher platelet count in all MR analyses (Figure 2), although this did not reach statistical significance. Furthermore, the CAD-MR analyses may have been affected by pleiotropic SNPs, with the MR-Egger test for this just missing statistical significance (*P*=0.05), and the MR-Egger estimate that adjusted for this approaching statistical significance (OR, 1.11; 95% CI, 0.99–1.24; *P*=0.08). Comparisons of clot structure between stroke subtypes suggest that cardioembolic strokes have greater proportions of platelets^34^ and fibrin^35^ than large-artery strokes, consistent with the findings reported here. It is therefore surprising that antiplatelet therapy is of limited benefit for stroke prevention in the context of atrial fibrillation, although the benefit from anticoagulation may be explained by the high fibrin content.

### Findings in Context

The effect of platelet traits on CAD risk has previously been investigated using an MR framework, and in keeping with our current findings, no effect of genetically determined platelet count was identified.^16^ Our study goes further to also explore ischemic stroke as an outcome, for which we do find MR evidence of an effect of genetically determined platelet count on overall risk of ischemic stroke, as well as its subtypes.

While some observational work has found an association between a higher platelet count and risk of CAD,^36^ and a higher platelet count in cases of MI or unstable angina compared with controls,^8^ others studies have reported no significant difference in platelet count between cases and controls for CAD or MI.^37–39^ Furthermore, a cohort study of hemodialysis patients found that higher platelet count was associated with a lower risk of CAD,^39^ and although it is unclear whether this is generalizable to healthy individuals, other studies have also reported a higher platelet count to be associated with lower risk of MI.^9,40^ Of note, case-control studies may be affected by the measurement of platelet count after the incident case of CAD, which may limit interpretation because of possible reverse causation. For example, Yaghoubi et al^40^ measured platelet count up to 24 hours after symptom onset in MI or unstable angina, while Panwar et al^8^ measured platelet count up to 24 hours after admission with an MI or unstable angina event.

Looking at ischemic stroke, a case-control study based in Turkey found that platelet count was significantly higher in cases compared with controls.^7^ These results were replicated in case-control studies in Hungary^41^ and in China.^6^ However, another case-control study set in China reported no significant difference between platelet count in cases and controls.^5^ Furthermore, the Cardiovascular Health Study, including a cohort of 5766 individuals, of whom 807 developed ischemic strokes, found no relationship between platelet count and risk of ischemic stroke after 12 to 15 years of follow-up.^9^ These results were supported by the European Prospective Investigation into Cancer and Nutrition-Netherlands cohort study (average follow-up 11.4 years), which also reported no relationship between platelet count and risk of stroke.^36^

Other cohort studies have looked at overall CVD risk, and a cohort study in Denmark with a 3.5 year follow-up period found that platelet count was associated with increased risk of CVD.^10^ In contrast to the case-control studies, the cohort studies carry the benefit of measuring platelet count before ischemic stroke incidence, avoiding spurious associations from reverse causation. A summary of the key findings of the discussed observational studies in this area is available in Table XI in the online-only Data Supplement.

### Strengths and Limitations

A consistent limitation of all of the discussed observational studies is that platelet count is affected by a range of conditions, which may confound the analyses. For example, thrombocytopenia is associated with malignancy, liver disease, autoimmune diseases, pregnancy, and infections.^42^ Thrombocythemia is associated with iron deficiency, hematologic malignancy, and chronic inflammation.^43^ Thus, the key advantage of the MR approach used in our study is that it overcomes this potential source of bias by using genetic variants as instruments for platelet count.^13,16,18^ Furthermore, MR uses genetic variants that are allocated at conception as instruments for the exposure of interest and is therefore not affected by reverse causation as case-control studies might be.

We have taken a rigorous methodological approach in our MR analyses. The SNPs selected as instruments for the MR analysis were associated with platelet count at genome-wide significance, reducing the risk of using invalid instruments. Furthermore, the F statistics were above 10 for all SNPs, indicating that the instruments were unlikely to suffer marked weak instrument bias.^17^ We incorporated a range of sensitivity analyses to explore potential bias because of genetic pleiotropy in our approach. For these, the effect estimates were generally similar to those from the main IVW-MR analyses, although wider CIs were observed reflecting the reduced statistical power associated with these approaches (Figures 2 and 3). Of particular note, platelet count is closely related to other platelet and more general hematologic traits, both at an observational and a genetic level,^16^ and it was therefore important that we made every effort to investigate whether our MR estimates were biased by pleiotropic variants. While approaches such as multivariable MR allow adjustments to be made for any genetic association of the instruments with traits through which pleiotropy is likely to be exerted,^44^ this approach was not appropriate for our current work. Specifically, multivariable MR can be severely biased in the case of overlapping traits, such as would be expected for platelet and hematologic characteristics.^45^

MR assumes a linear relationship between the exposure and outcome,^11^ in our case genetically determined platelet count and disease risk. For this reason, the results of our MR analysis should not be extrapolated to extremes of platelet count. Use of genome-wide association studies may also result in the Winner’s curse,^15^ which can result in overestimation of the SNP-exposure estimate taken from the same population used to identify instruments, and bias of our consequent 2-sample MR estimate toward the null. Of further note, by using genetic variants as a marker of platelet count, MR accounts for the lifetime effect of this exposure.^11^ The MR results for the association between genetically determined platelet count and CVD risk should, therefore, not be directly extrapolated to estimate the effect of any potential clinical intervention targeting platelet count.

## Conclusions

This study used MR to investigate the association between genetically determined platelet count and cardiovascular risk. We report an increased risk of ischemic stroke with higher genetically determined platelet count. Stroke is the second largest cause of death worldwide,^1^ therefore, understanding its associated risk factors is a clinical and public health priority. These findings may thus guide further work toward identifying preventative strategies.

## Acknowledgments

D. Gill designed the study. D. Gill, G. Monori, and M. Georgakis performed statistical analysis, interpreted the results and drafted the article. D. Gill, G. Monori, M. Georgakis, I. Tzoulaki, and M. Laffan critically revised the article for intellectual content and approved the submitted version of the article. D. Gill and G. Monori are accountable for the accuracy and integrity of the work. The authors offer their thanks and appreciation to the Coronary Artery Disease Genome-wide Replication And Meta-analysis plus Coronary Artery Disease genetics (CARDIoGRAMplusC4D) Consortium,^18^ the International Human Epigenome Consortium,^16^ UK Biobank, INTERVAL, and UK BiLEVE, and the MEGASTROKE (Multiancestry Genome-wide Association Study of Stroke) project^19^ for making the data used in this study publicly available. The MEGASTROKE project was funded by sources detailed at http://www.megastroke.org/acknowledgements.html. The full list of MEGASTROKE authors is available in the supplemental methods available online.

## Sources of Funding

D. Gill led this study as part of a Wellcome 4i Clinical PhD Programme at Imperial College London.

## Disclosures

None.

## Supplementary Material
