# Supplementary material for: Genetically Determined Platelet Count and Risk of Cardiovascular Disease: Mendelian Randomization Study
Source: Arterioscler Thromb Vasc Biol. 2018 Oct 11;38(12):2862–9. doi: 10.1161/ATVBAHA.118.311804 (PMC6250250; doi:10.1161/ATVBAHA.118.311804)
Supplement: Supplementary file 1 [file atv-38-2862-s001.pdf]

Supplemental Material

Supplemental Methods

Full author list of MEGASTROKE Consortium[1]

Rainer Malik [1], Ganesh Chauhan [2], Matthew Traylor [3], Muralidharan Sargurupremraj [4,5], Yukinori Okada [6,7,8], Aniket Mishra [4,5], Loes Rutten-Jacobs [3], Anne-Katrin Giese [9], Sander W van der Laan [10], Solveig Gretarsdottir [11], Christopher D Anderson [12,13,14,14], Michael Chong [15], Hieab HH Adams [16,17], Tetsuro Ago [18], Peter Almgren [19], Philippe Amouyel [20,21], Hakan Ay [22,13], Traci M Bartz [23], Oscar R Benavente [24], Steve Bevan [25], Giorgio B Boncoraglio [26], Robert D Brown, Jr. [27], Adam S Butterworth [28,29], Caty Carrera [30,31], Cara L Carty [32,33], Daniel I Chasman [34,35], Wei-Min Chen [36], John W Cole [37], Adolfo Correa [38], Ioana Cotlarciuc [39], Carlos Cruchaga [40,41], John Danesh [28,42,43,44], Paul IW de Bakker [45,46], Anita L DeStefano [47,48], Marcel den Hoed [49], Qing Duan [50], Stefan T Engelter [51,52], Guido J Falcone [53,54], Rebecca F Gottesman [55], Raji P Grewal [56], Vilmundur Gudnason [57,58], Stefan Gustafsson [59], Jeffrey Haessler [60], Tamara B Harris [61], Ahamad Hassan [62], Aki S Havulinna [63,64], Susan R Heckbert [65], Elizabeth G Holliday [66,67], George Howard [68], Fang-Chi Hsu [69], Hyacinth I Hyacinth [70], M Arfan Ikram [16], Erik Ingelsson [71,72], Marguerite R Irvin [73], Xueqiu Jian [74], Jordi Jiménez-Conde [75], Julie A Johnson [76,77], J Wouter Jukema [78], Masahiro Kanai [6,7,79], Keith L Keene [80,81], Brett M Kissela [82], Dawn O Kleindorfer [82], Charles Kooperberg [60], Michiaki Kubo [83], Leslie A Lange [84], Carl D Langefeld [85], Claudia Langenberg [86], Lenore J Launer [87], Jin-Moo Lee [88], Robin Lemmens [89,90], Didier Leys [91], Cathryn M Lewis [92,93], Wei-Yu Lin [28,94], Arne G Lindgren [95,96], Erik Lorentzen [97], Patrik K Magnusson [98], Jane Maguire [99], Ani Manichaikul [36], Patrick F McArdle [100], James F Meschia [101], Braxton D Mitchell [100,102], Thomas H Mosley [103,104], Michael A Nalls [105,106], Toshiharu Ninomiya [107], Martin J O'Donnell [15,108], Bruce M Psaty [109,110,111,112], Sara L Pulit [113,45], Kristiina Rannikmäe [114,115], Alexander P Reiner [65,116], Kathryn M Rexrode [117], Kenneth Rice [118], Stephen S Rich [36], Paul M Ridker [34,35], Natalia S Rost [9,13], Peter M Rothwell [119], Jerome I Rotter [120,121], Tatjana Rundek [122], Ralph L Sacco [122], Saori Sakaue [7,123], Michele M Sale [124], Veikko Salomaa [63], Bishwa R Sapkota [125], Reinhold Schmidt [126], Carsten O Schmidt [127], Ulf Schminke [128], Pankaj Sharma [39], Agnieszka Slowik [129], Cathie LM Sudlow [114,115], Christian Tanislav [130], Turgut Tatlisumak [131,132], Kent D Taylor [120,121], Vincent NS Thijs [133,134], Gudmar Thorleifsson [11], Unnur Thorsteinsdottir [11], Steffen Tiedt [1], Stella Trompet [135], Christophe Tzourio [5,136,137], Cornelia M van Duijn [138,139], Matthew Walters [140], Nicholas J Wareham [86], Sylvia Wassertheil-Smoller [141], James G Wilson [142], Kerri L Wiggins [109], Qiong Yang [47], Salim Yusuf [15], Najaf Amin [16], Hugo S Aparicio [185,48], Donna K Arnett [186], John Attia [187], Alexa S Beiser [47,48], Claudine Berr [188], Julie E Buring [34,35], Mariana Bustamante [189], Valeria Caso [190], Yu-Ching Cheng [191], Seung Hoan Choi [192,48], Ayesha Chowhan [185,48], Natalia Cullell [31], Jean-François Dartigues [193,194], Hossein Delavaran [95,96], Pilar Delgado [195], Marcus Dörr [196,197], Gunnar Engström [19], Ian Ford [198], Wander S Gurpreet [199], Anders Hamsten [200,201], Laura Heitsch [202], Atsushi Hozawa [203], Laura Ibanez [204], Andreea Ilinca [95,96], Martin Ingelsson [205], Motoki Iwasaki [206], Rebecca D Jackson [207],

Katarina Jood [208], Pekka Jousilahti [63], Sara Kaffashian [4,5], Lalit Kalra [209], Masahiro Kamouchi [210], Takanari Kitazono [211], Olafur Kjartansson [212], Manja Kloss [213], Peter J Koudstaal [214], Jerzy Krupinski [215], Daniel L Labovitz [216], Cathy C Laurie [118], Christopher R Levi [217], Linxin Li [218], Lars Lind [219], Cecilia M Lindgren [220,221], Vasileios Lioutas [222,48], Yong Mei Liu [223], Oscar L Lopez [224], Hirata Makoto [225], Nicolas Martinez-Majander [172], Koichi Matsuda [225], Naoko Minegishi [203], Joan Montaner [226], Andrew P Morris [227,228], Elena Muiño [31], Martina Müller-Nurasyid [229,230,231], Bo Norrving [95,96], Soichi Ogishima [203], Eugenio A Parati [232], Leema Reddy Peddareddygari [56], Nancy L Pedersen [98,233], Joanna Pera [129], Markus Perola [63,234], Alessandro Pezzini [235], Silvana Pileggi [236], Raquel Rabionet [237], Iolanda Riba-Llena [30], Marta Ribasés [238], Jose R Romero [185,48], Jaume Roquer [239,240], Anthony G Rudd [241,242], Antti-Pekka Sarin [243,244], Ralhan Sarju [199], Chloe Sarnowski [47,48], Makoto Sasaki [245], Claudia L Satizabal [185,48], Mamoru Satoh [245], Naveed Sattar [246], Norie Sawada [206], Gerli Sibolt [172], Ásgeir Sigurdsson [247], Albert Smith [248], Kenji Sobue [245], Carolina Soriano-Tárraga [240], Tara Stanne [249], O Colin Stine [250], David J Stott [251], Konstantin Strauch [229,252], Takako Takai [203], Hideo Tanaka [253,254], Kozo Tanno [245], Alexander Teumer [255], Liisa Tomppo [172], Nuria P Torres-Aguila [31], Emmanuel Touze [256,257], Shoichiro Tsugane [206], Andre G Uitterlinden [258], Einar M Valdimarsson [259], Sven J van der Lee [16], Henry Völzke [255], Kenji Wakai [253], David Weir [260], Stephen R Williams [261], Charles DA Wolfe [241,242], Quenna Wong [118], Huichun Xu [191], Taiki Yamaji [206], Dharambir K Sanghera [125,169,170], Olle Melander [19], Christina Jern [171], Daniel Strbian [172,173], Israel Fernandez-Cadenas [31,30], W T Longstreth, Jr [174,65], Arndt Rolfs [175], Jun Hata [107], Daniel Woo [82], Jonathan Rosand [12,13,14], Guillaume Pare [15], Jemma C Hopewell [176], Danish Saleheen [177], Kari Stefansson [11,178], Bradford B Worrall [179], Steven J Kittner [37], Sudha Seshadri [180,48], Myriam Fornage [74,181], Hugh S Markus [3], Joanna MM Howson [28], Yoichiro Kamatani [6,182], Stephanie Debette [4,5], Martin Dichgans [1,183,184]

1 Institute for Stroke and Dementia Research (ISD), University Hospital, LMU Munich, Munich, Germany
2 Centre for Brain Research, Indian Institute of Science, Bangalore, India
3 Stroke Research Group, Division of Clinical Neurosciences, University of Cambridge, UK
4 INSERM U1219 Bordeaux Population Health Research Center, Bordeaux, France
5 University of Bordeaux, Bordeaux, France
6 Laboratory for Statistical Analysis, RIKEN Center for Integrative Medical Sciences, Yokohama, Japan
7 Department of Statistical Genetics, Osaka University Graduate School of Medicine, Osaka, Japan
8 Laboratory of Statistical Immunology, Immunology Frontier Research Center (WPI-IFReC), Osaka University, Suita, Japan.
9 Department of Neurology, Massachusetts General Hospital, Harvard Medical School, Boston, MA, USA
10 Laboratory of Experimental Cardiology, Division of Heart and Lungs, University Medical Center Utrecht, University of Utrecht, Utrecht,Netherlands
11 deCODE genetics/AMGEN inc, Reykjavik, Iceland

12 Center for Genomic Medicine, Massachusetts General Hospital (MGH), Boston, MA, USA
13 J. Philip Kistler Stroke Research Center, Department of Neurology, MGH, Boston, MA, USA
14 Program in Medical and Population Genetics, Broad Institute, Cambridge, MA, USA
15 Population Health Research Institute, McMaster University, Hamilton, Canada
16 Department of Epidemiology, Erasmus University Medical Center, Rotterdam, Netherlands
17 Department of Radiology and Nuclear Medicine, Erasmus University Medical Center, Rotterdam, Netherlands
18 Department of Medicine and Clinical Science, Graduate School of Medical Sciences, Kyushu University, Fukuoka, Japan
19 Department of Clinical Sciences, Lund University, Malmö, Sweden
20 Univ. Lille, Inserm, Institut Pasteur de Lille, LabEx DISTALZ-UMR1167, Risk factors and molecular determinants of aging-related diseases, F-59000 Lille, France
21 Centre Hosp. Univ Lille, Epidemiology and Public Health Department, F-59000 Lille, France
22 AA Martinos Center for Biomedical Imaging, Department of Radiology, Massachusetts General Hospital, Harvard Medical School, Boston, MA, USA
23 Cardiovascular Health Research Unit, Departments of Biostatistics and Medicine, University of Washington, Seattle, WA, USA
24 Division of Neurology, Faculty of Medicine, Brain Research Center, University of British Columbia, Vancouver, Canada
25 School of Life Science, University of Lincoln, Lincoln, UK
26 Department of Cerebrovascular Diseases, Fondazione IRCCS Istituto Neurologico "Carlo Besta", Milano, Italy
27 Department of Neurology, Mayo Clinic Rochester, Rochester, MN, USA
28 MRC/BHF Cardiovascular Epidemiology Unit, Department of Public Health and Primary Care, University of Cambridge, Cambridge, UK
29 The National Institute for Health Research Blood and Transplant Research Unit in Donor Health and Genomics, University of Cambridge, UK
30 Neurovascular Research Laboratory, Vall d'Hebron Institut of Research, Neurology and Medicine Departments-Universitat Autònoma de Barcelona, Vall d’Hebrón Hospital, Barcelona, Spain
31 Stroke Pharmacogenomics and Genetics, Fundacio Docència i Recerca MutuaTerrassa, Terrassa, Spain
32 Children's Research Institute, Children's National Medical Center, Washington, DC, USA
33 Center for Translational Science, George Washington University, Washington, DC, USA
34 Division of Preventive Medicine, Brigham and Women's Hospital, Boston, MA, USA
35 Harvard Medical School, Boston, MA, USA
36 Center for Public Health Genomics, Department of Public Health Sciences, University of Virginia, Charlottesville, VA, USA

37 Department of Neurology, University of Maryland School of Medicine and Baltimore VAMC, Baltimore, MD, USA
38 Departments of Medicine, Pediatrics and Population Health Science, University of Mississippi Medical Center, Jackson, MS, USA
39 Institute of Cardiovascular Research, Royal Holloway University of London, UK & Ashford and St Peters Hospital, Surrey UK
40 Department of Psychiatry,The Hope Center Program on Protein Aggregation and Neurodegeneration (HPAN),Washington University, School of Medicine, St. Louis, MO, USA
41 Department of Developmental Biology, Washington University School of Medicine, St. Louis, MO, USA
42 NIHR Blood and Transplant Research Unit in Donor Health and Genomics, Department of Public Health and Primary Care, University of Cambridge, Cambridge, UK
43 Wellcome Trust Sanger Institute, Wellcome Trust Genome Campus, Hinxton, Cambridge, UK
44 British Heart Foundation, Cambridge Centre of Excellence, Department of Medicine, University of Cambridge, Cambridge, UK
45 Department of Medical Genetics, University Medical Center Utrecht, Utrecht, Netherlands
46 Department of Epidemiology, Julius Center for Health Sciences and Primary Care, University Medical Center Utrecht, Utrecht, Netherlands
47 Boston University School of Public Health, Boston, MA, USA
48 Framingham Heart Study, Framingham, MA, USA
49 Department of Immunology, Genetics and Pathology and Science for Life Laboratory, Uppsala University, Uppsala, Sweden
50 Department of Genetics, University of North Carolina, Chapel Hill, NC, USA
51 Department of Neurology and Stroke Center, Basel University Hospital, Switzerland
52 Neurorehabilitation Unit, University and University Center for Medicine of Aging and Rehabilitation Basel, Felix Platter Hospital, Basel, Switzerland
53 Department of Neurology, Yale University School of Medicine, New Haven, CT, USA
54 Program in Medical and Population Genetics, The Broad Institute of Harvard and MIT, Cambridge, MA, USA
55 Department of Neurology, Johns Hopkins University School of Medicine, Baltimore, MD, USA
56 Neuroscience Institute, SF Medical Center, Trenton, NJ, USA
57 Icelandic Heart Association Research Institute, Kopavogur, Iceland
58 University of Iceland, Faculty of Medicine, Reykjavik, Iceland
59 Department of Medical Sciences, Molecular Epidemiology and Science for Life Laboratory, Uppsala University, Uppsala, Sweden
60 Division of Public Health Sciences, Fred Hutchinson Cancer Research Center, Seattle, WA, USA
61 Laboratory of Epidemiology and Population Science, National Institute on Aging, National Institutes of Health, Bethesda, MD, USA

62 Department of Neurology, Leeds General Infirmary, Leeds Teaching Hospitals NHS Trust, Leeds, UK
63 National Institute for Health and Welfare, Helsinki, Finland
64 FIMM - Institute for Molecular Medicine Finland, Helsinki, Finland
65 Department of Epidemiology, University of Washington, Seattle, WA, USA
66 Public Health Stream, Hunter Medical Research Institute, New Lambton, Australia
67 Faculty of Health and Medicine, University of Newcastle, Newcastle, Australia
68 School of Public Health, University of Alabama at Birmingham, Birmingham, AL, USA
69 Department of Biostatistical Sciences, Wake Forest School of Medicine, Winston-Salem, NC, USA
70 Aflac Cancer and Blood Disorder Center, Department of Pediatrics, Emory University School of Medicine, Atlanta, GA, USA
71 Department of Medicine, Division of Cardiovascular Medicine, Stanford University School of Medicine, CA, USA
72 Department of Medical Sciences, Molecular Epidemiology and Science for Life Laboratory, Uppsala University, Uppsala, Sweden
73 Epidemiology, School of Public Health, University of Alabama at Birmingham, USA
74 Brown Foundation Institute of Molecular Medicine, University of Texas Health Science Center at Houston, Houston, TX, USA
75 Neurovascular Research Group (NEUVAS), Neurology Department, Institut Hospital del Mar d'Investigació Mèdica, Universitat Autònoma de Barcelona, Barcelona, Spain
76 Department of Pharmacotherapy and Translational Research and Center for Pharmacogenomics, University of Florida, College of Pharmacy, Gainesville, FL, USA
77 Division of Cardiovascular Medicine, College of Medicine, University of Florida, Gainesville, FL, USA
78 Department of Cardiology, Leiden University Medical Center, Leiden, the Netherlands
79 Program in Bioinformatics and Integrative Genomics, Harvard Medical School, Boston, MA, USA
80 Department of Biology, East Carolina University, Greenville, NC, USA
81 Center for Health Disparities, East Carolina University, Greenville, NC, USA
82 University of Cincinnati College of Medicine, Cincinnati, OH, USA
83 RIKEN Center for Integrative Medical Sciences, Yokohama, Japan
84 Department of Medicine, University of Colorado Denver, Anschutz Medical Campus, Aurora, CO, USA
85 Center for Public Health Genomics and Department of Biostatistical Sciences, Wake Forest School of Medicine, Winston-Salem, NC, USA
86 MRC Epidemiology Unit, University of Cambridge School of Clinical Medicine, Institute of Metabolic Science, Cambridge Biomedical Campus, Cambridge, UK
87 Intramural Research Program, National Institute on Aging, National Institutes of Health, Bethesda, MD, USA

88 Department of Neurology, Radiology, and Biomedical Engineering, Washington University School of Medicine, St. Louis, MO, USA
89 KU Leuven – University of Leuven, Department of Neurosciences, Experimental Neurology, Leuven, Belgium
90 VIB Center for Brain & Disease Research, University Hospitals Leuven, Department of Neurology, Leuven, Belgium
91 Univ.-Lille, INSERM U 1171. CHU Lille. Lille, France
92 Department of Medical and Molecular Genetics, King's College London, London, UK
93 SGDP Centre, Institute of Psychiatry, Psychology & Neuroscience, King's College London, London, UK
94 Northern Institute for Cancer Research, Paul O'Gorman Building, Newcastle University, Newcastle, UK
95 Department of Clinical Sciences Lund, Neurology, Lund University, Lund, Sweden
96 Department of Neurology and Rehabilitation Medicine, Skåne University Hospital, Lund, Sweden
97 Bioinformatics Core Facility, University of Gothenburg, Gothenburg, Sweden
98 Department of Medical Epidemiology and Biostatistics, Karolinska Institutet, Stockholm, Sweden
99 University of Technology Sydney, Faculty of Health, Ultimo, Australia
100 Department of Medicine, University of Maryland School of Medicine, MD, USA
101 Department of Neurology, Mayo Clinic, Jacksonville, FL, USA
102 Geriatrics Research and Education Clinical Center, Baltimore Veterans Administration Medical Center, Baltimore, MD, USA
103 Division of Geriatrics, School of Medicine, University of Mississippi Medical Center, Jackson, MS, USA
104 Memory Impairment and Neurodegenerative Dementia Center, University of Mississippi Medical Center, Jackson, MS, USA
105 Laboratory of Neurogenetics, National Institute on Aging, National institutes of Health, Bethesda, MD, USA
106 Data Tecnica International, Glen Echo MD, USA
107 Department of Epidemiology and Public Health, Graduate School of Medical Sciences, Kyushu University, Fukuoka, Japan
108 Clinical Research Facility, Department of Medicine, NUI Galway, Galway, Ireland
109 Cardiovascular Health Research Unit, Department of Medicine, University of Washington, Seattle, WA, USA
110 Department of Epidemiology, University of Washington, Seattle, WA
111 Department of Health Services, University of Washington, Seattle, WA, USA
112 Kaiser Permanente Washington Health Research Institute, Seattle, WA, USA
113 Brain Center Rudolf Magnus, Department of Neurology, University Medical Center Utrecht, Utrecht, The Netherlands
114 Usher Institute of Population Health Sciences and Informatics, University of Edinburgh, Edinburgh, UK
115 Centre for Clinical Brain Sciences, University of Edinburgh, Edinburgh, UK

116 Fred Hutchinson Cancer Research Center, University of Washington, Seattle, WA, USA
117 Department of Medicine, Brigham and Women's Hospital, Boston, MA, USA
118 Department of Biostatistics, University of Washington, Seattle, WA, USA
119 Nuffield Department of Clinical Neurosciences, University of Oxford, UK
120 Institute for Translational Genomics and Population Sciences, Los Angeles Biomedical Research Institute at Harbor-UCLA Medical Center, Torrance, CA, USA
121 Division of Genomic Outcomes, Department of Pediatrics, Harbor-UCLA Medical Center, Torrance, CA, USA
122 Department of Neurology, Miller School of Medicine, University of Miami, Miami, FL, USA
123 Department of Allergy and Rheumatology, Graduate School of Medicine, the University of Tokyo, Tokyo, Japan
124 Center for Public Health Genomics, University of Virginia, Charlottesville, VA, USA
125 Department of Pediatrics, College of Medicine, University of Oklahoma Health Sciences Center, Oklahoma City, OK, USA
126 Department of Neurology, Medical University of Graz, Graz, Austria
127 University Medicine Greifswald, Institute for Community Medicine, SHIP-KEF, Greifswald, Germany
128 University Medicine Greifswald, Department of Neurology, Greifswald, Germany
129 Department of Neurology, Jagiellonian University, Krakow, Poland
130 Department of Neurology, Justus Liebig University, Giessen, Germany
131 Department of Clinical Neurosciences/Neurology, Institute of Neuroscience and Physiology, Sahlgrenska Academy at University of Gothenburg, Gothenburg, Sweden
132 Sahlgrenska University Hospital, Gothenburg, Sweden
133 Stroke Division, Florey Institute of Neuroscience and Mental Health, University of Melbourne, Heidelberg, Australia
134 Austin Health, Department of Neurology, Heidelberg, Australia
135 Department of Internal Medicine, Section Gerontology and Geriatrics, Leiden University Medical Center, Leiden, the Netherlands
136 INSERM U1219, Bordeaux, France
137 Department of Public Health, Bordeaux University Hospital, Bordeaux, France
138 Genetic Epidemiology Unit, Department of Epidemiology, Erasmus University Medical Center Rotterdam, Netherlands
139 Center for Medical Systems Biology, Leiden, Netherlands
140 School of Medicine, Dentistry and Nursing at the University of Glasgow, Glasgow, UK
141 Department of Epidemiology and Population Health, Albert Einstein College of Medicine, NY, USA
142 Department of Physiology and Biophysics, University of Mississippi Medical Center, Jackson, MS, USA
143 A full list of members and affiliations appears in the Supplementary Note
144 Department of Human Genetics, McGill University, Montreal, Canada

145 Department of Pathophysiology, Institute of Biomedicine and Translation Medicine, University of Tartu, Tartu, Estonia
146 Department of Cardiac Surgery, Tartu University Hospital, Tartu, Estonia
147 Clinical Gene Networks AB,Stockholm, Sweden
148 Department of Genetics and Genomic Sciences, The Icahn Institute for Genomics and Multiscale Biology Icahn School of Medicine at Mount Sinai, New York, NY , USA
149 Department of Pathophysiology, Institute of Biomedicine and Translation Medicine, University of Tartu, Biomeedikum, Tartu, Estonia
150 Integrated Cardio Metabolic Centre, Department of Medicine, Karolinska Institutet, Karolinska Universitetssjukhuset, Huddinge, Sweden.
151 Clinical Gene Networks AB, Stockholm, Sweden
152 Sorbonne Universités, UPMC Univ. Paris 06, INSERM, UMR_S 1166, Team Genomics & Pathophysiology of Cardiovascular Diseases, Paris, France
153 ICAN Institute for Cardiometabolism and Nutrition, Paris, France
154 Department of Biomedical Engineering, University of Virginia, Charlottesville, VA, USA
155 Group Health Research Institute, Group Health Cooperative, Seattle, WA, USA
156 Seattle Epidemiologic Research and Information Center, VA Office of Research and Development, Seattle, WA, USA
157 Cardiovascular Research Center, Massachusetts General Hospital, Boston, MA, USA
158 Department of Medical Research, Bærum Hospital, Vestre Viken Hospital Trust, Gjettum, Norway
159 Saw Swee Hock School of Public Health, National University of Singapore and National University Health System, Singapore
160 National Heart and Lung Institute, Imperial College London, London, UK
161 Department of Gene Diagnostics and Therapeutics, Research Institute, National Center for Global Health and Medicine, Tokyo, Japan
162 Department of Epidemiology, Tulane University School of Public Health and Tropical Medicine, New Orleans, LA, USA
163 Department of Cardiology,University Medical Center Groningen, University of Groningen, Netherlands
164 MRC-PHE Centre for Environment and Health, School of Public Health, Department of Epidemiology and Biostatistics, Imperial College London, London, UK
165 Department of Epidemiology and Biostatistics, Imperial College London, London, UK
166 Department of Cardiology, Ealing Hospital NHS Trust, Southall, UK
167 National Heart, Lung and Blood Research Institute, Division of Intramural Research, Population Sciences Branch, Framingham, MA, USA
168 A full list of members and affiliations appears at the end of the manuscript
169 Department of Phamaceutical Sciences, Collge of Pharmacy, University of Oklahoma Health Sciences Center, Oklahoma City, OK, USA
170 Oklahoma Center for Neuroscience, Oklahoma City, OK, USA
171 Department of Pathology and Genetics, Institute of Biomedicine, The Sahlgrenska Academy at University of Gothenburg, Gothenburg, Sweden

172 Department of Neurology, Helsinki University Hospital, Helsinki, Finland
173 Clinical Neurosciences, Neurology, University of Helsinki, Helsinki, Finland
174 Department of Neurology, University of Washington, Seattle, WA, USA
175 Albrecht Kossel Institute, University Clinic of Rostock, Rostock, Germany
176 Clinical Trial Service Unit and Epidemiological Studies Unit, Nuffield Department of Population Health, University of Oxford, Oxford, UK
177 Department of Genetics, Perelman School of Medicine, University of Pennsylvania, PA, USA
178 Faculty of Medicine, University of Iceland, Reykjavik, Iceland
179 Departments of Neurology and Public Health Sciences, University of Virginia School of Medicine, Charlottesville, VA, USA
180 Department of Neurology, Boston University School of Medicine, Boston, MA, USA
181 Human Genetics Center, University of Texas Health Science Center at Houston, Houston, TX, USA
182 Center for Genomic Medicine, Kyoto University Graduate School of Medicine, Kyoto, Japan
183 Munich Cluster for Systems Neurology (SyNergy), Munich, Germany
184 German Center for Neurodegenerative Diseases (DZNE), Munich, Germany
185 Boston University School of Medicine, Boston, MA, USA
186 University of Kentucky College of Public Health, Lexington, KY, USA
187 University of Newcastle and Hunter Medical Research Institute, New Lambton, Australia
188 Univ. Montpellier, Inserm, U1061, Montpellier, France
189 Centre for Research in Environmental Epidemiology, Barcelona, Spain
190 Department of Neurology, Università degli Studi di Perugia, Umbria, Italy
191 Department of Medicine, University of Maryland School of Medicine, Baltimore, MD, USA
192 Broad Institute, Cambridge, MA, USA
193 Univ. Bordeaux, Inserm, Bordeaux Population Health Research Center, UMR 1219, Bordeaux, France
194 Bordeaux University Hospital, Department of Neurology, Memory Clinic, Bordeaux, France
195 Neurovascular Research Laboratory. Vall d'Hebron Institut of Research, Neurology and Medicine Departments-Universitat Autònoma de Barcelona. Vall d’Hebrón Hospital, Barcelona, Spain
196 University Medicine Greifswald, Department of Internal Medicine B, Greifswald, Germany
197 DZHK, Greifswald, Germany
198 Robertson Center for Biostatistics, University of Glasgow, Glasgow, UK
199 Hero DMC Heart Institute, Dayanand Medical College & Hospital, Ludhiana, India
200 Atherosclerosis Research Unit, Department of Medicine Solna, Karolinska Institutet, Stockholm, Sweden
201 Karolinska Institutet, Stockholm, Sweden
202 Division of Emergency Medicine, and Department of Neurology, Washington University School of Medicine, St. Louis, MO, USA

203 Tohoku Medical Megabank Organization, Sendai, Japan
204 Department of Psychiatry, Washington University School of Medicine, St. Louis, MO, USA
205 Department of Public Health and Caring Sciences / Geriatrics, Uppsala University, Uppsala, Sweden
206 Epidemiology and Prevention Group, Center for Public Health Sciences, National Cancer Center, Tokyo, Japan
207 Department of Internal Medicine and the Center for Clinical and Translational Science, The Ohio State University, Columbus, OH, USA
208 Institute of Neuroscience and Physiology, the Sahlgrenska Academy at University of Gothenburg, Goteborg, Sweden
209 Department of Basic and Clinical Neurosciences, King's College London, London, UK
210 Department of Health Care Administration and Management, Graduate School of Medical Sciences, Kyushu University, Japan
211 Department of Medicine and Clinical Science, Graduate School of Medical Sciences, Kyushu University, Japan
212 Landspitali National University Hospital, Departments of Neurology & Radiology, Reykjavik, Iceland
213 Department of Neurology, Heidelberg University Hospital, Germany
214 Department of Neurology, Erasmus University Medical Center
215 Hospital Universitari Mutua Terrassa, Terrassa (Barcelona), Spain
216 Albert Einstein College of Medicine, Montefiore Medical Center, New York, NY, USA
217 John Hunter Hospital, Hunter Medical Research Institute and University of Newcastle, Newcastle, NSW, Australia
218 Centre for Prevention of Stroke and Dementia, Nuffield Department of Clinical Neurosciences, University of Oxford, UK
219 Department of Medical Sciences, Uppsala University, Uppsala, Sweden
220 Genetic and Genomic Epidemiology Unit, Wellcome Trust Centre for Human Genetics, University of Oxford, Oxford, UK
221 The Wellcome Trust Centre for Human Genetics, Oxford, UK
222 Beth Israel Deaconess Medical Center, Boston, MA, USA
223 Wake Forest School of Medicine, Wake Forest, NC, USA
224 Department of Neurology, University of Pittsburgh, Pittsburgh, PA, USA
225 BioBank Japan, Laboratory of Clinical Sequencing, Department of Computational biology and medical Sciences, Graduate school of Frontier Sciences, The University of Tokyo, Tokyo, Japan
226 Neurovascular Research Laboratory, Vall d'Hebron Institut of Research, Neurology and Medicine Departments-Universitat Autònoma de Barcelona. Vall d’Hebrón Hospital, Barcelona, Spain
227 Department of Biostatistics, University of Liverpool, Liverpool, UK
228 Wellcome Trust Centre for Human Genetics, University of Oxford, Oxford, UK
229 Institute of Genetic Epidemiology, Helmholtz Zentrum München - German Research Center for Environmental Health, Neuherberg, Germany
230 Department of Medicine I, Ludwig-Maximilians-Universität, Munich, Germany

231 DZHK (German Centre for Cardiovascular Research), partner site Munich Heart Alliance, Munich, Germany
232 Department of Cerebrovascular Diseases, Fondazione IRCCS Istituto Neurologico “Carlo Besta”, Milano, Italy
233 Karolinska Institutet, MEB, Stockholm, Sweden
234 University of Tartu, Estonian Genome Center, Tartu, Estonia, Tartu, Estonia
235 Department of Clinical and Experimental Sciences, Neurology Clinic, University of Brescia, Italy
236 Translational Genomics Unit, Department of Oncology, IRCCS Istituto di Ricerche Farmacologiche Mario Negri, Milano, Italy
237 Department of Genetics, Microbiology and Statistics, University of Barcelona, Barcelona, Spain
238 Psychiatric Genetics Unit, Group of Psychiatry, Mental Health and Addictions, Vall d’Hebron Research Institute (VHIR), Universitat Autònoma de Barcelona, Biomedical Network Research Centre on Mental Health (CIBERSAM), Barcelona, Spain
239 Department of Neurology, IMIM-Hospital del Mar, and Universitat Autònoma de Barcelona, Spain
240 IMIM (Hospital del Mar Medical Research Institute), Barcelona, Spain
241 National Institute for Health Research Comprehensive Biomedical Research Centre, Guy's & St. Thomas' NHS Foundation Trust and King's College London, London, UK
242 Division of Health and Social Care Research, King's College London, London, UK
243 FIMM-Institute for Molecular Medicine Finland, Helsinki, Finland
244 THL-National Institute for Health and Welfare, Helsinki, Finland
245 Iwate Tohoku Medical Megabank Organization, Iwate Medical University, Iwate, Japan
246 BHF Glasgow Cardiovascular Research Centre, Faculty of Medicine, Glasgow, UK
247 deCODE Genetics/Amgen, Inc., Reykjavik, Iceland
248 Icelandic Heart Association, Reykjavik, Iceland
249 Institute of Biomedicine, the Sahlgrenska Academy at University of Gothenburg, Goteborg, Sweden
250 Department of Epidemiology, University of Maryland School of Medicine, Baltimore, MD, USA
251 Institute of Cardiovascular and Medical Sciences, Faculty of Medicine, University of Glasgow, Glasgow, UK
252 Chair of Genetic Epidemiology, IBE, Faculty of Medicine, LMU Munich, Germany
253 Division of Epidemiology and Prevention, Aichi Cancer Center Research Institute, Nagoya, Japan
254 Department of Epidemiology, Nagoya University Graduate School of Medicine, Nagoya, Japan
255 University Medicine Greifswald, Institute for Community Medicine, SHIP-KEF, Greifswald, Germany
256 Department of Neurology, Caen University Hospital, Caen, France

257 University of Caen Normandy, Caen, France
258 Department of Internal Medicine, Erasmus University Medical Center, Rotterdam, Netherlands
259 Landspitali University Hospital, Reykjavik, Iceland
260 Survey Research Center, University of Michigan, Ann Arbor, MI, USA
261 University of Virginia Department of Neurology, Charlottesville, VA, USA

Supplemental Tables

Supplementary Table I: SNP-platelet count associations (n=166,066) (Astle et al, 2016)							
**SNP**	**EA**	**EAF**	**GX**	**GX SE**	**p-value**	**R²**	**F**
**rs10048745**	A	0.26	0.0293	0.0043	5.29E-12	3.31E-04	55
**rs10058074**	A	0.47	-0.0317	0.0036	2.91E-18	5.01E-04	83
**rs10075570**	A	0.25	-0.0278	0.0042	3.79E-11	2.90E-04	48
**rs10199109**	T	0.69	-0.0345	0.0039	1.98E-18	5.09E-04	85
**rs10220411**	G	0.26	0.0330	0.0042	2.00E-15	4.20E-04	70
**rs1034564**	T	0.29	0.0272	0.0040	1.49E-11	3.02E-04	50
**rs10466905**	A	0.19	0.0276	0.0047	3.16E-09	2.36E-04	39
**rs1047891**	A	0.32	-0.0342	0.0039	2.25E-18	5.05E-04	84
**rs1050316**	T	0.65	-0.0254	0.0038	3.61E-11	2.93E-04	49
**rs10514301**	T	0.12	0.0394	0.0057	3.01E-12	3.23E-04	54
**rs1059196**	T	0.35	-0.0305	0.0043	1.06E-12	4.25E-04	71
**rs1060431**	A	0.07	0.0636	0.0070	1.15E-19	5.51E-04	92
**rs10761741**	T	0.42	0.0769	0.0037	3.12E-96	2.88E-03	480
**rs10769960**	C	0.44	-0.0331	0.0037	6.70E-19	5.40E-04	90
**rs10811664**	A	0.16	-0.0587	0.0050	1.62E-31	9.10E-04	151
**rs10820606**	C	0.23	0.0499	0.0044	3.83E-30	8.90E-04	148
**rs10893909**	T	0.25	-0.0317	0.0042	5.21E-14	3.79E-04	63
**rs10940072**	A	0.40	-0.0267	0.0037	9.73E-13	3.43E-04	57
**rs10974808**	G	0.14	0.1218	0.0052	1.09E-122	3.67E-03	612
**rs10984466**	G	0.36	0.0378	0.0038	2.02E-23	6.60E-04	110
**rs11071720**	C	0.70	0.0496	0.0040	1.39E-35	1.03E-03	172
**rs11082304**	T	0.51	-0.0507	0.0036	7.50E-44	1.28E-03	213
**rs11083766**	C	0.34	-0.0442	0.0039	2.76E-30	8.75E-04	145
**rs11121845**	T	0.46	-0.0391	0.0037	1.04E-25	7.61E-04	126
**rs11142444**	G	0.44	-0.0239	0.0037	6.83E-11	2.81E-04	47
**rs11175492**	G	0.10	0.0579	0.0061	3.42E-21	5.98E-04	99
**rs111941366**	T	0.45	-0.0432	0.0037	6.44E-32	9.25E-04	154
**rs11217191**	A	0.20	0.0302	0.0046	4.52E-11	2.90E-04	48
**rs11240408**	T	0.62	-0.0465	0.0038	1.35E-34	1.01E-03	168
**rs112790992**	C	0.32	0.0304	0.0039	1.24E-14	3.98E-04	66
**rs113128512**	C	0.09	-0.0384	0.0063	1.14E-09	2.45E-04	41
**rs114694170**	C	0.06	0.1632	0.0079	9.00E-96	2.96E-03	492
**rs115487693**	C	0.04	-0.0831	0.0088	3.84E-21	5.88E-04	98
**rs11553699**	G	0.14	-0.0801	0.0056	1.02E-45	1.51E-03	252
**rs1155577**	T	0.49	0.0217	0.0036	2.57E-09	2.35E-04	39
**rs11556924**	T	0.39	0.0264	0.0037	1.67E-12	3.30E-04	55
**rs11559982**	G	0.56	0.0571	0.0037	2.35E-54	1.61E-03	267
**rs11562010**	A	0.44	0.0264	0.0037	8.84E-13	3.44E-04	57
**rs1158570**	C	0.52	0.0229	0.0036	3.41E-10	2.62E-04	43
**rs11604127**	T	0.24	0.0931	0.0043	3.60E-103	3.12E-03	520
**rs116052829**	T	0.10	0.0376	0.0060	2.80E-10	2.65E-04	44
**rs11653357**	A	0.17	0.0640	0.0048	1.40E-40	1.18E-03	196
**rs11734099**	A	0.18	0.0574	0.0048	6.72E-33	9.57E-04	159
**rs1182180**	T	0.43	0.0292	0.0037	2.30E-15	4.20E-04	70

**rs11841319**	T	0.10	-0.0659	0.0061	2.02E-27	8.00E-04	133
**rs1190545**	C	0.74	0.0359	0.0042	4.81E-18	5.02E-04	83
**rs11993146**	A	0.23	-0.0266	0.0043	6.50E-10	2.53E-04	42
**rs11995702**	G	0.32	-0.0232	0.0039	2.73E-09	2.36E-04	39
**rs12005199**	A	0.31	0.1126	0.0041	2.07E-163	5.40E-03	902
**rs12052715**	G	0.72	-0.0344	0.0041	2.72E-17	4.73E-04	79
**rs12096438**	T	0.53	-0.0224	0.0036	6.70E-10	2.50E-04	42
**rs12458093**	G	0.48	0.0229	0.0038	1.92E-09	2.61E-04	43
**rs12459847**	C	0.26	-0.0516	0.0042	1.54E-34	1.01E-03	168
**rs12491785**	T	0.61	-0.0294	0.0037	3.91E-15	4.12E-04	68
**rs1260326**	C	0.61	-0.0386	0.0037	2.31E-25	7.12E-04	118
**rs12608697**	A	0.41	0.0305	0.0037	2.71E-16	4.50E-04	75
**rs12976598**	A	0.06	0.0512	0.0075	6.97E-12	3.16E-04	52
**rs1331308**	C	0.50	0.0330	0.0037	1.96E-19	5.45E-04	91
**rs1354034**	C	0.60	0.1379	0.0037	9.05E-301	9.10E-03	1526
**rs141759085**	G	0.08	0.0480	0.0067	9.42E-13	3.42E-04	57
**rs148440689**	A	0.02	0.0978	0.0119	1.70E-16	4.48E-04	74
**rs149290349**	A	0.08	-0.0827	0.0071	9.46E-32	9.60E-04	160
**rs150568286**	A	0.01	-0.1443	0.0166	3.78E-18	5.18E-04	86
**rs1506636**	G	0.68	0.0290	0.0039	9.01E-14	3.68E-04	61
**rs151233**	T	0.13	0.0648	0.0054	4.28E-33	9.55E-04	159
**rs1538970**	A	0.23	-0.0394	0.0044	3.99E-19	5.55E-04	92
**rs1555405**	A	0.25	-0.0505	0.0042	8.97E-33	9.52E-04	158
**rs1631677**	G	0.14	0.0474	0.0052	1.17E-19	5.51E-04	92
**rs16977972**	T	0.14	0.0449	0.0055	3.14E-16	4.97E-04	83
**rs16979901**	G	0.10	0.0421	0.0060	2.37E-12	3.27E-04	54
**rs1704413**	G	0.11	-0.0343	0.0059	5.88E-09	2.35E-04	39
**rs17145750**	T	0.16	-0.0294	0.0050	3.07E-09	2.33E-04	39
**rs1716505**	G	0.32	0.0440	0.0040	2.23E-28	8.46E-04	141
**rs174548**	G	0.31	0.0386	0.0039	5.46E-23	6.44E-04	107
**rs17572109**	A	0.23	0.0388	0.0044	7.46E-19	5.36E-04	89
**rs17580**	A	0.05	0.0500	0.0085	4.39E-09	2.30E-04	38
**rs17708984**	A	0.29	0.0328	0.0041	6.04E-16	4.40E-04	73
**rs17758695**	T	0.03	-0.0691	0.0107	1.20E-10	2.79E-04	46
**rs17825630**	A	0.15	0.0500	0.0055	1.06E-19	6.25E-04	104
**rs1799945**	G	0.15	-0.0351	0.0051	5.28E-12	3.14E-04	52
**rs183725**	C	0.77	0.0260	0.0044	4.63E-09	2.41E-04	40
**rs1887430**	A	0.59	0.0703	0.0037	3.20E-80	2.39E-03	398
**rs192022**	G	0.45	0.0304	0.0038	1.06E-15	4.59E-04	76
**rs2015599**	A	0.46	-0.0445	0.0036	3.74E-34	9.81E-04	163
**rs2068888**	A	0.45	-0.0237	0.0037	9.35E-11	2.79E-04	46
**rs2070667**	A	0.05	-0.0550	0.0085	1.12E-10	2.80E-04	46
**rs2075672**	G	0.63	0.0277	0.0038	1.81E-13	3.60E-04	60
**rs2078064**	A	0.11	0.0380	0.0058	6.97E-11	2.83E-04	47
**rs210142**	C	0.70	0.1020	0.0040	1.19E-144	4.35E-03	726
**rs214053**	C	0.44	-0.0479	0.0037	4.23E-39	1.13E-03	188

**rs216191**	T	0.65	0.0370	0.0038	2.52E-22	6.26E-04	104
**rs2235989**	T	0.44	0.0280	0.0037	1.63E-14	3.88E-04	64
**rs2255531**	A	0.35	-0.0242	0.0038	2.56E-10	2.66E-04	44
**rs2283847**	T	0.55	-0.0255	0.0038	2.60E-11	3.20E-04	53
**rs2284344**	C	0.52	-0.0215	0.0037	5.15E-09	2.31E-04	38
**rs2331174**	A	0.43	-0.0363	0.0037	7.59E-23	6.47E-04	108
**rs2411233**	G	0.44	0.0283	0.0037	2.43E-14	3.95E-04	66
**rs2448490**	A	0.36	-0.0286	0.0038	4.96E-14	3.79E-04	63
**rs2518683**	G	0.15	-0.0330	0.0052	2.71E-10	2.82E-04	47
**rs2523673**	C	0.46	-0.0418	0.0037	2.33E-30	8.68E-04	144
**rs2700937**	T	0.46	0.0216	0.0037	3.79E-09	2.32E-04	38
**rs2736100**	A	0.50	-0.0336	0.0036	2.95E-20	5.63E-04	94
**rs2810491**	C	0.26	0.0399	0.0042	1.81E-21	6.08E-04	101
**rs2836441**	A	0.85	-0.0435	0.0052	3.44E-17	4.78E-04	79
**rs28550009**	G	0.12	0.0737	0.0057	3.61E-38	1.13E-03	188
**rs2862064**	G	0.83	-0.0297	0.0048	7.82E-10	2.51E-04	42
**rs2894802**	G	0.57	-0.0280	0.0037	2.73E-14	3.83E-04	64
**rs290268**	G	0.46	0.0220	0.0036	1.56E-09	2.40E-04	40
**rs2932536**	A	0.53	-0.0346	0.0036	2.00E-21	5.96E-04	99
**rs2958137**	G	0.52	-0.0356	0.0037	4.02E-22	6.32E-04	105
**rs2975009**	T	0.47	0.0381	0.0038	6.98E-24	7.22E-04	120
**rs3184504**	C	0.52	-0.1039	0.0036	6.03E-180	5.39E-03	900
**rs34038797**	G	0.49	-0.0297	0.0038	5.72E-15	4.41E-04	73
**rs342292**	G	0.45	-0.0716	0.0037	2.80E-85	2.54E-03	422
**rs34536443**	C	0.05	-0.0614	0.0086	7.28E-13	3.43E-04	57
**rs34623301**	A	0.22	0.0663	0.0044	1.83E-50	1.49E-03	247
**rs34667100**	C	0.49	-0.0460	0.0036	1.62E-36	1.06E-03	175
**rs34950321**	T	0.02	-0.0876	0.0137	1.63E-10	2.72E-04	45
**rs35427**	G	0.38	-0.0256	0.0039	3.99E-11	3.09E-04	51
**rs35430985**	A	0.27	-0.0254	0.0041	6.91E-10	2.55E-04	42
**rs36109901**	C	0.27	0.0565	0.0041	2.81E-43	1.27E-03	211
**rs3731211**	A	0.72	0.0411	0.0041	5.21E-24	6.78E-04	113
**rs3741404**	C	0.36	-0.0224	0.0038	3.81E-09	2.30E-04	38
**rs3747207**	A	0.21	-0.0345	0.0045	9.10E-15	4.01E-04	67
**rs3804749**	T	0.59	-0.0413	0.0037	2.39E-28	8.22E-04	137
**rs3809114**	A	0.56	-0.0246	0.0037	2.57E-11	2.98E-04	49
**rs3809272**	A	0.30	-0.0945	0.0040	3.92E-126	3.78E-03	630
**rs3819299**	G	0.06	0.0936	0.0079	4.08E-32	9.22E-04	153
**rs3844535**	G	0.70	0.0237	0.0040	2.63E-09	2.36E-04	39
**rs3865444**	A	0.32	-0.0225	0.0039	7.39E-09	2.21E-04	37
**rs409950**	A	0.18	0.0646	0.0049	4.93E-39	1.24E-03	206
**rs41315846**	C	0.48	0.0575	0.0037	1.88E-54	1.65E-03	274
**rs4148435**	A	0.92	0.0747	0.0066	9.18E-30	8.58E-04	143
**rs4272720**	G	0.24	-0.0350	0.0043	4.16E-16	4.42E-04	74
**rs429358**	C	0.14	-0.0302	0.0051	3.26E-09	2.22E-04	37
**rs4377346**	C	0.25	0.0362	0.0046	3.53E-15	4.95E-04	82

**rs4388979**	T	0.58	-0.0435	0.0037	7.37E-32	9.21E-04	153
**rs4411786**	C	0.26	-0.0459	0.0041	1.63E-28	8.18E-04	136
**rs4432538**	A	0.51	-0.0242	0.0037	3.78E-11	2.92E-04	49
**rs4455005**	A	0.64	-0.0302	0.0038	3.57E-15	4.20E-04	70
**rs4470077**	G	0.19	0.0293	0.0047	3.05E-10	2.66E-04	44
**rs4631704**	T	0.61	0.0238	0.0038	2.41E-10	2.70E-04	45
**rs4670779**	T	0.32	-0.0244	0.0040	8.64E-10	2.58E-04	43
**rs4699154**	C	0.69	0.0269	0.0040	1.08E-11	3.09E-04	51
**rs4709819**	A	0.41	0.0317	0.0037	1.74E-17	4.85E-04	81
**rs4711890**	G	0.27	0.0305	0.0041	1.44E-13	3.65E-04	61
**rs4783186**	C	0.88	-0.0422	0.0056	3.07E-14	3.84E-04	64
**rs4846217**	T	0.13	-0.0361	0.0055	5.42E-11	2.86E-04	48
**rs4907622**	C	0.53	-0.0304	0.0037	8.13E-17	4.61E-04	77
**rs4925750**	C	0.31	0.0327	0.0039	9.43E-17	4.59E-04	76
**rs4937127**	G	0.52	-0.0320	0.0036	1.86E-18	5.10E-04	85
**rs4937333**	C	0.53	-0.0322	0.0037	6.07E-18	5.18E-04	86
**rs4965426**	A	0.14	-0.0365	0.0052	2.21E-12	3.27E-04	54
**rs553749201**	A	0.05	-0.0654	0.0083	2.32E-15	4.42E-04	73
**rs55707100**	T	0.03	0.1143	0.0114	1.83E-23	6.64E-04	110
**rs56036086**	A	0.14	-0.0511	0.0053	3.78E-22	6.23E-04	103
**rs56043070**	A	0.07	-0.1388	0.0070	2.66E-86	2.55E-03	425
**rs56125409**	G	0.12	-0.0506	0.0056	1.65E-19	5.54E-04	92
**rs57274573**	T	0.17	0.0280	0.0049	8.08E-09	2.21E-04	37
**rs58530613**	C	0.11	0.0346	0.0058	3.27E-09	2.38E-04	40
**rs59739601**	G	0.08	-0.0551	0.0069	1.29E-15	4.29E-04	71
**rs59865663**	A	0.20	0.0408	0.0046	9.30E-19	5.41E-04	90
**rs6060983**	C	0.30	-0.0334	0.0040	5.58E-17	4.67E-04	78
**rs619460**	A	0.62	-0.0237	0.0037	2.48E-10	2.65E-04	44
**rs6425521**	A	0.80	-0.0652	0.0046	2.49E-46	1.36E-03	225
**rs6445967**	C	0.32	-0.0305	0.0039	4.68E-15	4.06E-04	67
**rs655029**	A	0.71	0.0735	0.0041	9.37E-73	2.22E-03	369
**rs655641**	G	0.81	0.0266	0.0045	4.10E-09	2.20E-04	37
**rs6556471**	C	0.68	-0.0462	0.0039	2.96E-32	9.31E-04	155
**rs670179**	A	0.57	0.0282	0.0037	3.14E-14	3.90E-04	65
**rs6756513**	A	0.28	-0.0251	0.0041	6.87E-10	2.55E-04	42
**rs6925716**	C	0.51	0.0300	0.0037	1.89E-16	4.51E-04	75
**rs6961069**	T	0.40	0.0261	0.0038	4.13E-12	3.27E-04	54
**rs6993770**	T	0.29	-0.0700	0.0040	1.02E-67	2.00E-03	333
**rs7146395**	C	0.67	-0.0227	0.0039	4.79E-09	2.27E-04	38
**rs71633359**	C	0.32	0.0251	0.0040	5.57E-10	2.73E-04	45
**rs7178196**	A	0.17	-0.0345	0.0049	1.90E-12	3.31E-04	55
**rs7249921**	T	0.42	-0.0237	0.0037	2.58E-10	2.73E-04	45
**rs73000929**	A	0.04	-0.0919	0.0098	5.84E-21	6.08E-04	101
**rs73000965**	A	0.32	-0.0281	0.0039	8.58E-13	3.43E-04	57
**rs73109811**	T	0.20	0.0407	0.0047	5.75E-18	5.27E-04	88
**rs75080135**	C	0.18	0.1019	0.0048	5.82E-101	3.03E-03	505

**rs75107793**	A	0.07	0.1157	0.0071	2.28E-59	1.80E-03	300
**rs75501914**	A	0.07	0.0494	0.0074	2.94E-11	2.99E-04	50
**rs7585866**	G	0.35	0.0234	0.0038	1.00E-09	2.49E-04	41
**rs75967349**	G	0.05	-0.0554	0.0085	5.61E-11	2.91E-04	48
**rs7641175**	A	0.78	0.0458	0.0044	2.40E-25	7.18E-04	119
**rs7665147**	A	0.19	-0.0317	0.0047	1.66E-11	3.04E-04	51
**rs7696658**	T	0.48	0.0215	0.0037	4.17E-09	2.30E-04	38
**rs77300440**	T	0.08	0.0686	0.0067	1.50E-24	6.98E-04	116
**rs77320796**	G	0.26	0.0268	0.0042	1.35E-10	2.76E-04	46
**rs7776054**	G	0.26	0.1192	0.0042	4.09E-181	5.47E-03	914
**rs7788849**	A	0.90	-0.0443	0.0062	6.17E-13	3.46E-04	57
**rs7811142**	T	0.20	0.0358	0.0046	5.57E-15	4.04E-04	67
**rs78265569**	A	0.09	-0.0458	0.0065	1.59E-12	3.41E-04	57
**rs7833924**	G	0.43	0.0439	0.0037	7.45E-33	9.44E-04	157
**rs78565404**	T	0.05	0.1420	0.0085	5.36E-63	2.05E-03	342
**rs78909033**	A	0.14	0.0653	0.0053	1.27E-34	1.00E-03	167
**rs79287178**	A	0.03	0.0642	0.0108	2.44E-09	2.69E-04	45
**rs7950696**	C	0.45	0.0274	0.0037	6.55E-14	3.71E-04	62
**rs79936776**	G	0.03	0.0787	0.0102	1.35E-14	3.99E-04	66
**rs80012730**	C	0.07	-0.0438	0.0070	4.16E-10	2.65E-04	44
**rs80054178**	C	0.02	0.1248	0.0123	2.77E-24	6.88E-04	114
**rs8037137**	C	0.13	-0.0354	0.0054	7.81E-11	2.84E-04	47
**rs8073060**	A	0.29	0.0300	0.0040	8.87E-14	3.70E-04	61
**rs8137128**	T	0.56	-0.0418	0.0037	3.73E-29	8.61E-04	143
**rs8176747**	G	0.06	-0.0546	0.0075	3.58E-13	3.51E-04	58
**rs853195**	A	0.64	-0.0240	0.0038	2.24E-10	2.66E-04	44
**rs926326**	G	0.77	-0.0475	0.0043	8.63E-28	7.92E-04	132
**rs9267098**	G	0.27	-0.0498	0.0043	7.99E-31	9.73E-04	162
**rs928391**	C	0.27	-0.0260	0.0041	2.45E-10	2.66E-04	44
**rs9402633**	T	0.21	0.0328	0.0045	2.26E-13	3.58E-04	59
**rs9462031**	T	0.16	-0.0432	0.0051	2.91E-17	4.89E-04	81
**rs9524862**	A	0.49	0.0351	0.0037	9.04E-22	6.16E-04	102
**rs9704108**	C	0.93	0.0535	0.0078	5.63E-12	3.83E-04	64
**rs9809116**	G	0.41	0.0265	0.0037	1.32E-12	3.39E-04	56
**rs9810259**	G	0.42	-0.0409	0.0037	1.03E-28	8.17E-04	136
**rs9908158**	C	0.35	0.0312	0.0038	3.63E-16	4.41E-04	73
**rs9974653**	C	0.32	-0.0295	0.0039	7.54E-14	3.78E-04	63
EA: effect allele; EAF: effect allele frequency; F: F-statistic; GX: the association between the SNP and platelet count; R2: the proportion of variability in platelet count explained by the SNP; SE: standard error; SNP: single nucleotide polymorphism.							

Supplementary Table II: Potentially pleiotropic associations between SNPs and risk of CVD, independent of their effect on platelet count. Pleiotropic effects were searched for using the PhenoScanner database (available online at: http://www.phenoscanner.medschl.cam.ac.uk/phenoscanner) (Staley et al, 2016)		
SNP	Pleiotropic effect	Reference(s)
rs10048745		
rs10058074	Height; inflammatory bowel disease	GIANT, 2014; IBDGC, 2015
rs10075570		
rs10199109		
rs10220411		
rs1034564		
rs10466905		
rs1047891	Creatinine; fibrinogen; high density lipoprotein cholesterol; homocysteine	CKD-Gen, 2016; GLGC, 2013; Danik et al, 2009; Lange et al, 2010
rs1050316	Migraine without aura	Freilnger et al, 2012
rs10514301		
rs1059196		
rs1060431		
rs10761741	Alkaline phosphatase; platelet aggregation in response to epinephrine; triglycerides; years of educational attainment	GLGC, 2013; SSGAC, 2016; Johnson et al, 2010; Yuan et al, 2008
rs10769960		
rs10811664		
rs10820606		
rs10893909		
rs10940072		
rs10974808	Mean cell hemoglobin concentration; mean cell volume	Ganesh et al, 2009; van der Harst et al, 2012
rs10984466		
rs11071720	Mean platelet volume	Soranzo et al, 2009
rs11082304	Height	GIANT, 2014

rs11083766		
rs11121845		
rs11142444		
rs11175492		
rs111941366		
rs11217191		
rs11240408		
rs112790992		
rs113128512		
rs114694170		
rs115487693	Rheumatoid arthritis	Okada et al, 2014
rs11553699		
rs1155577		
rs11556924	Coronary artery disease; myocardial infarction	CARDIoGRAMplusC4D, 2015
rs11559982		
rs11562010		
rs1158570		
rs11604127		
rs116052829		
rs11653357		
rs11734099		
rs1182180		
rs11841319		
rs1190545	Height	GIANT, 2014
rs11993146		
rs11995702		
rs12005199		
rs12052715		
rs12096438		
rs12458093		
rs12459847		

rs12491785		
rs1260326	Age at menopause; albumin; C-reactive protein; cardiovascular disease risk factors; cholesterol; chronic kidney disease; clotting factor VII, cretinine; fasting glucose; fasting insulin; gamma glutamyl transferase; gout; height; lipid metabolism phenotypes; triglycerides; type 2 diabetes; urate	Asselbergs et al, 2012; Avery et al, 2011; Barber et al, 2010; Chambers et al, 2011; Chasman et al, 2009; CKD-Gen, 2016; CKD-Gen, 2010; Dehghan et al, 2011; DIAGRAM, 2015; Elbers et al, 2012; Franceschini et al, 2012; GIANT, 2014; GLGC, 2013; Johansen et al, 2010; Kamatani et al, 2010; Kathiresan et al, 2008; Kolz et al, 2009; MAGIC, 2012; Matuso et al 2015; Middelberg et al, 2011; Musunuru et al, 2012; Pattaro et al, 2012; ReproGen, 2015; Sabatti et al, 2008; Shah et al, 2013; Smith et al, 2010; Tang et al, 2010; Taylor et al, 2011; Weissglas Volkov et al, 2013; Willer et al, 2008; Yang et al, 2010
rs12608697		
rs12976598		
rs1331308	Mean cell hemoglobin concentration; mean cell volume	van der Harst, 2012
rs1354034	Mean platelet volume, red blood cell traits, white blood cell count	Gieger et al, 2011; Shameer et al, 2013; Li et al, 2012
rs141759085		
rs148440689		
rs149290349	Inflammatory bowel disease, type 2 diabetes	DIAGRAM, 2015; IBDGC, 2015
rs150568286		
rs1506636		
rs151233		
rs1538970		
rs1555405		
rs1631677		
rs16977972		
rs16979901		
rs1704413		

rs17145750	Gamma glutamyl transferase; high density lipoprotein; triglycerides; serum urate; very low density lipoprotein cholesterol	GLGC, 2013; GUGC, 2012; Chambers, 2011; Kettunen et al, 2012
rs1716505		
rs174548	Fasting glucose; heart rate; high density lipoprotein cholesterol; low density lipoprotein cholesterol; total cholesterol; triglycerides	GLGC, 2010; HRGene, 2013; MAGIC, 2010; Waterworth, 2010
rs17572109	Height	GIANT, 2014
rs17580		
rs17708984		
rs17758695		
rs17825630		
rs1799945	Blood pressure; iron status biomarkers	ICBP, 2011; Pichler et al, 2011; Chambers et al, 2009; Ganesh et al, 2009; Johnson et al, 2011; van der Harst et al, 2012
rs183725		
rs1887430		
rs192022		
rs2015599	Mean platelet volume	Gieger et al, 2011
rs2068888	Creatinine; triglycerides	CKD-Gen, 2016; GLGC, 2013
rs2070667		
rs2075672	Mean cell hemoglobin; mean cell hemoglobin concentration; mean cell volume	van der Harst et al, 2012
rs2078064		
rs210142	Chronic lymphocytic leukemia	Berndt et al, 2013; Slager et al, 2012
rs214053		
rs216191	Aortic root size; schizophrenia	PGC, 2014; Vasan et al, 2009
rs2235989		
rs2255531	C-reactive protein	Wu et al, 2011

rs2283847		
rs2284344		
rs2331174		
rs2411233		
rs2448490		
rs2518683		
rs2523673		
rs2700937		
rs2736100	Gliona; lung cancer; red blood cell count; testicular cancer	Chung et al, 2013; GliomaScan, 2012; Hsiung et al, 2010; Hu et al, 2011; Kamatani et al, 2010; Lan et al, 2012; Landi et al, 2009; Miki et al, 2010; Sanson et a, 2011; Shete et al, 2009; Shiraishi et al, 2012; Timofeeva et al, 2012; Turnbull et al, 2010; Walsh et al, 2014; Wrensch et al, 2009; Yang et al, 2011
rs2810491		
rs2836441		
rs28550009		
rs2862064		
rs2894802		
rs290268		
rs2932536		
rs2958137		
rs2975009		
rs3184504	Blood pressure; cholesterol; coronary artery disease; estimated glomerular filtration rate; high density lipoprotein; hypothyroidism; low density lipoprotein; myocardial infarction; red blood cell traits; rheumatoid arthritis; type 1 diabetes; urate	Asselbergs et al, 2012; Barrett et al, 2009; BPExome, 2016; Bradfiel et al, 2011; CARDIoGRAMplusC4D, 2015; CKD-Gen, 2016; Eriksson et al, 2012; Fox et al, 2011; Ganesh et al, 2009; Gieger et al, 2011; GLGC, 2013, GUGC, 2012; Howson et al, 2017; ICBP, 2011; Lettre et al, 2011; Levy et al, 2009; Plagnol et al, 2011; Soranzo et al, 2009; van der Harst et al, 2012; Wain et al, 2011; Zhernakova et al, 2011
rs34038797		

rs342292		
rs34536443	Rheumatoid arthitis; primary biliary cirrhosis	Okada et al, 2014; Eyre et al, 2012; Liu et al, 2012
rs34623301		
rs34667100		
rs34950321		
rs35427		
rs35430985	Type 2 diabetes	DIAGRAM, 2015
rs36109901		
rs3731211		
rs3741404		
rs3747207		
rs3804749		
rs3809114	Urate	GUGC, 2012
rs3809272		
rs3819299	Rheumatoid arthritis	Okada et al, 2014
rs3844535		
rs3865444	Alzheimer's disease	Naj et al, 2011; Hollingworth et al, 2011
rs409950		
rs41315846		
rs4148435		
rs4272720		
rs429358	Alzheimer's disease; coronary artery disease; Lewy body disease; low density lipoprotein cholesterol change with statins; myocardial infarction	Beecham et al, 2014; CARDIoGRAMplusC4D, 2015; IGAP, 2013; Thompson et al, 2009
rs4377346		
rs4388979		
rs4411786		
rs4432538		
rs4455005		
rs4470077	Galectin 3	de Boer et al, 2012

rs4631704		
rs4670779		
rs4699154		
rs4709819	Mean cell hemoglobin concentration; mean cell volume	van der Harst et al, 2012
rs4711890		
rs4783186		
rs4846217		
rs4907622		
rs4925750		
rs4937127		
rs4937333		
rs4965426		
rs553749201		
rs55707100		
rs56036086		
rs56043070		
rs56125409		
rs57274573		
rs58530613		
rs59739601		
rs59865663		
rs6060983		
rs619460		
rs6425521		
rs6445967		
rs655029		
rs655641		
rs6556471		
rs670179		
rs6756513		
rs6925716		

rs6961069		
rs6993770	Vascular endothelial growth factor	Debette et al, 2011
rs7146395		
rs71633359		
rs7178196		
rs7249921		
rs73000929		
rs73000965		
rs73109811		
rs75080135		
rs75107793		
rs75501914		
rs7585866		
rs75967349		
rs7641175		
rs7665147		
rs7696658		
rs77300440		
rs77320796		
rs7776054	Mean cell hemoglobin; red blood cell traits	Ganesh et al, 2009; van der Harst et al, 2012
rs7788849		
rs7811142		
rs78265569		
rs7833924		
rs78565404		
rs78909033		
rs79287178		
rs7950696	Body mass index; proinsulin	GIANT, 2015; MAGIC, 2011
rs79936776		
rs80012730		
rs80054178		

rs8037137	Type 2 diabetes	DIAGRAM, 2015
rs8073060		
rs8137128		
rs8176747	Activated partial thromboplastin time; carcinoembryonic antigen; clotting factor XII; hemoglobin; red blood cell traits; von Willebrand factor	He et al, 2013; Tang et al, 2012; van der Harst et al, 2012; Williams et al, 2013
rs853195		
rs926326		
rs9267098		
rs928391		
rs9402633		
rs9462031		
rs9524862		
rs9704108		
rs9809116		
rs9810259		
rs9908158		
rs9974653		

BPExome: blood pressure exome; CARDIoGRAMplusC4D: coronary artery disease genome wide replication and meta-analysis plus the coronary artery disease genetics; CKD-GEN: chronic kidney disease genetics; CVD: cardiovascular disease; DIAGRAM: diabetes genetics replication and meta-analysis; GIANT: genetic investigation of anthropometric traits; GLGC: global lipids genetics consortium; GUGC: global rate genetics consortium; HRGene: heart rate consortium; IBDGC: inflammatory bowel disease genetics consortium; ICBP: international consortium of blood pressure; PGC: psychiatric genomics consortium; IGAP: international genomics of Alzheimer’s project; MAGIC: meta-analyses of glucose and insulin-related traits consortium; ReproGen: reproductive genetics; SNP: single nucleotide polymorphism; SSGAC: social science genetic association consortium.

Supplementary Table III: Inverse variance weighted MR estimates for platelet count and coronary artery disease (Astle et al, 2016; Nikpay et al, 2015)							
**SNP**	**EA**	**GX**	**GX SE**	**GY**	**GY SE**	**MR**	**MR SE**
**rs10048745**	A	0.0293	0.0043	-0.0126	0.0117	-0.4303	0.4029
**rs10058074**	A	-0.0317	0.0036	-0.0121	0.0103	0.3828	0.3268
**rs10075570**	A	-0.0278	0.0042	0.0027	0.0100	-0.0984	0.3597
**rs10199109**	T	-0.0345	0.0039	-0.0023	0.0108	0.0656	0.3128
**rs10220411**	G	0.0330	0.0042	-0.0209	0.0114	-0.6329	0.3541
**rs1034564**	T	0.0272	0.0040	0.0302	0.0103	1.1119	0.4139
**rs10466905**	A	0.0276	0.0047	0.0009	0.0135	0.0344	0.4900
**rs1047891**	A	-0.0342	0.0039	-0.0254	0.0108	0.7423	0.3263
**rs1050316**	T	-0.0254	0.0038	-0.0163	0.0097	0.6406	0.3945
**rs10514301**	T	0.0394	0.0057	0.0385	0.0136	0.9753	0.3713
**rs1059196**	T	-0.0305	0.0043	-0.0001	0.0133	0.0043	0.4356
**rs1060431**	A	0.0636	0.0070	-0.0150	0.0170	-0.2355	0.2681
**rs10761741**	T	0.0769	0.0037	0.0069	0.0092	0.0900	0.1202
**rs10769960**	C	-0.0331	0.0037	-0.0122	0.0119	0.3683	0.3604
**rs10811664**	A	-0.0587	0.0050	0.0285	0.0126	-0.4857	0.2183
**rs10820606**	C	0.0499	0.0044	-0.0061	0.0131	-0.1224	0.2638
**rs10893909**	T	-0.0317	0.0042	-0.0167	0.0115	0.5282	0.3681
**rs10940072**	A	-0.0267	0.0037	-0.0168	0.0095	0.6294	0.3667
**rs10974808**	G	0.1218	0.0052	-0.0093	0.0163	-0.0762	0.1337
**rs10984466**	G	0.0378	0.0038	-0.0018	0.0101	-0.0486	0.2686
**rs11071720**	C	0.0496	0.0040	-0.0055	0.0100	-0.1108	0.2012
**rs11082304**	T	-0.0507	0.0036	0.0098	0.0095	-0.1942	0.1883
**rs11083766**	C	-0.0442	0.0039	0.0224	0.0131	-0.5071	0.3006
**rs11121845**	T	-0.0391	0.0037	-0.0027	0.0095	0.0693	0.2424
**rs11142444**	G	-0.0239	0.0037	0.0139	0.0095	-0.5816	0.4052
**rs11175492**	G	0.0579	0.0061	0.0281	0.0164	0.4854	0.2872
**rs111941366**	T	-0.0432	0.0037	-0.0151	0.0117	0.3495	0.2723
**rs11217191**	A	0.0302	0.0046	-0.0029	0.0113	-0.0959	0.3732
**rs11240408**	T	-0.0465	0.0038	0.0021	0.0105	-0.0446	0.2253
**rs112790992**	C	0.0304	0.0039	-0.0048	0.0104	-0.1591	0.3427
**rs113128512**	C	-0.0384	0.0063	-0.0024	0.0167	0.0624	0.4348
**rs114694170**	C	0.1632	0.0079	0.0011	0.0255	0.0068	0.1562
**rs115487693**	C	-0.0831	0.0088	-0.0040	0.0338	0.0477	0.4070
**rs11553699**	G	-0.0801	0.0056	-0.0258	0.0194	0.3218	0.2427
**rs1155577**	T	0.0217	0.0036	0.0300	0.0092	1.3801	0.4838
**rs11556924**	T	0.0264	0.0037	-0.0726	0.0111	-2.7531	0.5729
**rs11559982**	G	0.0571	0.0037	0.0083	0.0097	0.1449	0.1703
**rs11562010**	A	0.0264	0.0037	-0.0192	0.0094	-0.7278	0.3706
**rs1158570**	C	0.0229	0.0036	-0.0048	0.0094	-0.2085	0.4099
**rs11604127**	T	0.0931	0.0043	0.0072	0.0141	0.0772	0.1512
**rs116052829**	T	0.0376	0.0060	0.0208	0.0178	0.5537	0.4822
**rs11653357**	A	0.0640	0.0048	0.0095	0.0116	0.1490	0.1809

**rs11734099**	A	0.0574	0.0048	0.0255	0.0124	0.4447	0.2199
**rs1182180**	T	0.0292	0.0037	-0.0113	0.0095	-0.3871	0.3283
**rs11841319**	T	-0.0659	0.0061	0.0036	0.0153	-0.0553	0.2318
**rs1190545**	C	0.0359	0.0042	0.0056	0.0100	0.1557	0.2784
**rs11993146**	A	-0.0266	0.0043	0.0159	0.0105	-0.5983	0.4065
**rs11995702**	G	-0.0232	0.0039	0.0103	0.0102	-0.4421	0.4461
**rs12005199**	A	0.1126	0.0041	0.0082	0.0112	0.0725	0.0996
**rs12052715**	G	-0.0344	0.0041	-0.0009	0.0105	0.0250	0.3046
**rs12096438**	T	-0.0224	0.0036	-0.0015	0.0093	0.0654	0.4162
**rs12458093**	G	0.0229	0.0038	0.0009	0.0108	0.0410	0.4740
**rs12459847**	C	-0.0516	0.0042	0.0366	0.0111	-0.7101	0.2227
**rs12491785**	T	-0.0294	0.0037	0.0220	0.0094	-0.7497	0.3328
**rs1260326**	C	-0.0386	0.0037	0.0033	0.0096	-0.0844	0.2493
**rs12608697**	A	0.0305	0.0037	-0.0012	0.0094	-0.0408	0.3069
**rs12976598**	A	0.0512	0.0075	0.0119	0.0212	0.2329	0.4146
**rs1331308**	C	0.0330	0.0037	-0.0171	0.0093	-0.5193	0.2878
**rs1354034**	C	0.1379	0.0037	0.0050	0.0096	0.0360	0.0695
**rs141759085**	G	0.0480	0.0067	0.0311	0.0172	0.6483	0.3697
**rs148440689**	A	0.0978	0.0119	0.0384	0.0325	0.3931	0.3360
**rs149290349**	A	-0.0827	0.0071	0.0611	0.0211	-0.7392	0.2623
**rs150568286**	A	-0.1443	0.0166	0.0502	0.0746	-0.3477	0.5186
**rs1506636**	G	0.0290	0.0039	0.0081	0.0104	0.2786	0.3607
**rs151233**	T	0.0648	0.0054	0.0077	0.0191	0.1183	0.2943
**rs1538970**	A	-0.0394	0.0044	0.0081	0.0114	-0.2044	0.2907
**rs1555405**	A	-0.0505	0.0042	-0.0089	0.0103	0.1761	0.2053
**rs1631677**	G	0.0474	0.0052	0.0035	0.0123	0.0740	0.2604
**rs16977972**	T	0.0449	0.0055	-0.0096	0.0137	-0.2149	0.3074
**rs16979901**	G	0.0421	0.0060	0.0287	0.0176	0.6803	0.4279
**rs17145750**	T	-0.0294	0.0050	-0.0066	0.0139	0.2235	0.4760
**rs1716505**	G	0.0440	0.0040	-0.0099	0.0103	-0.2252	0.2343
**rs174548**	G	0.0386	0.0039	-0.0194	0.0103	-0.5030	0.2713
**rs17572109**	A	0.0388	0.0044	0.0122	0.0134	0.3138	0.3462
**rs17580**	A	0.0500	0.0085	-0.0313	0.0270	-0.6250	0.5507
**rs17708984**	A	0.0328	0.0041	-0.0046	0.0099	-0.1391	0.3041
**rs17825630**	A	0.0500	0.0055	-0.0141	0.0138	-0.2816	0.2772
**rs1799945**	G	-0.0351	0.0051	0.0072	0.0142	-0.2046	0.4059
**rs1887430**	A	0.0703	0.0037	-0.0027	0.0094	-0.0381	0.1334
**rs2015599**	A	-0.0445	0.0036	-0.0017	0.0092	0.0379	0.2061
**rs2068888**	A	-0.0237	0.0037	-0.0391	0.0097	1.6482	0.4811
**rs2070667**	A	-0.0550	0.0085	-0.0272	0.0188	0.4942	0.3508
**rs2075672**	G	0.0277	0.0038	0.0009	0.0099	0.0307	0.3555
**rs2078064**	A	0.0380	0.0058	0.0140	0.0128	0.3690	0.3403
**rs210142**	C	0.1020	0.0040	-0.0070	0.0111	-0.0685	0.1088
**rs214053**	C	-0.0479	0.0037	-0.0159	0.0092	0.3325	0.1937
**rs216191**	T	0.0370	0.0038	-0.0381	0.0096	-1.0277	0.2789
**rs2235989**	T	0.0280	0.0037	-0.0066	0.0092	-0.2355	0.3315

**rs2255531**	A	-0.0242	0.0038	0.0421	0.0095	-1.7419	0.4789
**rs2283847**	T	-0.0255	0.0038	-0.0016	0.0103	0.0616	0.4050
**rs2284344**	C	-0.0215	0.0037	0.0062	0.0095	-0.2874	0.4450
**rs2331174**	A	-0.0363	0.0037	-0.0117	0.0098	0.3229	0.2716
**rs2448490**	A	-0.0286	0.0038	0.0093	0.0105	-0.3243	0.3685
**rs2518683**	G	-0.0330	0.0052	0.0305	0.0137	-0.9262	0.4398
**rs2700937**	T	0.0216	0.0037	0.0097	0.0093	0.4514	0.4378
**rs2736100**	A	-0.0336	0.0036	0.0303	0.0098	-0.9022	0.3068
**rs2810491**	C	0.0399	0.0042	0.0120	0.0102	0.2997	0.2581
**rs2836441**	A	-0.0435	0.0052	0.0041	0.0141	-0.0943	0.3237
**rs28550009**	G	0.0737	0.0057	-0.0099	0.0159	-0.1346	0.2166
**rs2862064**	G	-0.0297	0.0048	0.0181	0.0138	-0.6081	0.4735
**rs2894802**	G	-0.0280	0.0037	0.0044	0.0095	-0.1570	0.3387
**rs290268**	G	0.0220	0.0036	-0.0101	0.0092	-0.4597	0.4255
**rs2932536**	A	-0.0346	0.0036	0.0015	0.0093	-0.0441	0.2678
**rs2958137**	G	-0.0356	0.0037	-0.0106	0.0095	0.2978	0.2682
**rs3184504**	C	-0.1039	0.0036	-0.0642	0.0105	0.6181	0.1035
**rs34038797**	G	-0.0297	0.0038	0.0035	0.0094	-0.1165	0.3183
**rs342292**	G	-0.0716	0.0037	-0.0142	0.0098	0.1978	0.1372
**rs34536443**	C	-0.0614	0.0086	-0.0656	0.0359	1.0686	0.6037
**rs34623301**	A	0.0663	0.0044	0.0073	0.0122	0.1099	0.1846
**rs34667100**	C	-0.0460	0.0036	-0.0044	0.0116	0.0960	0.2534
**rs34950321**	T	-0.0876	0.0137	0.0406	0.0360	-0.4628	0.4173
**rs35427**	G	-0.0256	0.0039	0.0000	0.0101	0.0005	0.3940
**rs35430985**	A	-0.0254	0.0041	0.0062	0.0104	-0.2431	0.4094
**rs36109901**	C	0.0565	0.0041	0.0136	0.0100	0.2409	0.1786
**rs3731211**	A	0.0411	0.0041	-0.0296	0.0107	-0.7202	0.2706
**rs3741404**	C	-0.0224	0.0038	-0.0084	0.0103	0.3751	0.4668
**rs3747207**	A	-0.0345	0.0045	-0.0193	0.0111	0.5583	0.3285
**rs3778028**	A	0.1172	0.0196	0.0225	0.0318	0.1915	0.2733
**rs3804749**	T	-0.0413	0.0037	0.0024	0.0095	-0.0585	0.2295
**rs3809114**	A	-0.0246	0.0037	-0.0051	0.0097	0.2078	0.3962
**rs3809272**	A	-0.0945	0.0040	-0.0318	0.0113	0.3362	0.1203
**rs3819299**	G	0.0936	0.0079	0.0400	0.0190	0.4275	0.2065
**rs3844535**	G	0.0237	0.0040	0.0080	0.0103	0.3375	0.4386
**rs3865444**	A	-0.0225	0.0039	-0.0003	0.0105	0.0136	0.4659
**rs409950**	A	0.0646	0.0049	-0.0078	0.0132	-0.1215	0.2042
**rs41315846**	C	0.0575	0.0037	-0.0041	0.0098	-0.0709	0.1704
**rs41316003**	A	0.1654	0.0276	0.0886	0.0757	0.5357	0.4663
**rs4148435**	A	0.0747	0.0066	0.0074	0.0146	0.0988	0.1958
**rs4272720**	G	-0.0350	0.0043	-0.0104	0.0121	0.2964	0.3485
**rs429358**	C	-0.0302	0.0051	0.0909	0.0152	-3.0086	0.7150
**rs4388979**	T	-0.0435	0.0037	-0.0009	0.0097	0.0215	0.2219
**rs4411786**	C	-0.0459	0.0041	0.0275	0.0100	-0.5995	0.2253
**rs4432538**	A	-0.0242	0.0037	0.0118	0.0093	-0.4875	0.3903
**rs4470077**	G	0.0293	0.0047	0.0145	0.0119	0.4949	0.4129

**rs4631704**	T	0.0238	0.0038	-0.0307	0.0098	-1.2865	0.4586
**rs4670779**	T	-0.0244	0.0040	-0.0180	0.0103	0.7382	0.4385
**rs4699154**	C	0.0269	0.0040	-0.0129	0.0102	-0.4786	0.3858
**rs4709819**	A	0.0317	0.0037	-0.0203	0.0092	-0.6399	0.2986
**rs4711890**	G	0.0305	0.0041	-0.0111	0.0111	-0.3623	0.3661
**rs4783186**	C	-0.0422	0.0056	-0.0062	0.0149	0.1480	0.3537
**rs4846217**	T	-0.0361	0.0055	-0.0067	0.0137	0.1860	0.3798
**rs4907622**	C	-0.0304	0.0037	0.0013	0.0105	-0.0419	0.3440
**rs4925750**	C	0.0327	0.0039	0.0352	0.0100	1.0775	0.3332
**rs4937127**	G	-0.0320	0.0036	-0.0045	0.0095	0.1403	0.2973
**rs4937333**	C	-0.0322	0.0037	0.0063	0.0092	-0.1956	0.2852
**rs4965426**	A	-0.0365	0.0052	0.0011	0.0133	-0.0297	0.3647
**rs55707100**	T	0.1143	0.0114	-0.0011	0.0308	-0.0092	0.2696
**rs56036086**	A	-0.0511	0.0053	0.0043	0.0142	-0.0836	0.2777
**rs56043070**	A	-0.1388	0.0070	-0.0150	0.0230	0.1078	0.1660
**rs56125409**	G	-0.0506	0.0056	-0.0073	0.0138	0.1443	0.2731
**rs57274573**	T	0.0280	0.0049	0.0071	0.0190	0.2528	0.6811
**rs58530613**	C	0.0346	0.0058	0.0125	0.0155	0.3624	0.4509
**rs59739601**	G	-0.0551	0.0069	0.0029	0.0171	-0.0527	0.3110
**rs59865663**	A	0.0408	0.0046	0.0030	0.0141	0.0740	0.3457
**rs6060983**	C	-0.0334	0.0040	-0.0047	0.0110	0.1400	0.3301
**rs619460**	A	-0.0237	0.0037	-0.0193	0.0095	0.8153	0.4207
**rs6425521**	A	-0.0652	0.0046	0.0056	0.0115	-0.0852	0.1770
**rs6445967**	C	-0.0305	0.0039	-0.0176	0.0096	0.5789	0.3235
**rs655029**	A	0.0735	0.0041	-0.0096	0.0116	-0.1312	0.1578
**rs655641**	G	0.0266	0.0045	0.0115	0.0112	0.4309	0.4250
**rs6556471**	C	-0.0462	0.0039	0.0069	0.0099	-0.1504	0.2145
**rs670179**	A	0.0282	0.0037	-0.0091	0.0097	-0.3210	0.3464
**rs6756513**	A	-0.0251	0.0041	0.0027	0.0107	-0.1066	0.4242
**rs6925716**	C	0.0300	0.0037	-0.0049	0.0093	-0.1620	0.3087
**rs6961069**	T	0.0261	0.0038	0.0036	0.0094	0.1376	0.3622
**rs6993770**	T	-0.0700	0.0040	-0.0274	0.0102	0.3919	0.1468
**rs7146395**	C	-0.0227	0.0039	0.0079	0.0099	-0.3488	0.4385
**rs7178196**	A	-0.0345	0.0049	-0.0161	0.0116	0.4669	0.3428
**rs7249921**	T	-0.0237	0.0037	-0.0019	0.0097	0.0819	0.4107
**rs73000929**	A	-0.0919	0.0098	-0.0375	0.0447	0.4075	0.4885
**rs73000965**	A	-0.0281	0.0039	0.0231	0.0100	-0.8226	0.3729
**rs73109811**	T	0.0407	0.0047	-0.0149	0.0146	-0.3647	0.3615
**rs75080135**	C	0.1019	0.0048	-0.0094	0.0112	-0.0921	0.1097
**rs75107793**	A	0.1157	0.0071	-0.0411	0.0256	-0.3554	0.2220
**rs75501914**	A	0.0494	0.0074	0.0174	0.0182	0.3526	0.3714
**rs7585866**	G	0.0234	0.0038	-0.0245	0.0141	-1.0496	0.6269
**rs75967349**	G	-0.0554	0.0085	-0.0099	0.0283	0.1789	0.5115
**rs7641175**	A	0.0458	0.0044	0.0050	0.0113	0.1099	0.2466
**rs7665147**	A	-0.0317	0.0047	0.0580	0.0111	-1.8281	0.4436
**rs7696658**	T	0.0215	0.0037	0.0044	0.0099	0.2043	0.4621

**rs77300440**	T	0.0686	0.0067	0.0083	0.0171	0.1205	0.2489
**rs77320796**	G	0.0268	0.0042	0.0055	0.0112	0.2035	0.4199
**rs7776054**	G	0.1192	0.0042	-0.0251	0.0110	-0.2107	0.0924
**rs7788849**	A	-0.0443	0.0062	-0.0147	0.0156	0.3330	0.3558
**rs7811142**	T	0.0358	0.0046	-0.0164	0.0129	-0.4573	0.3646
**rs78265569**	A	-0.0458	0.0065	0.0354	0.0192	-0.7739	0.4344
**rs7833924**	G	0.0439	0.0037	-0.0043	0.0100	-0.0975	0.2291
**rs78565404**	T	0.1420	0.0085	-0.0058	0.0232	-0.0408	0.1634
**rs78909033**	A	0.0653	0.0053	-0.0085	0.0181	-0.1304	0.2773
**rs79287178**	A	0.0642	0.0108	-0.0338	0.0359	-0.5260	0.5658
**rs7950696**	C	0.0274	0.0037	-0.0013	0.0094	-0.0472	0.3432
**rs79936776**	G	0.0787	0.0102	0.0060	0.0282	0.0757	0.3578
**rs80012730**	C	-0.0438	0.0070	0.0103	0.0184	-0.2359	0.4209
**rs8037137**	C	-0.0354	0.0054	0.0233	0.0127	-0.6570	0.3733
**rs8073060**	A	0.0300	0.0040	0.0061	0.0100	0.2040	0.3366
**rs8137128**	T	-0.0418	0.0037	-0.0033	0.0094	0.0790	0.2237
**rs8176747**	G	-0.0546	0.0075	-0.0160	0.0136	0.2934	0.2529
**rs853195**	A	-0.0240	0.0038	-0.0170	0.0100	0.7099	0.4312
**rs926326**	G	-0.0475	0.0043	0.0069	0.0117	-0.1455	0.2466
**rs928391**	C	-0.0260	0.0041	0.0186	0.0107	-0.7146	0.4246
**rs9402633**	T	0.0328	0.0045	0.0018	0.0116	0.0563	0.3535
**rs9462031**	T	-0.0432	0.0051	0.0535	0.0139	-1.2401	0.3534
**rs9524862**	A	0.0351	0.0037	-0.0047	0.0093	-0.1349	0.2654
**rs9809116**	G	0.0265	0.0037	0.0188	0.0095	0.7106	0.3726
**rs9810259**	G	-0.0409	0.0037	-0.0156	0.0095	0.3800	0.2350
**rs9908158**	C	0.0312	0.0038	-0.0042	0.0095	-0.1354	0.3042
**rs9974653**	C	-0.0295	0.0039	0.0225	0.0104	-0.7623	0.3657

EA: effect allele; GX: SNP-platelet count association; GY: SNP-coronary artery disease association; MR: Mendelian randomization; SE: standard error; SNP: single nucleotide polymorphism.

Supplementary Table IV: Inverse variance weighted MR estimates for platelet count and myocardial infarction (Astle et al, 2016; Nikpay et al, 2015)							
**SNP**	**EA**	**GX**	**GX SE**	**GY**	**GY SE**	**MR**	**MR SE**
**rs10048745**	A	0.0293	0.0043	-0.0064	0.0126	-0.2189	0.4304
**rs10058074**	A	-0.0317	0.0036	-0.0019	0.0114	0.0606	0.3590
**rs10075570**	A	-0.0278	0.0042	-0.0025	0.0111	0.0891	0.3979
**rs10199109**	T	-0.0345	0.0039	-0.0041	0.0116	0.1181	0.3353
**rs10220411**	G	0.0330	0.0042	-0.0192	0.0127	-0.5826	0.3933
**rs1034564**	T	0.0272	0.0040	0.0250	0.0115	0.9204	0.4452
**rs10466905**	A	0.0276	0.0047	-0.0070	0.0156	-0.2553	0.5665
**rs1047891**	A	-0.0342	0.0039	-0.0111	0.0116	0.3237	0.3406
**rs1050316**	T	-0.0254	0.0038	-0.0070	0.0108	0.2755	0.4271
**rs10514301**	T	0.0394	0.0057	0.0547	0.0148	1.3879	0.4250
**rs1059196**	T	-0.0305	0.0043	-0.0038	0.0149	0.1252	0.4883
**rs1060431**	A	0.0636	0.0070	-0.0052	0.0190	-0.0821	0.2981
**rs10761741**	T	0.0769	0.0037	0.0055	0.0103	0.0715	0.1339
**rs10769960**	C	-0.0331	0.0037	-0.0100	0.0132	0.3024	0.4006
**rs10811664**	A	-0.0587	0.0050	0.0158	0.0142	-0.2683	0.2436
**rs10820606**	C	0.0499	0.0044	-0.0139	0.0147	-0.2781	0.2962
**rs10893909**	T	-0.0317	0.0042	-0.0142	0.0126	0.4466	0.4003
**rs10940072**	A	-0.0267	0.0037	-0.0267	0.0104	1.0000	0.4151
**rs10974808**	G	0.1218	0.0052	-0.0292	0.0180	-0.2399	0.1479
**rs10984466**	G	0.0378	0.0038	0.0110	0.0113	0.2913	0.2994
**rs11071720**	C	0.0496	0.0040	-0.0079	0.0111	-0.1597	0.2239
**rs11082304**	T	-0.0507	0.0036	0.0098	0.0106	-0.1940	0.2094
**rs11083766**	C	-0.0442	0.0039	0.0243	0.0147	-0.5499	0.3360
**rs11121845**	T	-0.0391	0.0037	-0.0067	0.0105	0.1709	0.2692
**rs11142444**	G	-0.0239	0.0037	0.0095	0.0105	-0.3986	0.4415
**rs11175492**	G	0.0579	0.0061	0.0183	0.0181	0.3162	0.3152
**rs111941366**	T	-0.0432	0.0037	-0.0266	0.0127	0.6157	0.2980
**rs11217191**	A	0.0302	0.0046	0.0014	0.0126	0.0453	0.4156
**rs11240408**	T	-0.0465	0.0038	0.0073	0.0116	-0.1574	0.2496
**rs112790992**	C	0.0304	0.0039	-0.0016	0.0114	-0.0530	0.3755
**rs113128512**	C	-0.0384	0.0063	-0.0023	0.0183	0.0596	0.4770
**rs114694170**	C	0.1632	0.0079	-0.0362	0.0280	-0.2218	0.1718
**rs115487693**	C	-0.0831	0.0088	-0.0137	0.0371	0.1648	0.4472
**rs11553699**	G	-0.0801	0.0056	-0.0324	0.0213	0.4039	0.2675
**rs1155577**	T	0.0217	0.0036	0.0340	0.0102	1.5653	0.5383
**rs11556924**	T	0.0264	0.0037	-0.0689	0.0126	-2.6146	0.6033
**rs11559982**	G	0.0571	0.0037	0.0232	0.0108	0.4070	0.1909
**rs11562010**	A	0.0264	0.0037	-0.0101	0.0105	-0.3814	0.3996
**rs1158570**	C	0.0229	0.0036	0.0030	0.0105	0.1303	0.4576

**rs11604127**	T	0.0931	0.0043	0.0064	0.0156	0.0688	0.1681
**rs116052829**	T	0.0376	0.0060	0.0225	0.0194	0.5995	0.5249
**rs11653357**	A	0.0640	0.0048	0.0166	0.0130	0.2587	0.2040
**rs11734099**	A	0.0574	0.0048	0.0258	0.0139	0.4496	0.2454
**rs1182180**	T	0.0292	0.0037	-0.0074	0.0105	-0.2516	0.3599
**rs11841319**	T	-0.0659	0.0061	-0.0105	0.0168	0.1596	0.2556
**rs1190545**	C	0.0359	0.0042	0.0077	0.0111	0.2152	0.3090
**rs11993146**	A	-0.0266	0.0043	0.0209	0.0115	-0.7857	0.4513
**rs11995702**	G	-0.0232	0.0039	0.0244	0.0112	-1.0484	0.5141
**rs12005199**	A	0.1126	0.0041	0.0072	0.0126	0.0640	0.1119
**rs12052715**	G	-0.0344	0.0041	-0.0032	0.0111	0.0926	0.3241
**rs12096438**	T	-0.0224	0.0036	-0.0030	0.0104	0.1348	0.4665
**rs12458093**	G	0.0229	0.0038	-0.0006	0.0120	-0.0258	0.5257
**rs12459847**	C	-0.0516	0.0042	0.0309	0.0121	-0.5981	0.2402
**rs12491785**	T	-0.0294	0.0037	0.0205	0.0105	-0.6972	0.3685
**rs1260326**	C	-0.0386	0.0037	0.0011	0.0107	-0.0289	0.2763
**rs12608697**	A	0.0305	0.0037	-0.0035	0.0104	-0.1142	0.3395
**rs12976598**	A	0.0512	0.0075	0.0026	0.0231	0.0499	0.4509
**rs1331308**	C	0.0330	0.0037	-0.0158	0.0105	-0.4800	0.3217
**rs1354034**	C	0.1379	0.0037	-0.0012	0.0107	-0.0090	0.0775
**rs141759085**	G	0.0480	0.0067	0.0261	0.0197	0.5434	0.4174
**rs148440689**	A	0.0978	0.0119	0.0514	0.0331	0.5253	0.3446
**rs149290349**	A	-0.0827	0.0071	0.0620	0.0229	-0.7497	0.2839
**rs1506636**	G	0.0290	0.0039	0.0068	0.0118	0.2356	0.4069
**rs151233**	T	0.0648	0.0054	0.0085	0.0207	0.1311	0.3191
**rs1538970**	A	-0.0394	0.0044	0.0138	0.0127	-0.3500	0.3241
**rs1555405**	A	-0.0505	0.0042	-0.0069	0.0114	0.1364	0.2272
**rs1631677**	G	0.0474	0.0052	0.0001	0.0139	0.0013	0.2939
**rs16977972**	T	0.0449	0.0055	-0.0017	0.0155	-0.0374	0.3446
**rs16979901**	G	0.0421	0.0060	0.0186	0.0198	0.4406	0.4753
**rs17145750**	T	-0.0294	0.0050	-0.0001	0.0156	0.0040	0.5328
**rs1716505**	G	0.0440	0.0040	-0.0132	0.0115	-0.2988	0.2620
**rs174548**	G	0.0386	0.0039	-0.0247	0.0116	-0.6385	0.3073
**rs17572109**	A	0.0388	0.0044	0.0187	0.0146	0.4823	0.3811
**rs17580**	A	0.0500	0.0085	-0.0558	0.0296	-1.1158	0.6221
**rs17708984**	A	0.0328	0.0041	-0.0042	0.0111	-0.1284	0.3387
**rs17825630**	A	0.0500	0.0055	-0.0056	0.0152	-0.1114	0.3038
**rs1799945**	G	-0.0351	0.0051	0.0040	0.0155	-0.1129	0.4433
**rs1887430**	A	0.0703	0.0037	-0.0136	0.0105	-0.1933	0.1500
**rs2015599**	A	-0.0445	0.0036	0.0014	0.0102	-0.0316	0.2294
**rs2068888**	A	-0.0237	0.0037	-0.0433	0.0109	1.8257	0.5373
**rs2070667**	A	-0.0550	0.0085	-0.0188	0.0215	0.3412	0.3950
**rs2075672**	G	0.0277	0.0038	-0.0034	0.0111	-0.1217	0.3996

**rs2078064**	A	0.0380	0.0058	0.0168	0.0141	0.4414	0.3782
**rs210142**	C	0.1020	0.0040	-0.0150	0.0124	-0.1473	0.1218
**rs214053**	C	-0.0479	0.0037	-0.0139	0.0102	0.2898	0.2137
**rs216191**	T	0.0370	0.0038	-0.0408	0.0106	-1.1007	0.3067
**rs2235989**	T	0.0280	0.0037	-0.0015	0.0104	-0.0527	0.3724
**rs2255531**	A	-0.0242	0.0038	0.0444	0.0105	-1.8383	0.5242
**rs2283847**	T	-0.0255	0.0038	0.0024	0.0117	-0.0942	0.4582
**rs2284344**	C	-0.0215	0.0037	0.0090	0.0107	-0.4186	0.5024
**rs2331174**	A	-0.0363	0.0037	-0.0110	0.0109	0.3037	0.3014
**rs2448490**	A	-0.0286	0.0038	0.0020	0.0116	-0.0702	0.4053
**rs2518683**	G	-0.0330	0.0052	0.0245	0.0152	-0.7425	0.4745
**rs2700937**	T	0.0216	0.0037	0.0161	0.0104	0.7458	0.4967
**rs2736100**	A	-0.0336	0.0036	0.0278	0.0110	-0.8278	0.3388
**rs2810491**	C	0.0399	0.0042	0.0108	0.0115	0.2716	0.2895
**rs2836441**	A	-0.0435	0.0052	0.0076	0.0159	-0.1742	0.3657
**rs28550009**	G	0.0737	0.0057	0.0016	0.0182	0.0218	0.2462
**rs2862064**	G	-0.0297	0.0048	0.0185	0.0152	-0.6220	0.5210
**rs2894802**	G	-0.0280	0.0037	-0.0052	0.0104	0.1867	0.3731
**rs290268**	G	0.0220	0.0036	-0.0093	0.0102	-0.4221	0.4712
**rs2932536**	A	-0.0346	0.0036	0.0056	0.0102	-0.1627	0.2970
**rs2958137**	G	-0.0356	0.0037	-0.0062	0.0107	0.1745	0.3002
**rs3184504**	C	-0.1039	0.0036	-0.0733	0.0117	0.7060	0.1149
**rs34038797**	G	-0.0297	0.0038	-0.0123	0.0104	0.4142	0.3556
**rs342292**	G	-0.0716	0.0037	-0.0077	0.0109	0.1072	0.1523
**rs34536443**	C	-0.0614	0.0086	-0.0579	0.0403	0.9436	0.6705
**rs34623301**	A	0.0663	0.0044	0.0120	0.0139	0.1805	0.2097
**rs34667100**	C	-0.0460	0.0036	-0.0030	0.0131	0.0656	0.2861
**rs34950321**	T	-0.0876	0.0137	0.0186	0.0400	-0.2124	0.4577
**rs35427**	G	-0.0256	0.0039	-0.0005	0.0112	0.0199	0.4399
**rs35430985**	A	-0.0254	0.0041	0.0102	0.0115	-0.4017	0.4571
**rs36109901**	C	0.0565	0.0041	0.0237	0.0112	0.4185	0.2000
**rs3731211**	A	0.0411	0.0041	-0.0093	0.0119	-0.2253	0.2918
**rs3741404**	C	-0.0224	0.0038	-0.0102	0.0115	0.4568	0.5181
**rs3747207**	A	-0.0345	0.0045	-0.0196	0.0123	0.5664	0.3629
**rs3778028**	A	0.1172	0.0196	-0.0031	0.0389	-0.0267	0.3318
**rs3804749**	T	-0.0413	0.0037	-0.0001	0.0105	0.0036	0.2547
**rs3809114**	A	-0.0246	0.0037	0.0039	0.0110	-0.1587	0.4474
**rs3809272**	A	-0.0945	0.0040	-0.0396	0.0125	0.4187	0.1339
**rs3819299**	G	0.0936	0.0079	0.0458	0.0210	0.4893	0.2282
**rs3844535**	G	0.0237	0.0040	0.0123	0.0115	0.5194	0.4935
**rs3865444**	A	-0.0225	0.0039	0.0050	0.0117	-0.2223	0.5218
**rs409950**	A	0.0646	0.0049	-0.0105	0.0148	-0.1623	0.2299
**rs41315846**	C	0.0575	0.0037	-0.0045	0.0109	-0.0788	0.1889

**rs41316003**	A	0.1654	0.0276	0.1048	0.0846	0.6335	0.5226
**rs4148435**	A	0.0747	0.0066	-0.0010	0.0162	-0.0131	0.2173
**rs4272720**	G	-0.0350	0.0043	-0.0140	0.0136	0.3987	0.3906
**rs429358**	C	-0.0302	0.0051	0.0962	0.0169	-3.1854	0.7775
**rs4388979**	T	-0.0435	0.0037	0.0017	0.0107	-0.0390	0.2458
**rs4411786**	C	-0.0459	0.0041	0.0250	0.0111	-0.5447	0.2465
**rs4432538**	A	-0.0242	0.0037	0.0038	0.0103	-0.1592	0.4256
**rs4470077**	G	0.0293	0.0047	0.0045	0.0135	0.1521	0.4602
**rs4631704**	T	0.0238	0.0038	-0.0245	0.0108	-1.0279	0.4804
**rs4670779**	T	-0.0244	0.0040	-0.0181	0.0114	0.7411	0.4806
**rs4699154**	C	0.0269	0.0040	-0.0206	0.0113	-0.7657	0.4359
**rs4709819**	A	0.0317	0.0037	-0.0152	0.0101	-0.4786	0.3249
**rs4711890**	G	0.0305	0.0041	-0.0139	0.0122	-0.4543	0.4044
**rs4783186**	C	-0.0422	0.0056	-0.0004	0.0169	0.0104	0.4018
**rs4846217**	T	-0.0361	0.0055	0.0097	0.0149	-0.2691	0.4155
**rs4907622**	C	-0.0304	0.0037	-0.0006	0.0117	0.0195	0.3859
**rs4925750**	C	0.0327	0.0039	0.0281	0.0111	0.8588	0.3555
**rs4937127**	G	-0.0320	0.0036	-0.0047	0.0105	0.1475	0.3302
**rs4937333**	C	-0.0322	0.0037	-0.0019	0.0102	0.0591	0.3177
**rs4965426**	A	-0.0365	0.0052	-0.0079	0.0149	0.2152	0.4096
**rs55707100**	T	0.1143	0.0114	0.0195	0.0337	0.1705	0.2952
**rs56036086**	A	-0.0511	0.0053	0.0048	0.0155	-0.0940	0.3033
**rs56043070**	A	-0.1388	0.0070	-0.0207	0.0254	0.1489	0.1833
**rs56125409**	G	-0.0506	0.0056	-0.0106	0.0155	0.2084	0.3061
**rs57274573**	T	0.0280	0.0049	0.0069	0.0218	0.2447	0.7787
**rs58530613**	C	0.0346	0.0058	0.0127	0.0171	0.3674	0.4968
**rs59739601**	G	-0.0551	0.0069	-0.0169	0.0193	0.3068	0.3517
**rs59865663**	A	0.0408	0.0046	0.0001	0.0162	0.0037	0.3961
**rs6060983**	C	-0.0334	0.0040	-0.0041	0.0121	0.1227	0.3639
**rs619460**	A	-0.0237	0.0037	-0.0090	0.0106	0.3791	0.4528
**rs6425521**	A	-0.0652	0.0046	-0.0049	0.0129	0.0745	0.1984
**rs6445967**	C	-0.0305	0.0039	-0.0152	0.0107	0.4984	0.3559
**rs655029**	A	0.0735	0.0041	0.0009	0.0127	0.0126	0.1732
**rs655641**	G	0.0266	0.0045	0.0072	0.0123	0.2688	0.4645
**rs6556471**	C	-0.0462	0.0039	-0.0010	0.0110	0.0218	0.2372
**rs670179**	A	0.0282	0.0037	-0.0151	0.0107	-0.5351	0.3856
**rs6756513**	A	-0.0251	0.0041	0.0079	0.0116	-0.3149	0.4664
**rs6925716**	C	0.0300	0.0037	0.0016	0.0104	0.0535	0.3449
**rs6961069**	T	0.0261	0.0038	0.0157	0.0105	0.6006	0.4120
**rs6993770**	T	-0.0700	0.0040	-0.0326	0.0113	0.4654	0.1632
**rs7146395**	C	-0.0227	0.0039	0.0044	0.0110	-0.1941	0.4833
**rs7178196**	A	-0.0345	0.0049	-0.0091	0.0128	0.2641	0.3741
**rs7249921**	T	-0.0237	0.0037	-0.0019	0.0107	0.0820	0.4522

**rs73000929**	A	-0.0919	0.0098	-0.0542	0.0487	0.5893	0.5334
**rs73000965**	A	-0.0281	0.0039	0.0205	0.0110	-0.7277	0.4049
**rs73109811**	T	0.0407	0.0047	0.0216	0.0164	0.5295	0.4065
**rs75080135**	C	0.1019	0.0048	0.0023	0.0124	0.0229	0.1214
**rs75107793**	A	0.1157	0.0071	-0.0191	0.0282	-0.1651	0.2438
**rs75501914**	A	0.0494	0.0074	0.0232	0.0201	0.4695	0.4136
**rs7585866**	G	0.0234	0.0038	-0.0325	0.0149	-1.3910	0.6765
**rs75967349**	G	-0.0554	0.0085	0.0088	0.0316	-0.1582	0.5708
**rs7641175**	A	0.0458	0.0044	0.0063	0.0125	0.1369	0.2731
**rs7665147**	A	-0.0317	0.0047	0.0442	0.0124	-1.3939	0.4422
**rs7696658**	T	0.0215	0.0037	0.0021	0.0111	0.0959	0.5183
**rs77300440**	T	0.0686	0.0067	0.0051	0.0193	0.0743	0.2816
**rs77320796**	G	0.0268	0.0042	-0.0063	0.0123	-0.2334	0.4592
**rs7776054**	G	0.1192	0.0042	-0.0183	0.0124	-0.1535	0.1040
**rs7788849**	A	-0.0443	0.0062	-0.0224	0.0175	0.5051	0.4005
**rs7811142**	T	0.0358	0.0046	-0.0060	0.0144	-0.1689	0.4046
**rs78265569**	A	-0.0458	0.0065	0.0356	0.0210	-0.7780	0.4710
**rs7833924**	G	0.0439	0.0037	-0.0059	0.0111	-0.1348	0.2541
**rs78565404**	T	0.1420	0.0085	-0.0118	0.0260	-0.0829	0.1831
**rs78909033**	A	0.0653	0.0053	0.0007	0.0199	0.0108	0.3050
**rs79287178**	A	0.0642	0.0108	-0.0534	0.0406	-0.8321	0.6478
**rs7950696**	C	0.0274	0.0037	0.0020	0.0105	0.0736	0.3830
**rs79936776**	G	0.0787	0.0102	0.0144	0.0310	0.1836	0.3944
**rs80012730**	C	-0.0438	0.0070	0.0000	0.0204	0.0008	0.4655
**rs8037137**	C	-0.0354	0.0054	0.0091	0.0144	-0.2573	0.4092
**rs8073060**	A	0.0300	0.0040	0.0123	0.0111	0.4097	0.3757
**rs8137128**	T	-0.0418	0.0037	-0.0026	0.0103	0.0612	0.2465
**rs8176747**	G	-0.0546	0.0075	0.0175	0.0152	-0.3202	0.2819
**rs853195**	A	-0.0240	0.0038	-0.0113	0.0110	0.4721	0.4667
**rs926326**	G	-0.0475	0.0043	-0.0001	0.0129	0.0031	0.2709
**rs928391**	C	-0.0260	0.0041	0.0159	0.0117	-0.6093	0.4605
**rs9402633**	T	0.0328	0.0045	0.0029	0.0129	0.0870	0.3943
**rs9462031**	T	-0.0432	0.0051	0.0559	0.0155	-1.2952	0.3897
**rs9524862**	A	0.0351	0.0037	-0.0087	0.0103	-0.2468	0.2942
**rs9809116**	G	0.0265	0.0037	0.0212	0.0106	0.8013	0.4143
**rs9810259**	G	-0.0409	0.0037	-0.0352	0.0106	0.8607	0.2697
**rs9908158**	C	0.0312	0.0038	0.0107	0.0106	0.3448	0.3418
**rs9974653**	C	-0.0295	0.0039	0.0257	0.0114	-0.8699	0.4046

EA: effect allele; GX: SNP-platelet count association; GY: SNP-myocardial infarction association; MR: Mendelian randomization; SE: standard error; SNP: single nucleotide polymorphism.

Supplementary Table V: Inverse variance weighted MR estimates for platelet count and ischemic stroke (Astle et al, 2016; Malik et al, 2018)							
**SNP**	**EA**	**GX**	**GX SE**	**GY**	**GY SE**	**MR**	**MR SE**
**rs10048745**	A	0.0293	0.0043	-0.0055	0.0108	-0.1874	0.3690
**rs10058074**	A	-0.0317	0.0036	0.0090	0.0095	-0.2839	0.3014
**rs10075570**	A	-0.0278	0.0042	0.0032	0.0090	-0.1150	0.3240
**rs10199109**	T	-0.0345	0.0039	0.0077	0.0094	-0.2231	0.2735
**rs10220411**	G	0.0330	0.0042	0.0065	0.0100	0.1971	0.3043
**rs1034564**	T	0.0272	0.0040	0.0166	0.0094	0.6111	0.3577
**rs10466905**	A	0.0276	0.0047	-0.0012	0.0121	-0.0435	0.4390
**rs1047891**	A	-0.0342	0.0039	-0.0057	0.0098	0.1668	0.2875
**rs1050316**	T	-0.0254	0.0038	-0.0086	0.0092	0.3385	0.3657
**rs10514301**	T	0.0394	0.0057	0.0264	0.0113	0.6694	0.3021
**rs1059196**	T	-0.0305	0.0043	-0.0150	0.0116	0.4918	0.3865
**rs1060431**	A	0.0636	0.0070	-0.0168	0.0141	-0.2640	0.2235
**rs10761741**	T	0.0769	0.0037	0.0060	0.0086	0.0780	0.1118
**rs10769960**	C	-0.0331	0.0037	0.0035	0.0101	-0.1056	0.3051
**rs10811664**	A	-0.0587	0.0050	0.0030	0.0107	-0.0511	0.1823
**rs10820606**	C	0.0499	0.0044	-0.0171	0.0110	-0.3428	0.2225
**rs10893909**	T	-0.0317	0.0042	0.0123	0.0105	-0.3879	0.3351
**rs10940072**	A	-0.0267	0.0037	-0.0002	0.0085	0.0075	0.3179
**rs10974808**	G	0.1218	0.0052	0.0040	0.0140	0.0329	0.1150
**rs10984466**	G	0.0378	0.0038	0.0159	0.0091	0.4209	0.2446
**rs11071720**	C	0.0496	0.0040	-0.0272	0.0091	-0.5486	0.1888
**rs11082304**	T	-0.0507	0.0036	-0.0067	0.0086	0.1322	0.1700
**rs11083766**	C	-0.0442	0.0039	-0.0162	0.0112	0.3666	0.2555
**rs11121845**	T	-0.0391	0.0037	-0.0093	0.0084	0.2378	0.2160
**rs11142444**	G	-0.0239	0.0037	0.0248	0.0087	-1.0368	0.3969
**rs11175492**	G	0.0579	0.0061	0.0085	0.0150	0.1469	0.2596
**rs111941366**	T	-0.0432	0.0037	-0.0307	0.0112	0.7102	0.2660
**rs11217191**	A	0.0302	0.0046	-0.0055	0.0101	-0.1819	0.3352
**rs11240408**	T	-0.0465	0.0038	-0.0045	0.0100	0.0969	0.2154
**rs112790992**	C	0.0304	0.0039	-0.0099	0.0096	-0.3261	0.3190
**rs113128512**	C	-0.0384	0.0063	-0.0226	0.0153	0.5890	0.4103
**rs114694170**	C	0.1632	0.0079	0.0073	0.0227	0.0447	0.1391
**rs115487693**	C	-0.0831	0.0088	0.0171	0.0262	-0.2057	0.3159
**rs11553699**	G	-0.0801	0.0056	0.0219	0.0161	-0.2733	0.2018
**rs1155577**	T	0.0217	0.0036	0.0025	0.0084	0.1152	0.3875
**rs11556924**	T	0.0264	0.0037	-0.0060	0.0106	-0.2276	0.4034
**rs11559982**	G	0.0571	0.0037	-0.0269	0.0092	-0.4714	0.1641
**rs11562010**	A	0.0264	0.0037	-0.0099	0.0090	-0.3744	0.3444
**rs1158570**	C	0.0229	0.0036	-0.0153	0.0085	-0.6683	0.3862
**rs11604127**	T	0.0931	0.0043	-0.0085	0.0130	-0.0913	0.1397

**rs116052829**	T	0.0376	0.0060	0.0154	0.0172	0.4097	0.4622
**rs11653357**	A	0.0640	0.0048	-0.0020	0.0102	-0.0312	0.1593
**rs11734099**	A	0.0574	0.0048	-0.0022	0.0107	-0.0383	0.1863
**rs1182180**	T	0.0292	0.0037	0.0143	0.0086	0.4890	0.3005
**rs11841319**	T	-0.0659	0.0061	-0.0075	0.0140	0.1139	0.2129
**rs1190545**	C	0.0359	0.0042	-0.0073	0.0091	-0.2031	0.2542
**rs11993146**	A	-0.0266	0.0043	0.0078	0.0103	-0.2932	0.3901
**rs11995702**	G	-0.0232	0.0039	-0.0010	0.0095	0.0430	0.4089
**rs12005199**	A	0.1126	0.0041	-0.0038	0.0097	-0.0338	0.0862
**rs12052715**	G	-0.0344	0.0041	-0.0110	0.0096	0.3196	0.2815
**rs12096438**	T	-0.0224	0.0036	-0.0068	0.0082	0.3034	0.3692
**rs12458093**	G	0.0229	0.0038	0.0034	0.0093	0.1488	0.4076
**rs12459847**	C	-0.0516	0.0042	0.0033	0.0111	-0.0640	0.2152
**rs12491785**	T	-0.0294	0.0037	0.0053	0.0086	-0.1803	0.2934
**rs1260326**	C	-0.0386	0.0037	-0.0061	0.0093	0.1580	0.2414
**rs12608697**	A	0.0305	0.0037	0.0000	0.0085	0.0000	0.2783
**rs12976598**	A	0.0512	0.0075	0.0185	0.0178	0.3614	0.3517
**rs1331308**	C	0.0330	0.0037	-0.0029	0.0088	-0.0878	0.2667
**rs1354034**	C	0.1379	0.0037	0.0225	0.0084	0.1632	0.0611
**rs141759085**	G	0.0480	0.0067	0.0228	0.0130	0.4755	0.2792
**rs148440689**	A	0.0978	0.0119	0.0365	0.0352	0.3732	0.3627
**rs149290349**	A	-0.0827	0.0071	-0.0137	0.0207	0.1656	0.2506
**rs1506636**	G	0.0290	0.0039	-0.0093	0.0094	-0.3204	0.3267
**rs151233**	T	0.0648	0.0054	-0.0011	0.0164	-0.0170	0.2530
**rs1538970**	A	-0.0394	0.0044	-0.0095	0.0103	0.2411	0.2627
**rs1555405**	A	-0.0505	0.0042	-0.0229	0.0092	0.4538	0.1862
**rs1631677**	G	0.0474	0.0052	-0.0015	0.0112	-0.0316	0.2363
**rs16977972**	T	0.0449	0.0055	-0.0128	0.0116	-0.2854	0.2610
**rs16979901**	G	0.0421	0.0060	-0.0048	0.0149	-0.1140	0.3542
**rs17145750**	T	-0.0294	0.0050	-0.0167	0.0126	0.5688	0.4398
**rs1716505**	G	0.0440	0.0040	0.0123	0.0094	0.2794	0.2150
**rs174548**	G	0.0386	0.0039	-0.0319	0.0092	-0.8256	0.2524
**rs17572109**	A	0.0388	0.0044	0.0080	0.0116	0.2063	0.3000
**rs17580**	A	0.0500	0.0085	-0.0174	0.0256	-0.3477	0.5149
**rs17708984**	A	0.0328	0.0041	-0.0005	0.0091	-0.0153	0.2777
**rs17825630**	A	0.0500	0.0055	-0.0024	0.0127	-0.0480	0.2542
**rs1799945**	G	-0.0351	0.0051	0.0331	0.0135	-0.9438	0.4085
**rs1887430**	A	0.0703	0.0037	0.0037	0.0084	0.0526	0.1194
**rs2015599**	A	-0.0445	0.0036	-0.0002	0.0085	0.0045	0.1912
**rs2068888**	A	-0.0237	0.0037	-0.0354	0.0086	1.4924	0.4296
**rs2070667**	A	-0.0550	0.0085	-0.0167	0.0150	0.3038	0.2769
**rs2075672**	G	0.0277	0.0038	0.0032	0.0091	0.1154	0.3286
**rs2078064**	A	0.0380	0.0058	0.0019	0.0117	0.0500	0.3079

**rs210142**	C	0.1020	0.0040	0.0001	0.0097	0.0010	0.0951
**rs214053**	C	-0.0479	0.0037	0.0030	0.0085	-0.0626	0.1774
**rs216191**	T	0.0370	0.0038	-0.0111	0.0090	-0.2998	0.2450
**rs2235989**	T	0.0280	0.0037	0.0132	0.0085	0.4711	0.3095
**rs2255531**	A	-0.0242	0.0038	-0.0101	0.0086	0.4177	0.3618
**rs2283847**	T	-0.0255	0.0038	0.0182	0.0086	-0.7151	0.3545
**rs2284344**	C	-0.0215	0.0037	0.0043	0.0083	-0.1998	0.3872
**rs2331174**	A	-0.0363	0.0037	-0.0129	0.0088	0.3555	0.2452
**rs2448490**	A	-0.0286	0.0038	0.0153	0.0090	-0.5349	0.3226
**rs2518683**	G	-0.0330	0.0052	0.0044	0.0124	-0.1335	0.3767
**rs2700937**	T	0.0216	0.0037	-0.0035	0.0085	-0.1622	0.3948
**rs2736100**	A	-0.0336	0.0036	-0.0050	0.0084	0.1490	0.2509
**rs2810491**	C	0.0399	0.0042	-0.0131	0.0092	-0.3284	0.2332
**rs2836441**	A	-0.0435	0.0052	-0.0156	0.0123	0.3586	0.2859
**rs28550009**	G	0.0737	0.0057	0.0153	0.0130	0.2075	0.1771
**rs2862064**	G	-0.0297	0.0048	-0.0083	0.0124	0.2793	0.4198
**rs2894802**	G	-0.0280	0.0037	-0.0008	0.0088	0.0286	0.3147
**rs290268**	G	0.0220	0.0036	0.0065	0.0084	0.2956	0.3851
**rs2932536**	A	-0.0346	0.0036	-0.0012	0.0082	0.0347	0.2373
**rs2958137**	G	-0.0356	0.0037	-0.0050	0.0084	0.1405	0.2365
**rs3184504**	C	-0.1039	0.0036	-0.0751	0.0098	0.7230	0.0977
**rs34038797**	G	-0.0297	0.0038	-0.0019	0.0087	0.0640	0.2930
**rs342292**	G	-0.0716	0.0037	-0.0067	0.0091	0.0936	0.1273
**rs34536443**	C	-0.0614	0.0086	-0.0450	0.0290	0.7335	0.4836
**rs34623301**	A	0.0663	0.0044	0.0086	0.0100	0.1296	0.1510
**rs34667100**	C	-0.0460	0.0036	-0.0134	0.0100	0.2916	0.2188
**rs35427**	G	-0.0256	0.0039	-0.0492	0.0092	1.9251	0.4632
**rs35430985**	A	-0.0254	0.0041	0.0207	0.0095	-0.8139	0.3961
**rs36109901**	C	0.0565	0.0041	0.0093	0.0089	0.1645	0.1579
**rs3731211**	A	0.0411	0.0041	0.0066	0.0095	0.1607	0.2319
**rs3741404**	C	-0.0224	0.0038	-0.0135	0.0089	0.6036	0.4109
**rs3747207**	A	-0.0345	0.0045	-0.0155	0.0097	0.4488	0.2868
**rs3778028**	A	0.1172	0.0196	-0.0147	0.0209	-0.1254	0.1795
**rs3804749**	T	-0.0413	0.0037	-0.0155	0.0085	0.3756	0.2088
**rs3809114**	A	-0.0246	0.0037	-0.0066	0.0085	0.2685	0.3481
**rs3809272**	A	-0.0945	0.0040	-0.0265	0.0104	0.2803	0.1106
**rs3819299**	G	0.0936	0.0079	-0.0066	0.0184	-0.0705	0.1967
**rs3844535**	G	0.0237	0.0040	-0.0075	0.0095	-0.3162	0.4040
**rs3865444**	A	-0.0225	0.0039	-0.0128	0.0096	0.5695	0.4383
**rs409950**	A	0.0646	0.0049	-0.0126	0.0114	-0.1951	0.1772
**rs41315846**	C	0.0575	0.0037	0.0015	0.0085	0.0261	0.1478
**rs4148435**	A	0.0747	0.0066	-0.0088	0.0129	-0.1178	0.1730
**rs4272720**	G	-0.0350	0.0043	-0.0006	0.0105	0.0171	0.2999

**rs429358**	C	-0.0302	0.0051	-0.0081	0.0125	0.2682	0.4164
**rs4388979**	T	-0.0435	0.0037	-0.0033	0.0089	0.0758	0.2046
**rs4411786**	C	-0.0459	0.0041	-0.0101	0.0091	0.2199	0.1991
**rs4432538**	A	-0.0242	0.0037	-0.0128	0.0083	0.5295	0.3526
**rs4470077**	G	0.0293	0.0047	-0.0058	0.0108	-0.1976	0.3694
**rs4631704**	T	0.0238	0.0038	-0.0004	0.0091	-0.0168	0.3820
**rs4670779**	T	-0.0244	0.0040	0.0065	0.0101	-0.2661	0.4157
**rs4699154**	C	0.0269	0.0040	-0.0066	0.0096	-0.2454	0.3587
**rs4709819**	A	0.0317	0.0037	-0.0054	0.0085	-0.1703	0.2688
**rs4711890**	G	0.0305	0.0041	0.0034	0.0100	0.1114	0.3281
**rs4783186**	C	-0.0422	0.0056	0.0114	0.0125	-0.2703	0.2985
**rs4846217**	T	-0.0361	0.0055	0.0018	0.0132	-0.0498	0.3653
**rs4907622**	C	-0.0304	0.0037	0.0221	0.0089	-0.7262	0.3052
**rs4925750**	C	0.0327	0.0039	0.0105	0.0089	0.3210	0.2748
**rs4937127**	G	-0.0320	0.0036	-0.0146	0.0088	0.4567	0.2802
**rs4937333**	C	-0.0322	0.0037	-0.0030	0.0082	0.0930	0.2546
**rs4965426**	A	-0.0365	0.0052	-0.0049	0.0119	0.1342	0.3264
**rs55707100**	T	0.1143	0.0114	0.0487	0.0314	0.4262	0.2781
**rs56036086**	A	-0.0511	0.0053	-0.0024	0.0134	0.0470	0.2622
**rs56043070**	A	-0.1388	0.0070	-0.0045	0.0190	0.0324	0.1369
**rs56125409**	G	-0.0506	0.0056	-0.0149	0.0120	0.2944	0.2393
**rs57274573**	T	0.0280	0.0049	0.0140	0.0134	0.4996	0.4860
**rs58530613**	C	0.0346	0.0058	0.0046	0.0142	0.1329	0.4110
**rs59739601**	G	-0.0551	0.0069	0.0060	0.0133	-0.1089	0.2417
**rs59865663**	A	0.0408	0.0046	-0.0121	0.0108	-0.2965	0.2667
**rs6060983**	C	-0.0334	0.0040	-0.0106	0.0100	0.3178	0.3022
**rs619460**	A	-0.0237	0.0037	-0.0230	0.0090	0.9694	0.4091
**rs6425521**	A	-0.0652	0.0046	-0.0171	0.0101	0.2621	0.1559
**rs6445967**	C	-0.0305	0.0039	-0.0013	0.0087	0.0427	0.2856
**rs655029**	A	0.0735	0.0041	0.0004	0.0112	0.0054	0.1524
**rs655641**	G	0.0266	0.0045	0.0135	0.0104	0.5066	0.3997
**rs6556471**	C	-0.0462	0.0039	0.0138	0.0091	-0.2989	0.1987
**rs670179**	A	0.0282	0.0037	0.0007	0.0090	0.0248	0.3190
**rs6756513**	A	-0.0251	0.0041	-0.0131	0.0091	0.5213	0.3719
**rs6925716**	C	0.0300	0.0037	-0.0138	0.0083	-0.4592	0.2818
**rs6961069**	T	0.0261	0.0038	0.0043	0.0085	0.1650	0.3270
**rs6993770**	T	-0.0700	0.0040	-0.0202	0.0092	0.2886	0.1325
**rs7146395**	C	-0.0227	0.0039	-0.0054	0.0089	0.2376	0.3937
**rs7178196**	A	-0.0345	0.0049	-0.0093	0.0102	0.2697	0.2983
**rs7249921**	T	-0.0237	0.0037	0.0206	0.0092	-0.8691	0.4118
**rs73000965**	A	-0.0281	0.0039	0.0019	0.0093	-0.0675	0.3306
**rs73109811**	T	0.0407	0.0047	0.0296	0.0127	0.7267	0.3230
**rs75080135**	C	0.1019	0.0048	0.0107	0.0108	0.1050	0.1061

**rs75107793**	A	0.1157	0.0071	0.0049	0.0217	0.0423	0.1875
**rs75501914**	A	0.0494	0.0074	0.0200	0.0158	0.4049	0.3256
**rs7585866**	G	0.0234	0.0038	-0.0217	0.0130	-0.9286	0.5767
**rs75967349**	G	-0.0554	0.0085	0.0011	0.0261	-0.0199	0.4712
**rs7641175**	A	0.0458	0.0044	-0.0090	0.0114	-0.1965	0.2496
**rs7665147**	A	-0.0317	0.0047	0.0241	0.0101	-0.7595	0.3377
**rs7696658**	T	0.0215	0.0037	-0.0113	0.0089	-0.5263	0.4241
**rs77300440**	T	0.0686	0.0067	-0.0225	0.0154	-0.3280	0.2268
**rs77320796**	G	0.0268	0.0042	0.0027	0.0110	0.1008	0.4108
**rs7776054**	G	0.1192	0.0042	0.0107	0.0096	0.0898	0.0806
**rs7788849**	A	-0.0443	0.0062	0.0032	0.0148	-0.0723	0.3344
**rs7811142**	T	0.0358	0.0046	0.0009	0.0117	0.0252	0.3272
**rs78265569**	A	-0.0458	0.0065	0.0027	0.0183	-0.0590	0.3998
**rs7833924**	G	0.0439	0.0037	0.0039	0.0093	0.0888	0.2120
**rs78565404**	T	0.1420	0.0085	0.0002	0.0204	0.0014	0.1436
**rs78909033**	A	0.0653	0.0053	0.0010	0.0156	0.0153	0.2389
**rs79287178**	A	0.0642	0.0108	-0.0065	0.0327	-0.1013	0.5098
**rs7950696**	C	0.0274	0.0037	-0.0171	0.0088	-0.6237	0.3316
**rs79936776**	G	0.0787	0.0102	0.0153	0.0247	0.1944	0.3148
**rs80012730**	C	-0.0438	0.0070	-0.0066	0.0190	0.1507	0.4344
**rs8037137**	C	-0.0354	0.0054	-0.0157	0.0110	0.4429	0.3177
**rs8073060**	A	0.0300	0.0040	-0.0105	0.0095	-0.3506	0.3206
**rs8137128**	T	-0.0418	0.0037	-0.0064	0.0088	0.1530	0.2108
**rs8176747**	G	-0.0546	0.0075	0.0045	0.0138	-0.0824	0.2530
**rs853195**	A	-0.0240	0.0038	-0.0157	0.0093	0.6548	0.4014
**rs926326**	G	-0.0475	0.0043	-0.0087	0.0110	0.1832	0.2323
**rs928391**	C	-0.0260	0.0041	0.0027	0.0101	-0.1037	0.3884
**rs9402633**	T	0.0328	0.0045	0.0203	0.0105	0.6193	0.3313
**rs9462031**	T	-0.0432	0.0051	0.0125	0.0132	-0.2896	0.3077
**rs9524862**	A	0.0351	0.0037	0.0038	0.0084	0.1083	0.2397
**rs9809116**	G	0.0265	0.0037	0.0056	0.0086	0.2113	0.3259
**rs9810259**	G	-0.0409	0.0037	-0.0157	0.0085	0.3836	0.2105
**rs9908158**	C	0.0312	0.0038	-0.0102	0.0085	-0.3272	0.2756
**rs9974653**	C	-0.0295	0.0039	0.0145	0.0093	-0.4910	0.3217

EA: effect allele; GX: SNP-platelet count association; GY: SNP-ischemic stroke association; MR: Mendelian randomization; SE: standard error; SNP: single nucleotide polymorphism.

Supplementary Table VI: Inverse variance weighted MR estimates for platelet count and cardioembolic stroke (Astle et al, 2016; Malik et al, 2018)							
**SNP**	**EA**	**GX**	**GX SE**	**GY**	**GY SE**	**MR**	**MR SE**
**rs10048745**	A	0.0293	0.0043	-0.0169	0.0210	-0.5759	0.7204
**rs10058074**	A	-0.0317	0.0036	0.0015	0.0182	-0.0473	0.5741
**rs10075570**	A	-0.0278	0.0042	-0.0031	0.0189	0.1114	0.6797
**rs10199109**	T	-0.0345	0.0039	-0.0063	0.0196	0.1825	0.5682
**rs10220411**	G	0.0330	0.0042	0.0433	0.0202	1.3133	0.6346
**rs1034564**	T	0.0272	0.0040	-0.0022	0.0191	-0.0810	0.7032
**rs10466905**	A	0.0276	0.0047	0.0048	0.0241	0.1741	0.8748
**rs1047891**	A	-0.0342	0.0039	0.0119	0.0206	-0.3483	0.6042
**rs1050316**	T	-0.0254	0.0038	-0.0183	0.0184	0.7202	0.7323
**rs10514301**	T	0.0394	0.0057	0.0430	0.0245	1.0903	0.6405
**rs1059196**	T	-0.0305	0.0043	-0.0697	0.0266	2.2853	0.9293
**rs1060431**	A	0.0636	0.0070	0.0390	0.0301	0.6130	0.4779
**rs10761741**	T	0.0769	0.0037	0.0443	0.0183	0.5758	0.2395
**rs10769960**	C	-0.0331	0.0037	-0.0010	0.0201	0.0302	0.6067
**rs10820606**	C	0.0499	0.0044	-0.0409	0.0226	-0.8199	0.4587
**rs10893909**	T	-0.0317	0.0042	0.0363	0.0212	-1.1447	0.6856
**rs10940072**	A	-0.0267	0.0037	-0.0090	0.0174	0.3366	0.6524
**rs10974808**	G	0.1218	0.0052	-0.0105	0.0278	-0.0862	0.2284
**rs10984466**	G	0.0378	0.0038	0.0279	0.0183	0.7386	0.4901
**rs11071720**	C	0.0496	0.0040	-0.0198	0.0208	-0.3993	0.4207
**rs11082304**	T	-0.0507	0.0036	0.0043	0.0179	-0.0849	0.3533
**rs11083766**	C	-0.0442	0.0039	0.0004	0.0228	-0.0091	0.5160
**rs11121845**	T	-0.0391	0.0037	-0.0135	0.0170	0.3451	0.4359
**rs11142444**	G	-0.0239	0.0037	0.0333	0.0176	-1.3921	0.7661
**rs11175492**	G	0.0579	0.0061	-0.0078	0.0312	-0.1348	0.5393
**rs111941366**	T	-0.0432	0.0037	-0.0550	0.0218	1.2723	0.5158
**rs11217191**	A	0.0302	0.0046	0.0076	0.0210	0.2514	0.6957
**rs11240408**	T	-0.0465	0.0038	-0.0436	0.0185	0.9385	0.4055
**rs112790992**	C	0.0304	0.0039	-0.0393	0.0201	-1.2943	0.6829
**rs113128512**	C	-0.0384	0.0063	-0.0565	0.0318	1.4726	0.8634
**rs114694170**	C	0.1632	0.0079	0.0324	0.0467	0.1986	0.2864
**rs115487693**	C	-0.0831	0.0088	-0.0040	0.0564	0.0481	0.6784
**rs11553699**	G	-0.0801	0.0056	0.0424	0.0311	-0.5291	0.3899
**rs1155577**	T	0.0217	0.0036	0.0101	0.0172	0.4653	0.7962
**rs11556924**	T	0.0264	0.0037	0.0145	0.0214	0.5501	0.8156
**rs11559982**	G	0.0571	0.0037	-0.0330	0.0184	-0.5783	0.3246
**rs11562010**	A	0.0264	0.0037	0.0090	0.0176	0.3404	0.6674
**rs1158570**	C	0.0229	0.0036	-0.0130	0.0174	-0.5678	0.7654
**rs11604127**	T	0.0931	0.0043	0.0206	0.0253	0.2213	0.2720

**rs116052829**	T	0.0376	0.0060	0.0195	0.0346	0.5188	0.9242
**rs11653357**	A	0.0640	0.0048	-0.0275	0.0216	-0.4294	0.3388
**rs11734099**	A	0.0574	0.0048	-0.0065	0.0231	-0.1132	0.4023
**rs1182180**	T	0.0292	0.0037	0.0190	0.0175	0.6498	0.6041
**rs11841319**	T	-0.0659	0.0061	-0.0035	0.0300	0.0531	0.4556
**rs1190545**	C	0.0359	0.0042	-0.0079	0.0191	-0.2198	0.5320
**rs11993146**	A	-0.0266	0.0043	0.0143	0.0207	-0.5376	0.7830
**rs11995702**	G	-0.0232	0.0039	-0.0016	0.0188	0.0689	0.8092
**rs12005199**	A	0.1126	0.0041	-0.0339	0.0201	-0.3011	0.1789
**rs12052715**	G	-0.0344	0.0041	0.0052	0.0192	-0.1511	0.5582
**rs12096438**	T	-0.0224	0.0036	0.0013	0.0170	-0.0580	0.7586
**rs12458093**	G	0.0229	0.0038	0.0238	0.0191	1.0413	0.8534
**rs12459847**	C	-0.0516	0.0042	0.0080	0.0216	-0.1551	0.4189
**rs12491785**	T	-0.0294	0.0037	0.0069	0.0175	-0.2347	0.5960
**rs1260326**	C	-0.0386	0.0037	0.0134	0.0190	-0.3471	0.4933
**rs12608697**	A	0.0305	0.0037	0.0297	0.0174	0.9725	0.5820
**rs12976598**	A	0.0512	0.0075	-0.0036	0.0381	-0.0703	0.7444
**rs1331308**	C	0.0330	0.0037	0.0193	0.0177	0.5846	0.5400
**rs1354034**	C	0.1379	0.0037	0.0315	0.0174	0.2285	0.1264
**rs141759085**	G	0.0480	0.0067	0.0578	0.0282	1.2053	0.6118
**rs148440689**	A	0.0978	0.0119	0.0305	0.0786	0.3118	0.8045
**rs149290349**	A	-0.0827	0.0071	0.0208	0.0399	-0.2515	0.4828
**rs1506636**	G	0.0290	0.0039	-0.0382	0.0189	-1.3160	0.6746
**rs151233**	T	0.0648	0.0054	-0.0476	0.0332	-0.7344	0.5159
**rs1538970**	A	-0.0394	0.0044	0.0130	0.0211	-0.3299	0.5367
**rs1555405**	A	-0.0505	0.0042	-0.0208	0.0187	0.4122	0.3722
**rs1631677**	G	0.0474	0.0052	0.0200	0.0232	0.4219	0.4916
**rs16977972**	T	0.0449	0.0055	-0.0124	0.0248	-0.2765	0.5539
**rs16979901**	G	0.0421	0.0060	0.0242	0.0311	0.5746	0.7430
**rs17145750**	T	-0.0294	0.0050	-0.0281	0.0260	0.9571	0.9002
**rs1716505**	G	0.0440	0.0040	0.0349	0.0194	0.7928	0.4465
**rs174548**	G	0.0386	0.0039	-0.0459	0.0192	-1.1879	0.5113
**rs17572109**	A	0.0388	0.0044	-0.0168	0.0223	-0.4332	0.5771
**rs17580**	A	0.0500	0.0085	-0.0465	0.0519	-0.9291	1.0490
**rs17708984**	A	0.0328	0.0041	0.0140	0.0187	0.4272	0.5731
**rs17825630**	A	0.0500	0.0055	0.0126	0.0258	0.2521	0.5170
**rs1799945**	G	-0.0351	0.0051	0.0457	0.0269	-1.3031	0.7899
**rs1887430**	A	0.0703	0.0037	0.0127	0.0172	0.1805	0.2447
**rs2015599**	A	-0.0445	0.0036	-0.0059	0.0175	0.1327	0.3938
**rs2068888**	A	-0.0237	0.0037	-0.0430	0.0177	1.8128	0.7970
**rs2070667**	A	-0.0550	0.0085	0.0234	0.0342	-0.4257	0.6257
**rs2075672**	G	0.0277	0.0038	-0.0055	0.0184	-0.1984	0.6642
**rs2078064**	A	0.0380	0.0058	0.0290	0.0249	0.7629	0.6654

**rs210142**	C	0.1020	0.0040	0.0307	0.0194	0.3011	0.1906
**rs214053**	C	-0.0479	0.0037	0.0033	0.0176	-0.0688	0.3671
**rs216191**	T	0.0370	0.0038	0.0003	0.0182	0.0081	0.4916
**rs2235989**	T	0.0280	0.0037	0.0154	0.0173	0.5496	0.6215
**rs2255531**	A	-0.0242	0.0038	-0.0086	0.0176	0.3557	0.7301
**rs2283847**	T	-0.0255	0.0038	0.0200	0.0178	-0.7858	0.7092
**rs2284344**	C	-0.0215	0.0037	0.0156	0.0171	-0.7249	0.8042
**rs2331174**	A	-0.0363	0.0037	-0.0229	0.0178	0.6310	0.4946
**rs2448490**	A	-0.0286	0.0038	0.0128	0.0183	-0.4475	0.6426
**rs2518683**	G	-0.0330	0.0052	0.0101	0.0259	-0.3064	0.7871
**rs2700937**	T	0.0216	0.0037	-0.0041	0.0171	-0.1900	0.7930
**rs2736100**	A	-0.0336	0.0036	-0.0392	0.0170	1.1683	0.5223
**rs2810491**	C	0.0399	0.0042	0.0016	0.0192	0.0401	0.4814
**rs2836441**	A	-0.0435	0.0052	-0.0272	0.0261	0.6252	0.6045
**rs28550009**	G	0.0737	0.0057	0.0342	0.0276	0.4639	0.3761
**rs2862064**	G	-0.0297	0.0048	-0.0287	0.0246	0.9659	0.8427
**rs2894802**	G	-0.0280	0.0037	0.0181	0.0179	-0.6473	0.6457
**rs290268**	G	0.0220	0.0036	0.0263	0.0170	1.1961	0.7981
**rs2932536**	A	-0.0346	0.0036	0.0122	0.0169	-0.3530	0.4904
**rs2958137**	G	-0.0356	0.0037	0.0014	0.0172	-0.0393	0.4834
**rs3184504**	C	-0.1039	0.0036	-0.0615	0.0191	0.5921	0.1850
**rs34038797**	G	-0.0297	0.0038	-0.0001	0.0181	0.0034	0.6093
**rs342292**	G	-0.0716	0.0037	0.0170	0.0182	-0.2376	0.2547
**rs34536443**	C	-0.0614	0.0086	-0.0420	0.0588	0.6846	0.9631
**rs34623301**	A	0.0663	0.0044	0.0090	0.0212	0.1357	0.3197
**rs34667100**	C	-0.0460	0.0036	-0.0146	0.0196	0.3177	0.4273
**rs35427**	G	-0.0256	0.0039	-0.0587	0.0188	2.2969	0.8137
**rs35430985**	A	-0.0254	0.0041	0.0224	0.0197	-0.8807	0.7876
**rs36109901**	C	0.0565	0.0041	0.0336	0.0187	0.5944	0.3336
**rs3731211**	A	0.0411	0.0041	0.0377	0.0200	0.9180	0.4954
**rs3741404**	C	-0.0224	0.0038	0.0230	0.0181	-1.0284	0.8279
**rs3747207**	A	-0.0345	0.0045	-0.0075	0.0202	0.2172	0.5856
**rs3778028**	A	0.1172	0.0196	-0.0438	0.0586	-0.3736	0.5037
**rs3804749**	T	-0.0413	0.0037	0.0050	0.0178	-0.1212	0.4315
**rs3809114**	A	-0.0246	0.0037	-0.0125	0.0174	0.5085	0.7119
**rs3809272**	A	-0.0945	0.0040	-0.0024	0.0212	0.0254	0.2243
**rs3819299**	G	0.0936	0.0079	0.0012	0.0397	0.0128	0.4241
**rs3844535**	G	0.0237	0.0040	-0.0236	0.0196	-0.9950	0.8431
**rs3865444**	A	-0.0225	0.0039	0.0096	0.0194	-0.4271	0.8663
**rs409950**	A	0.0646	0.0049	0.0252	0.0235	0.3902	0.3651
**rs41315846**	C	0.0575	0.0037	0.0198	0.0180	0.3443	0.3138
**rs4148435**	A	0.0747	0.0066	0.0143	0.0287	0.1914	0.3846
**rs4272720**	G	-0.0350	0.0043	-0.0049	0.0211	0.1399	0.6029

**rs429358**	C	-0.0302	0.0051	-0.0280	0.0261	0.9272	0.8784
**rs4388979**	T	-0.0435	0.0037	-0.0338	0.0178	0.7765	0.4142
**rs4411786**	C	-0.0459	0.0041	-0.0015	0.0189	0.0327	0.4115
**rs4432538**	A	-0.0242	0.0037	-0.0306	0.0171	1.2658	0.7328
**rs4470077**	G	0.0293	0.0047	0.0000	0.0220	0.0000	0.7497
**rs4631704**	T	0.0238	0.0038	-0.0259	0.0183	-1.0871	0.7871
**rs4670779**	T	-0.0244	0.0040	0.0070	0.0208	-0.2866	0.8528
**rs4699154**	C	0.0269	0.0040	-0.0132	0.0192	-0.4907	0.7175
**rs4709819**	A	0.0317	0.0037	-0.0059	0.0175	-0.1860	0.5522
**rs4711890**	G	0.0305	0.0041	0.0161	0.0203	0.5277	0.6692
**rs4783186**	C	-0.0422	0.0056	0.0085	0.0272	-0.2016	0.6455
**rs4846217**	T	-0.0361	0.0055	-0.0220	0.0274	0.6087	0.7638
**rs4907622**	C	-0.0304	0.0037	0.0372	0.0178	-1.2224	0.6030
**rs4925750**	C	0.0327	0.0039	-0.0007	0.0189	-0.0214	0.5779
**rs4937127**	G	-0.0320	0.0036	-0.0275	0.0176	0.8603	0.5593
**rs4937333**	C	-0.0322	0.0037	-0.0202	0.0170	0.6265	0.5322
**rs4965426**	A	-0.0365	0.0052	-0.0152	0.0257	0.4162	0.7063
**rs55707100**	T	0.1143	0.0114	-0.0262	0.0680	-0.2293	0.5955
**rs56036086**	A	-0.0511	0.0053	0.0073	0.0265	-0.1428	0.5187
**rs56043070**	A	-0.1388	0.0070	-0.0003	0.0414	0.0022	0.2983
**rs56125409**	G	-0.0506	0.0056	-0.0323	0.0263	0.6381	0.5244
**rs57274573**	T	0.0280	0.0049	-0.0168	0.0286	-0.5995	1.0259
**rs58530613**	C	0.0346	0.0058	0.0212	0.0283	0.6127	0.8244
**rs59739601**	G	-0.0551	0.0069	-0.0083	0.0308	0.1506	0.5592
**rs59865663**	A	0.0408	0.0046	0.0014	0.0229	0.0343	0.5611
**rs6060983**	C	-0.0334	0.0040	0.0151	0.0196	-0.4527	0.5901
**rs619460**	A	-0.0237	0.0037	-0.0334	0.0178	1.4077	0.7825
**rs6425521**	A	-0.0652	0.0046	-0.0011	0.0210	0.0169	0.3219
**rs6445967**	C	-0.0305	0.0039	-0.0118	0.0177	0.3874	0.5831
**rs655029**	A	0.0735	0.0041	0.0337	0.0220	0.4586	0.3004
**rs655641**	G	0.0266	0.0045	-0.0130	0.0222	-0.4878	0.8372
**rs6556471**	C	-0.0462	0.0039	0.0292	0.0190	-0.6324	0.4150
**rs670179**	A	0.0282	0.0037	-0.0060	0.0184	-0.2127	0.6528
**rs6756513**	A	-0.0251	0.0041	-0.0271	0.0190	1.0784	0.7760
**rs6925716**	C	0.0300	0.0037	-0.0059	0.0171	-0.1963	0.5696
**rs6961069**	T	0.0261	0.0038	0.0244	0.0173	0.9361	0.6773
**rs6993770**	T	-0.0700	0.0040	0.0063	0.0189	-0.0900	0.2701
**rs7146395**	C	-0.0227	0.0039	-0.0113	0.0181	0.4973	0.8010
**rs7178196**	A	-0.0345	0.0049	-0.0041	0.0217	0.1189	0.6295
**rs7249921**	T	-0.0237	0.0037	0.0470	0.0201	-1.9830	0.9042
**rs73000929**	A	-0.0919	0.0098	0.0209	0.0709	-0.2274	0.7717
**rs73000965**	A	-0.0281	0.0039	-0.0018	0.0189	0.0640	0.6717
**rs73109811**	T	0.0407	0.0047	0.0112	0.0252	0.2750	0.6195

**rs75080135**	C	0.1019	0.0048	0.0145	0.0211	0.1423	0.2071
**rs75107793**	A	0.1157	0.0071	0.0994	0.0422	0.8589	0.3684
**rs75501914**	A	0.0494	0.0074	-0.0249	0.0354	-0.5041	0.7207
**rs7585866**	G	0.0234	0.0038	-0.0376	0.0251	-1.6090	1.1059
**rs75967349**	G	-0.0554	0.0085	-0.0019	0.0527	0.0343	0.9513
**rs7641175**	A	0.0458	0.0044	-0.0256	0.0220	-0.5589	0.4833
**rs7665147**	A	-0.0317	0.0047	0.0290	0.0207	-0.9139	0.6663
**rs7696658**	T	0.0215	0.0037	-0.0116	0.0181	-0.5403	0.8480
**rs77300440**	T	0.0686	0.0067	-0.0404	0.0334	-0.5890	0.4903
**rs77320796**	G	0.0268	0.0042	-0.0143	0.0217	-0.5336	0.8140
**rs7776054**	G	0.1192	0.0042	0.0384	0.0202	0.3222	0.1699
**rs7788849**	A	-0.0443	0.0062	-0.0196	0.0302	0.4426	0.6848
**rs7811142**	T	0.0358	0.0046	0.0352	0.0234	0.9844	0.6665
**rs78265569**	A	-0.0458	0.0065	-0.0126	0.0371	0.2752	0.8113
**rs7833924**	G	0.0439	0.0037	-0.0128	0.0180	-0.2916	0.4108
**rs78565404**	T	0.1420	0.0085	-0.0570	0.0442	-0.4014	0.3121
**rs78909033**	A	0.0653	0.0053	-0.0061	0.0321	-0.0934	0.4916
**rs79287178**	A	0.0642	0.0108	-0.0058	0.0683	-0.0904	1.0643
**rs7950696**	C	0.0274	0.0037	-0.0131	0.0177	-0.4778	0.6487
**rs79936776**	G	0.0787	0.0102	0.0951	0.0523	1.2082	0.6827
**rs80012730**	C	-0.0438	0.0070	-0.0585	0.0377	1.3356	0.8868
**rs80054178**	C	0.1248	0.0123	0.2058	0.0863	1.6489	0.7102
**rs8037137**	C	-0.0354	0.0054	0.0146	0.0239	-0.4119	0.6772
**rs8073060**	A	0.0300	0.0040	-0.0273	0.0194	-0.9115	0.6591
**rs8137128**	T	-0.0418	0.0037	-0.0259	0.0174	0.6192	0.4196
**rs8176747**	G	-0.0546	0.0075	0.0241	0.0287	-0.4414	0.5292
**rs853195**	A	-0.0240	0.0038	-0.0084	0.0184	0.3503	0.7694
**rs926326**	G	-0.0475	0.0043	0.0111	0.0220	-0.2338	0.4638
**rs928391**	C	-0.0260	0.0041	-0.0063	0.0207	0.2420	0.7961
**rs9402633**	T	0.0328	0.0045	0.0148	0.0218	0.4515	0.6679
**rs9462031**	T	-0.0432	0.0051	0.0305	0.0271	-0.7066	0.6334
**rs9524862**	A	0.0351	0.0037	-0.0208	0.0173	-0.5928	0.4969
**rs9704108**	C	0.0535	0.0078	-0.0318	0.0556	-0.5942	1.0426
**rs9809116**	G	0.0265	0.0037	0.0272	0.0176	1.0264	0.6797
**rs9810259**	G	-0.0409	0.0037	-0.0361	0.0174	0.8821	0.4325
**rs9908158**	C	0.0312	0.0038	-0.0345	0.0175	-1.1066	0.5775
**rs9974653**	C	-0.0295	0.0039	0.0149	0.0190	-0.5046	0.6469

EA: effect allele; GX: SNP-platelet count association; GY: SNP-cardioembolic stroke association; MR: Mendelian randomization; SE: standard error; SNP: single nucleotide polymorphism.

Supplementary Table VII: Inverse variance weighted MR estimates for platelet count and large-artery atherosclerotic stroke (Astle et al, 2016; Malik et al, 2018)							
**SNP**	**EA**	**GX**	**GX SE**	**GY**	**GY SE**	**MR**	**MR SE**
**rs10048745**	A	0.0293	0.0043	0.0105	0.0250	0.3578	0.8534
**rs10058074**	A	-0.0317	0.0036	0.0110	0.0237	-0.3470	0.7486
**rs10075570**	A	-0.0278	0.0042	0.0259	0.0214	-0.9311	0.7821
**rs10199109**	T	-0.0345	0.0039	-0.0160	0.0224	0.4635	0.6511
**rs10220411**	G	0.0330	0.0042	-0.0241	0.0246	-0.7309	0.7518
**rs1034564**	T	0.0272	0.0040	0.0186	0.0227	0.6847	0.8417
**rs10466905**	A	0.0276	0.0047	-0.0381	0.0306	-1.3822	1.1343
**rs1047891**	A	-0.0342	0.0039	-0.0313	0.0245	0.9161	0.7247
**rs1050316**	T	-0.0254	0.0038	-0.0077	0.0216	0.3030	0.8513
**rs10514301**	T	0.0394	0.0057	0.0299	0.0265	0.7581	0.6806
**rs1059196**	T	-0.0305	0.0043	-0.0014	0.0276	0.0459	0.9049
**rs1060431**	A	0.0636	0.0070	-0.0178	0.0346	-0.2798	0.5447
**rs10761741**	T	0.0769	0.0037	0.0253	0.0205	0.3288	0.2669
**rs10769960**	C	-0.0331	0.0037	0.0221	0.0239	-0.6671	0.7253
**rs10811664**	A	-0.0587	0.0050	0.0208	0.0252	-0.3543	0.4303
**rs10820606**	C	0.0499	0.0044	-0.0164	0.0259	-0.3287	0.5200
**rs10893909**	T	-0.0317	0.0042	0.0433	0.0257	-1.3655	0.8305
**rs10940072**	A	-0.0267	0.0037	0.0030	0.0205	-0.1122	0.7668
**rs10974808**	G	0.1218	0.0052	0.0117	0.0347	0.0961	0.2850
**rs10984466**	G	0.0378	0.0038	0.0612	0.0221	1.6201	0.6072
**rs11071720**	C	0.0496	0.0040	-0.0463	0.0219	-0.9338	0.4480
**rs11082304**	T	-0.0507	0.0036	0.0240	0.0208	-0.4736	0.4119
**rs11083766**	C	-0.0442	0.0039	-0.0358	0.0275	0.8102	0.6264
**rs11121845**	T	-0.0391	0.0037	0.0204	0.0198	-0.5216	0.5087
**rs11142444**	G	-0.0239	0.0037	0.0126	0.0213	-0.5267	0.8941
**rs11175492**	G	0.0579	0.0061	0.0061	0.0386	0.1054	0.6670
**rs111941366**	T	-0.0432	0.0037	-0.0239	0.0282	0.5529	0.6540
**rs11217191**	A	0.0302	0.0046	0.0168	0.0241	0.5557	0.8016
**rs11240408**	T	-0.0465	0.0038	0.0350	0.0230	-0.7534	0.4989
**rs112790992**	C	0.0304	0.0039	0.0187	0.0240	0.6159	0.7945
**rs113128512**	C	-0.0384	0.0063	0.0652	0.0369	-1.6993	1.0014
**rs114694170**	C	0.1632	0.0079	0.0971	0.0592	0.5951	0.3640
**rs115487693**	C	-0.0831	0.0088	0.0906	0.0676	-1.0897	0.8212
**rs11553699**	G	-0.0801	0.0056	-0.0079	0.0412	0.0986	0.5142
**rs1155577**	T	0.0217	0.0036	0.0116	0.0199	0.5344	0.9211
**rs11556924**	T	0.0264	0.0037	-0.0399	0.0277	-1.5137	1.0725
**rs11559982**	G	0.0571	0.0037	-0.0144	0.0213	-0.2524	0.3736
**rs11562010**	A	0.0264	0.0037	-0.0084	0.0206	-0.3177	0.7804
**rs1158570**	C	0.0229	0.0036	-0.0021	0.0204	-0.0917	0.8911

**rs11604127**	T	0.0931	0.0043	-0.0590	0.0329	-0.6339	0.3547
**rs116052829**	T	0.0376	0.0060	0.0322	0.0438	0.8567	1.1732
**rs11653357**	A	0.0640	0.0048	0.0197	0.0237	0.3076	0.3708
**rs11734099**	A	0.0574	0.0048	0.0307	0.0256	0.5346	0.4480
**rs1182180**	T	0.0292	0.0037	0.0195	0.0207	0.6669	0.7129
**rs11841319**	T	-0.0659	0.0061	0.0233	0.0336	-0.3538	0.5113
**rs1190545**	C	0.0359	0.0042	0.0160	0.0216	0.4451	0.6031
**rs11993146**	A	-0.0266	0.0043	0.0008	0.0249	-0.0301	0.9360
**rs11995702**	G	-0.0232	0.0039	0.0082	0.0216	-0.3529	0.9315
**rs12005199**	A	0.1126	0.0041	-0.0134	0.0231	-0.1190	0.2052
**rs12052715**	G	-0.0344	0.0041	-0.0564	0.0234	1.6387	0.7070
**rs12096438**	T	-0.0224	0.0036	0.0084	0.0198	-0.3748	0.8855
**rs12458093**	G	0.0229	0.0038	-0.0076	0.0228	-0.3325	0.9990
**rs12459847**	C	-0.0516	0.0042	0.0364	0.0275	-0.7056	0.5362
**rs12491785**	T	-0.0294	0.0037	0.0300	0.0210	-1.0204	0.7260
**rs1260326**	C	-0.0386	0.0037	-0.0122	0.0220	0.3160	0.5707
**rs12608697**	A	0.0305	0.0037	0.0008	0.0205	0.0262	0.6713
**rs12976598**	A	0.0512	0.0075	0.0164	0.0441	0.3204	0.8628
**rs1331308**	C	0.0330	0.0037	-0.0160	0.0209	-0.4846	0.6353
**rs1354034**	C	0.1379	0.0037	0.0099	0.0202	0.0718	0.1465
**rs141759085**	G	0.0480	0.0067	0.0878	0.0304	1.8309	0.6839
**rs148440689**	A	0.0978	0.0119	0.1107	0.0857	1.1318	0.8869
**rs149290349**	A	-0.0827	0.0071	-0.0715	0.0524	0.8644	0.6378
**rs1506636**	G	0.0290	0.0039	-0.0622	0.0229	-2.1427	0.8396
**rs151233**	T	0.0648	0.0054	-0.0425	0.0420	-0.6557	0.6503
**rs1538970**	A	-0.0394	0.0044	0.0038	0.0239	-0.0964	0.6065
**rs1555405**	A	-0.0505	0.0042	-0.0299	0.0221	0.5925	0.4407
**rs1631677**	G	0.0474	0.0052	0.0122	0.0268	0.2574	0.5661
**rs16977972**	T	0.0449	0.0055	-0.0144	0.0278	-0.3210	0.6210
**rs16979901**	G	0.0421	0.0060	-0.0302	0.0388	-0.7171	0.9269
**rs17145750**	T	-0.0294	0.0050	-0.0088	0.0303	0.2997	1.0333
**rs1716505**	G	0.0440	0.0040	0.0365	0.0226	0.8292	0.5189
**rs174548**	G	0.0386	0.0039	-0.0563	0.0218	-1.4570	0.5832
**rs17572109**	A	0.0388	0.0044	0.0444	0.0281	1.1448	0.7359
**rs17580**	A	0.0500	0.0085	-0.0086	0.0637	-0.1718	1.2731
**rs17708984**	A	0.0328	0.0041	-0.0190	0.0217	-0.5798	0.6660
**rs17825630**	A	0.0500	0.0055	-0.0030	0.0306	-0.0600	0.6123
**rs1799945**	G	-0.0351	0.0051	0.0422	0.0328	-1.2033	0.9514
**rs1887430**	A	0.0703	0.0037	-0.0185	0.0203	-0.2630	0.2889
**rs2015599**	A	-0.0445	0.0036	-0.0033	0.0206	0.0742	0.4634
**rs2068888**	A	-0.0237	0.0037	-0.0362	0.0207	1.5262	0.9039
**rs2070667**	A	-0.0550	0.0085	-0.0193	0.0369	0.3511	0.6736
**rs2075672**	G	0.0277	0.0038	0.0296	0.0224	1.0676	0.8209

**rs2078064**	A	0.0380	0.0058	-0.0124	0.0272	-0.3262	0.7173
**rs210142**	C	0.1020	0.0040	0.0195	0.0227	0.1912	0.2227
**rs214053**	C	-0.0479	0.0037	-0.0164	0.0204	0.3421	0.4263
**rs216191**	T	0.0370	0.0038	0.0161	0.0218	0.4349	0.5905
**rs2235989**	T	0.0280	0.0037	0.0221	0.0203	0.7887	0.7317
**rs2255531**	A	-0.0242	0.0038	-0.0376	0.0204	1.5551	0.8788
**rs2283847**	T	-0.0255	0.0038	0.0214	0.0205	-0.8408	0.8153
**rs2284344**	C	-0.0215	0.0037	0.0487	0.0200	-2.2629	1.0068
**rs2331174**	A	-0.0363	0.0037	0.0071	0.0215	-0.1956	0.5928
**rs2448490**	A	-0.0286	0.0038	0.0552	0.0216	-1.9299	0.7975
**rs2518683**	G	-0.0330	0.0052	-0.0539	0.0327	1.6350	1.0252
**rs2700937**	T	0.0216	0.0037	-0.0105	0.0201	-0.4865	0.9350
**rs2736100**	A	-0.0336	0.0036	-0.0006	0.0201	0.0179	0.5991
**rs2810491**	C	0.0399	0.0042	-0.0066	0.0218	-0.1655	0.5468
**rs2836441**	A	-0.0435	0.0052	-0.0393	0.0288	0.9033	0.6706
**rs28550009**	G	0.0737	0.0057	0.0110	0.0311	0.1492	0.4220
**rs2862064**	G	-0.0297	0.0048	-0.0154	0.0310	0.5183	1.0467
**rs2894802**	G	-0.0280	0.0037	-0.0022	0.0230	0.0787	0.8225
**rs290268**	G	0.0220	0.0036	-0.0214	0.0202	-0.9732	0.9327
**rs2932536**	A	-0.0346	0.0036	-0.0251	0.0199	0.7263	0.5809
**rs2958137**	G	-0.0356	0.0037	0.0090	0.0201	-0.2529	0.5655
**rs3184504**	C	-0.1039	0.0036	-0.1143	0.0248	1.1004	0.2418
**rs34038797**	G	-0.0297	0.0038	-0.0661	0.0213	2.2252	0.7716
**rs342292**	G	-0.0716	0.0037	-0.0175	0.0225	0.2446	0.3147
**rs34536443**	C	-0.0614	0.0086	-0.0852	0.0751	1.3887	1.2393
**rs34623301**	A	0.0663	0.0044	0.0031	0.0238	0.0467	0.3588
**rs34667100**	C	-0.0460	0.0036	-0.0018	0.0231	0.0392	0.5027
**rs35427**	G	-0.0256	0.0039	-0.0386	0.0224	1.5104	0.9058
**rs35430985**	A	-0.0254	0.0041	0.0308	0.0234	-1.2110	0.9408
**rs36109901**	C	0.0565	0.0041	0.0327	0.0215	0.5785	0.3826
**rs3731211**	A	0.0411	0.0041	-0.0099	0.0233	-0.2411	0.5678
**rs3741404**	C	-0.0224	0.0038	-0.0096	0.0213	0.4293	0.9552
**rs3747207**	A	-0.0345	0.0045	-0.0779	0.0231	2.2556	0.7294
**rs3778028**	A	0.1172	0.0196	-0.0540	0.0515	-0.4606	0.4460
**rs3804749**	T	-0.0413	0.0037	-0.0110	0.0208	0.2666	0.5047
**rs3809114**	A	-0.0246	0.0037	-0.0017	0.0205	0.0692	0.8340
**rs3809272**	A	-0.0945	0.0040	-0.0313	0.0264	0.3311	0.2796
**rs3819299**	G	0.0936	0.0079	-0.0501	0.0455	-0.5352	0.4882
**rs3844535**	G	0.0237	0.0040	-0.0398	0.0226	-1.6779	0.9936
**rs3865444**	A	-0.0225	0.0039	-0.0223	0.0232	0.9921	1.0463
**rs409950**	A	0.0646	0.0049	-0.0229	0.0274	-0.3546	0.4252
**rs41315846**	C	0.0575	0.0037	-0.0020	0.0210	-0.0348	0.3651
**rs4148435**	A	0.0747	0.0066	-0.0402	0.0298	-0.5382	0.4018

**rs4272720**	G	-0.0350	0.0043	0.0142	0.0261	-0.4056	0.7471
**rs429358**	C	-0.0302	0.0051	0.0044	0.0308	-0.1457	1.0203
**rs4388979**	T	-0.0435	0.0037	0.0268	0.0214	-0.6157	0.4944
**rs4411786**	C	-0.0459	0.0041	-0.0114	0.0221	0.2482	0.4817
**rs4432538**	A	-0.0242	0.0037	-0.0208	0.0201	0.8604	0.8416
**rs4470077**	G	0.0293	0.0047	-0.0107	0.0266	-0.3646	0.9083
**rs4631704**	T	0.0238	0.0038	-0.0040	0.0217	-0.1679	0.9112
**rs4670779**	T	-0.0244	0.0040	-0.0190	0.0249	0.7778	1.0272
**rs4699154**	C	0.0269	0.0040	0.0045	0.0237	0.1673	0.8815
**rs4709819**	A	0.0317	0.0037	0.0237	0.0206	0.7473	0.6555
**rs4711890**	G	0.0305	0.0041	-0.0045	0.0245	-0.1475	0.8033
**rs4783186**	C	-0.0422	0.0056	0.0441	0.0298	-1.0457	0.7199
**rs4846217**	T	-0.0361	0.0055	0.0073	0.0336	-0.2020	0.9302
**rs4907622**	C	-0.0304	0.0037	-0.0006	0.0211	0.0197	0.6934
**rs4925750**	C	0.0327	0.0039	-0.0026	0.0216	-0.0795	0.6605
**rs4937127**	G	-0.0320	0.0036	-0.0414	0.0215	1.2952	0.6886
**rs4937333**	C	-0.0322	0.0037	-0.0201	0.0200	0.6234	0.6245
**rs4965426**	A	-0.0365	0.0052	0.0140	0.0281	-0.3834	0.7714
**rs55707100**	T	0.1143	0.0114	0.1553	0.0778	1.3591	0.6943
**rs56036086**	A	-0.0511	0.0053	0.0007	0.0335	-0.0137	0.6555
**rs56043070**	A	-0.1388	0.0070	-0.0349	0.0496	0.2515	0.3576
**rs56125409**	G	-0.0506	0.0056	0.0026	0.0283	-0.0514	0.5591
**rs57274573**	T	0.0280	0.0049	0.0578	0.0324	2.0627	1.2103
**rs58530613**	C	0.0346	0.0058	0.0286	0.0350	0.8265	1.0211
**rs59739601**	G	-0.0551	0.0069	-0.1063	0.0320	1.9290	0.6288
**rs59865663**	A	0.0408	0.0046	-0.0183	0.0260	-0.4484	0.6391
**rs6060983**	C	-0.0334	0.0040	-0.0307	0.0255	0.9205	0.7724
**rs619460**	A	-0.0237	0.0037	0.0137	0.0216	-0.5774	0.9149
**rs6425521**	A	-0.0652	0.0046	-0.0285	0.0243	0.4368	0.3737
**rs6445967**	C	-0.0305	0.0039	-0.0098	0.0211	0.3217	0.6939
**rs655029**	A	0.0735	0.0041	0.0013	0.0280	0.0177	0.3810
**rs655641**	G	0.0266	0.0045	0.0061	0.0243	0.2289	0.9127
**rs6556471**	C	-0.0462	0.0039	0.0075	0.0217	-0.1624	0.4702
**rs670179**	A	0.0282	0.0037	0.0026	0.0222	0.0922	0.7869
**rs6756513**	A	-0.0251	0.0041	0.0292	0.0217	-1.1620	0.8838
**rs6925716**	C	0.0300	0.0037	-0.0521	0.0202	-1.7338	0.7045
**rs6961069**	T	0.0261	0.0038	0.0249	0.0208	0.9553	0.8098
**rs6993770**	T	-0.0700	0.0040	-0.0411	0.0220	0.5872	0.3161
**rs7146395**	C	-0.0227	0.0039	-0.0024	0.0214	0.1056	0.9419
**rs7178196**	A	-0.0345	0.0049	0.0187	0.0240	-0.5423	0.7002
**rs7249921**	T	-0.0237	0.0037	0.0420	0.0226	-1.7720	0.9939
**rs73000929**	A	-0.0919	0.0098	-0.0864	0.0937	0.9400	1.0243
**rs73000965**	A	-0.0281	0.0039	0.0077	0.0222	-0.2736	0.7899

**rs73109811**	T	0.0407	0.0047	0.0588	0.0320	1.4437	0.8032
**rs75080135**	C	0.1019	0.0048	0.0037	0.0245	0.0363	0.2404
**rs75107793**	A	0.1157	0.0071	-0.0078	0.0556	-0.0674	0.4804
**rs75501914**	A	0.0494	0.0074	0.0665	0.0371	1.3463	0.7779
**rs7585866**	G	0.0234	0.0038	-0.0003	0.0326	-0.0128	1.3951
**rs75967349**	G	-0.0554	0.0085	-0.0342	0.0675	0.6174	1.2221
**rs7641175**	A	0.0458	0.0044	-0.0559	0.0272	-1.2205	0.6053
**rs7665147**	A	-0.0317	0.0047	-0.0022	0.0233	0.0693	0.7344
**rs7696658**	T	0.0215	0.0037	-0.0216	0.0212	-1.0060	1.0021
**rs77300440**	T	0.0686	0.0067	0.0327	0.0362	0.4767	0.5298
**rs77320796**	G	0.0268	0.0042	-0.0380	0.0278	-1.4181	1.0607
**rs7776054**	G	0.1192	0.0042	-0.0259	0.0234	-0.2173	0.1965
**rs7788849**	A	-0.0443	0.0062	-0.0245	0.0358	0.5533	0.8121
**rs7811142**	T	0.0358	0.0046	0.0164	0.0299	0.4587	0.8383
**rs78265569**	A	-0.0458	0.0065	0.0111	0.0451	-0.2424	0.9857
**rs7833924**	G	0.0439	0.0037	0.0335	0.0214	0.7631	0.4917
**rs78565404**	T	0.1420	0.0085	0.0861	0.0525	0.6063	0.3714
**rs78909033**	A	0.0653	0.0053	-0.0560	0.0413	-0.8575	0.6362
**rs79287178**	A	0.0642	0.0108	0.0350	0.0893	0.5453	1.3944
**rs7950696**	C	0.0274	0.0037	0.0350	0.0208	1.2766	0.7775
**rs79936776**	G	0.0787	0.0102	-0.0543	0.0683	-0.6899	0.8723
**rs80012730**	C	-0.0438	0.0070	0.0423	0.0459	-0.9657	1.0592
**rs80054178**	C	0.1248	0.0123	-0.0521	0.1348	-0.4174	1.0808
**rs8037137**	C	-0.0354	0.0054	0.0007	0.0260	-0.0197	0.7335
**rs8073060**	A	0.0300	0.0040	-0.0229	0.0232	-0.7645	0.7813
**rs8137128**	T	-0.0418	0.0037	-0.0067	0.0206	0.1602	0.4927
**rs8176747**	G	-0.0546	0.0075	0.0390	0.0317	-0.7144	0.5889
**rs853195**	A	-0.0240	0.0038	-0.0435	0.0227	1.8142	0.9890
**rs926326**	G	-0.0475	0.0043	-0.0379	0.0272	0.7982	0.5775
**rs928391**	C	-0.0260	0.0041	0.0182	0.0251	-0.6992	0.9706
**rs9402633**	T	0.0328	0.0045	0.0313	0.0245	0.9549	0.7587
**rs9462031**	T	-0.0432	0.0051	0.0362	0.0346	-0.8386	0.8077
**rs9524862**	A	0.0351	0.0037	-0.0235	0.0201	-0.6697	0.5771
**rs9704108**	C	0.0535	0.0078	-0.1284	0.0789	-2.3994	1.5150
**rs9809116**	G	0.0265	0.0037	0.0202	0.0208	0.7623	0.7922
**rs9810259**	G	-0.0409	0.0037	-0.0367	0.0205	0.8967	0.5074
**rs9908158**	C	0.0312	0.0038	-0.0024	0.0203	-0.0770	0.6512
**rs9974653**	C	-0.0295	0.0039	0.0276	0.0233	-0.9346	0.7988

EA: effect allele; GX: SNP-platelet count association; GY: SNP-large-artery atherosclerotic stroke association; MR: Mendelian randomization; SE: standard error; SNP: single nucleotide polymorphism.

Supplementary Table VIII: Inverse variance weighted MR estimates for platelet count and small vessel stroke (Astle et al, 2016; Malik et al, 2018)							
**SNP**	**EA**	**GX**	**GX SE**	**GY**	**GY SE**	**MR**	**MR SE**
**rs10048745**	A	0.0293	0.0043	0.0118	0.0212	0.4021	0.7247
**rs10058074**	A	-0.0317	0.0036	0.0231	0.0217	-0.7286	0.6896
**rs10075570**	A	-0.0278	0.0042	0.0041	0.0175	-0.1474	0.6295
**rs10199109**	T	-0.0345	0.0039	0.0189	0.0184	-0.5475	0.5367
**rs10220411**	G	0.0330	0.0042	0.0130	0.0212	0.3943	0.6449
**rs1034564**	T	0.0272	0.0040	0.0253	0.0191	0.9313	0.7165
**rs10466905**	A	0.0276	0.0047	-0.0336	0.0277	-1.2189	1.0257
**rs1047891**	A	-0.0342	0.0039	-0.0095	0.0209	0.2781	0.6125
**rs1050316**	T	-0.0254	0.0038	-0.0254	0.0179	0.9997	0.7205
**rs10514301**	T	0.0394	0.0057	0.0412	0.0203	1.0446	0.5360
**rs1059196**	T	-0.0305	0.0043	-0.0109	0.0221	0.3574	0.7263
**rs1060431**	A	0.0636	0.0070	-0.0103	0.0270	-0.1619	0.4247
**rs10761741**	T	0.0769	0.0037	-0.0485	0.0174	-0.6304	0.2282
**rs10769960**	C	-0.0331	0.0037	0.0106	0.0198	-0.3200	0.5988
**rs10811664**	A	-0.0587	0.0050	-0.0189	0.0199	0.3219	0.3401
**rs10820606**	C	0.0499	0.0044	0.0176	0.0207	0.3528	0.4161
**rs10893909**	T	-0.0317	0.0042	0.0022	0.0223	-0.0694	0.7033
**rs10940072**	A	-0.0267	0.0037	-0.0055	0.0170	0.2057	0.6364
**rs10974808**	G	0.1218	0.0052	0.0167	0.0316	0.1372	0.2596
**rs10984466**	G	0.0378	0.0038	0.0271	0.0189	0.7174	0.5055
**rs11071720**	C	0.0496	0.0040	-0.0240	0.0182	-0.4841	0.3691
**rs11082304**	T	-0.0507	0.0036	-0.0129	0.0171	0.2546	0.3380
**rs11083766**	C	-0.0442	0.0039	-0.0116	0.0222	0.2625	0.5030
**rs11121845**	T	-0.0391	0.0037	0.0026	0.0163	-0.0665	0.4168
**rs11142444**	G	-0.0239	0.0037	0.0085	0.0183	-0.3553	0.7670
**rs111941366**	T	-0.0432	0.0037	-0.0178	0.0254	0.4118	0.5886
**rs11217191**	A	0.0302	0.0046	-0.0155	0.0199	-0.5127	0.6628
**rs11240408**	T	-0.0465	0.0038	-0.0089	0.0196	0.1916	0.4222
**rs112790992**	C	0.0304	0.0039	-0.0191	0.0200	-0.6291	0.6637
**rs113128512**	C	-0.0384	0.0063	0.0120	0.0316	-0.3128	0.8252
**rs1155577**	T	0.0217	0.0036	0.0044	0.0165	0.2027	0.7609
**rs11556924**	T	0.0264	0.0037	0.0061	0.0239	0.2314	0.9073
**rs11559982**	G	0.0571	0.0037	-0.0137	0.0179	-0.2401	0.3141
**rs11562010**	A	0.0264	0.0037	-0.0263	0.0172	-0.9947	0.6652
**rs1158570**	C	0.0229	0.0036	-0.0220	0.0172	-0.9609	0.7667
**rs11653357**	A	0.0640	0.0048	0.0066	0.0192	0.1031	0.2999
**rs11734099**	A	0.0574	0.0048	-0.0191	0.0208	-0.3326	0.3633
**rs1182180**	T	0.0292	0.0037	0.0130	0.0173	0.4446	0.5943
**rs11841319**	T	-0.0659	0.0061	-0.0303	0.0272	0.4601	0.4152

**rs1190545**	C	0.0359	0.0042	-0.0112	0.0175	-0.3116	0.4882
**rs11993146**	A	-0.0266	0.0043	0.0200	0.0213	-0.7518	0.8099
**rs11995702**	G	-0.0232	0.0039	-0.0059	0.0200	0.2539	0.8618
**rs12005199**	A	0.1126	0.0041	0.0084	0.0191	0.0746	0.1697
**rs12052715**	G	-0.0344	0.0041	-0.0112	0.0206	0.3254	0.5998
**rs12096438**	T	-0.0224	0.0036	-0.0208	0.0164	0.9281	0.7470
**rs12458093**	G	0.0229	0.0038	0.0170	0.0191	0.7438	0.8448
**rs12459847**	C	-0.0516	0.0042	-0.0299	0.0255	0.5796	0.4965
**rs12491785**	T	-0.0294	0.0037	0.0038	0.0175	-0.1293	0.5955
**rs1260326**	C	-0.0386	0.0037	-0.0208	0.0175	0.5388	0.4563
**rs12608697**	A	0.0305	0.0037	-0.0155	0.0170	-0.5075	0.5601
**rs12976598**	A	0.0512	0.0075	-0.0483	0.0370	-0.9436	0.7358
**rs1331308**	C	0.0330	0.0037	-0.0180	0.0174	-0.5452	0.5305
**rs1354034**	C	0.1379	0.0037	0.0323	0.0167	0.2343	0.1213
**rs141759085**	G	0.0480	0.0067	0.0092	0.0233	0.1919	0.4866
**rs1506636**	G	0.0290	0.0039	0.0171	0.0197	0.5891	0.6832
**rs1538970**	A	-0.0394	0.0044	-0.0452	0.0193	1.1469	0.5063
**rs1555405**	A	-0.0505	0.0042	-0.0344	0.0180	0.6817	0.3612
**rs1631677**	G	0.0474	0.0052	0.0016	0.0220	0.0338	0.4641
**rs16977972**	T	0.0449	0.0055	-0.0394	0.0221	-0.8784	0.5043
**rs17145750**	T	-0.0294	0.0050	-0.0277	0.0252	0.9435	0.8730
**rs1716505**	G	0.0440	0.0040	0.0165	0.0189	0.3748	0.4307
**rs174548**	G	0.0386	0.0039	-0.0291	0.0178	-0.7531	0.4669
**rs17572109**	A	0.0388	0.0044	0.0462	0.0262	1.1912	0.6888
**rs17708984**	A	0.0328	0.0041	-0.0221	0.0179	-0.6744	0.5525
**rs17825630**	A	0.0500	0.0055	0.0326	0.0263	0.6523	0.5311
**rs1799945**	G	-0.0351	0.0051	0.0099	0.0291	-0.2823	0.8308
**rs1887430**	A	0.0703	0.0037	0.0074	0.0171	0.1052	0.2431
**rs2015599**	A	-0.0445	0.0036	-0.0126	0.0168	0.2834	0.3786
**rs2068888**	A	-0.0237	0.0037	-0.0109	0.0172	0.4595	0.7286
**rs2070667**	A	-0.0550	0.0085	-0.0394	0.0274	0.7168	0.5108
**rs2075672**	G	0.0277	0.0038	-0.0045	0.0187	-0.1623	0.6749
**rs2078064**	A	0.0380	0.0058	0.0114	0.0214	0.2999	0.5648
**rs210142**	C	0.1020	0.0040	-0.0262	0.0198	-0.2569	0.1944
**rs214053**	C	-0.0479	0.0037	0.0171	0.0172	-0.3567	0.3598
**rs216191**	T	0.0370	0.0038	-0.0596	0.0181	-1.6098	0.5162
**rs2235989**	T	0.0280	0.0037	0.0199	0.0169	0.7102	0.6102
**rs2255531**	A	-0.0242	0.0038	0.0257	0.0167	-1.0629	0.7108
**rs2283847**	T	-0.0255	0.0038	0.0157	0.0169	-0.6169	0.6704
**rs2284344**	C	-0.0215	0.0037	0.0127	0.0173	-0.5901	0.8102
**rs2331174**	A	-0.0363	0.0037	0.0094	0.0183	-0.2590	0.5049
**rs2448490**	A	-0.0286	0.0038	0.0401	0.0180	-1.4020	0.6563
**rs2518683**	G	-0.0330	0.0052	-0.0059	0.0288	0.1790	0.8741

**rs2700937**	T	0.0216	0.0037	-0.0188	0.0169	-0.8711	0.7969
**rs2736100**	A	-0.0336	0.0036	-0.0226	0.0166	0.6736	0.5001
**rs2810491**	C	0.0399	0.0042	-0.0267	0.0179	-0.6694	0.4543
**rs2836441**	A	-0.0435	0.0052	0.0030	0.0236	-0.0690	0.5425
**rs28550009**	G	0.0737	0.0057	0.0123	0.0251	0.1668	0.3407
**rs2862064**	G	-0.0297	0.0048	0.0109	0.0278	-0.3668	0.9375
**rs2894802**	G	-0.0280	0.0037	0.0063	0.0188	-0.2253	0.6729
**rs290268**	G	0.0220	0.0036	0.0335	0.0168	1.5235	0.8046
**rs2932536**	A	-0.0346	0.0036	-0.0188	0.0164	0.5440	0.4780
**rs2958137**	G	-0.0356	0.0037	-0.0256	0.0168	0.7194	0.4779
**rs3184504**	C	-0.1039	0.0036	-0.1017	0.0225	0.9791	0.2193
**rs34038797**	G	-0.0297	0.0038	-0.0169	0.0176	0.5689	0.5970
**rs342292**	G	-0.0716	0.0037	-0.0200	0.0190	0.2795	0.2659
**rs34623301**	A	0.0663	0.0044	-0.0200	0.0189	-0.3015	0.2856
**rs34667100**	C	-0.0460	0.0036	0.0029	0.0192	-0.0631	0.4178
**rs35427**	G	-0.0256	0.0039	-0.0648	0.0187	2.5355	0.8263
**rs35430985**	A	-0.0254	0.0041	0.0149	0.0195	-0.5858	0.7726
**rs36109901**	C	0.0565	0.0041	0.0010	0.0176	0.0177	0.3113
**rs3731211**	A	0.0411	0.0041	-0.0029	0.0195	-0.0706	0.4749
**rs3741404**	C	-0.0224	0.0038	0.0110	0.0176	-0.4919	0.7914
**rs3747207**	A	-0.0345	0.0045	-0.0214	0.0184	0.6196	0.5387
**rs3778028**	A	0.1172	0.0196	-0.0212	0.0346	-0.1808	0.2967
**rs3804749**	T	-0.0413	0.0037	-0.0016	0.0172	0.0388	0.4169
**rs3809114**	A	-0.0246	0.0037	0.0066	0.0170	-0.2685	0.6927
**rs3809272**	A	-0.0945	0.0040	-0.0468	0.0232	0.4950	0.2463
**rs3819299**	G	0.0936	0.0079	-0.0230	0.0360	-0.2457	0.3851
**rs3844535**	G	0.0237	0.0040	0.0010	0.0191	0.0422	0.8053
**rs3865444**	A	-0.0225	0.0039	-0.0188	0.0195	0.8364	0.8795
**rs409950**	A	0.0646	0.0049	-0.0298	0.0225	-0.4615	0.3502
**rs41315846**	C	0.0575	0.0037	0.0162	0.0174	0.2817	0.3031
**rs4148435**	A	0.0747	0.0066	-0.0299	0.0233	-0.4003	0.3139
**rs4272720**	G	-0.0350	0.0043	0.0149	0.0227	-0.4255	0.6504
**rs429358**	C	-0.0302	0.0051	-0.0036	0.0251	0.1192	0.8314
**rs4388979**	T	-0.0435	0.0037	-0.0158	0.0180	0.3630	0.4147
**rs4411786**	C	-0.0459	0.0041	-0.0174	0.0181	0.3789	0.3956
**rs4432538**	A	-0.0242	0.0037	-0.0089	0.0166	0.3682	0.6889
**rs4470077**	G	0.0293	0.0047	0.0355	0.0236	1.2097	0.8268
**rs4631704**	T	0.0238	0.0038	-0.0091	0.0185	-0.3820	0.7789
**rs4670779**	T	-0.0244	0.0040	-0.0044	0.0198	0.1801	0.8111
**rs4699154**	C	0.0269	0.0040	-0.0119	0.0205	-0.4424	0.7649
**rs4709819**	A	0.0317	0.0037	-0.0132	0.0168	-0.4162	0.5320
**rs4711890**	G	0.0305	0.0041	0.0009	0.0207	0.0295	0.6785
**rs4783186**	C	-0.0422	0.0056	-0.0051	0.0230	0.1209	0.5456

**rs4846217**	T	-0.0361	0.0055	-0.0741	0.0304	2.0503	0.8973
**rs4907622**	C	-0.0304	0.0037	0.0260	0.0175	-0.8544	0.5841
**rs4925750**	C	0.0327	0.0039	-0.0100	0.0176	-0.3057	0.5394
**rs4937127**	G	-0.0320	0.0036	0.0051	0.0187	-0.1595	0.5853
**rs4937333**	C	-0.0322	0.0037	0.0000	0.0165	0.0000	0.5118
**rs4965426**	A	-0.0365	0.0052	-0.0287	0.0221	0.7859	0.6155
**rs56036086**	A	-0.0511	0.0053	-0.0249	0.0305	0.4872	0.5989
**rs56043070**	A	-0.1388	0.0070	-0.0229	0.0383	0.1650	0.2761
**rs56125409**	G	-0.0506	0.0056	0.0347	0.0222	-0.6855	0.4451
**rs57274573**	T	0.0280	0.0049	0.0583	0.0250	2.0806	0.9624
**rs58530613**	C	0.0346	0.0058	0.0262	0.0310	0.7572	0.9050
**rs59739601**	G	-0.0551	0.0069	0.0011	0.0235	-0.0200	0.4265
**rs59865663**	A	0.0408	0.0046	0.0091	0.0214	0.2230	0.5250
**rs6060983**	C	-0.0334	0.0040	-0.0316	0.0229	0.9474	0.6959
**rs619460**	A	-0.0237	0.0037	-0.0312	0.0182	1.3149	0.7947
**rs6425521**	A	-0.0652	0.0046	-0.0296	0.0202	0.4537	0.3113
**rs6445967**	C	-0.0305	0.0039	-0.0074	0.0178	0.2429	0.5851
**rs655029**	A	0.0735	0.0041	-0.0068	0.0230	-0.0925	0.3130
**rs655641**	G	0.0266	0.0045	0.0133	0.0197	0.4991	0.7441
**rs6556471**	C	-0.0462	0.0039	0.0102	0.0177	-0.2209	0.3838
**rs670179**	A	0.0282	0.0037	0.0225	0.0190	0.7975	0.6816
**rs6756513**	A	-0.0251	0.0041	-0.0056	0.0178	0.2229	0.7093
**rs6925716**	C	0.0300	0.0037	-0.0053	0.0166	-0.1764	0.5528
**rs6961069**	T	0.0261	0.0038	0.0280	0.0172	1.0742	0.6778
**rs6993770**	T	-0.0700	0.0040	0.0005	0.0177	-0.0071	0.2529
**rs7146395**	C	-0.0227	0.0039	0.0016	0.0180	-0.0704	0.7922
**rs7178196**	A	-0.0345	0.0049	-0.0140	0.0197	0.4060	0.5742
**rs7249921**	T	-0.0237	0.0037	0.0266	0.0196	-1.1223	0.8458
**rs73000965**	A	-0.0281	0.0039	-0.0118	0.0186	0.4193	0.6636
**rs73109811**	T	0.0407	0.0047	0.0264	0.0300	0.6482	0.7404
**rs75080135**	C	0.1019	0.0048	0.0050	0.0221	0.0491	0.2169
**rs75501914**	A	0.0494	0.0074	0.0021	0.0302	0.0425	0.6114
**rs7641175**	A	0.0458	0.0044	0.0124	0.0249	0.2707	0.5443
**rs7665147**	A	-0.0317	0.0047	0.0440	0.0188	-1.3867	0.6273
**rs7696658**	T	0.0215	0.0037	0.0059	0.0173	0.2748	0.8071
**rs77300440**	T	0.0686	0.0067	-0.0654	0.0306	-0.9535	0.4557
**rs77320796**	G	0.0268	0.0042	0.0722	0.0252	2.6943	1.0298
**rs7776054**	G	0.1192	0.0042	0.0082	0.0188	0.0688	0.1578
**rs7788849**	A	-0.0443	0.0062	-0.0233	0.0311	0.5262	0.7061
**rs7811142**	T	0.0358	0.0046	0.0109	0.0263	0.3048	0.7366
**rs78265569**	A	-0.0458	0.0065	0.0158	0.0416	-0.3451	0.9099
**rs7833924**	G	0.0439	0.0037	-0.0108	0.0185	-0.2460	0.4219
**rs78565404**	T	0.1420	0.0085	-0.0166	0.0479	-0.1169	0.3373

**rs78909033**	A	0.0653	0.0053	-0.0062	0.0367	-0.0949	0.5620
**rs7950696**	C	0.0274	0.0037	-0.0354	0.0175	-1.2912	0.6611
**rs8037137**	C	-0.0354	0.0054	-0.0395	0.0206	1.1143	0.6059
**rs8073060**	A	0.0300	0.0040	-0.0211	0.0199	-0.7045	0.6711
**rs8137128**	T	-0.0418	0.0037	-0.0092	0.0174	0.2199	0.4164
**rs8176747**	G	-0.0546	0.0075	-0.0323	0.0257	0.5916	0.4777
**rs853195**	A	-0.0240	0.0038	-0.0139	0.0202	0.5797	0.8474
**rs926326**	G	-0.0475	0.0043	-0.0070	0.0247	0.1474	0.5204
**rs928391**	C	-0.0260	0.0041	-0.0179	0.0210	0.6877	0.8140
**rs9402633**	T	0.0328	0.0045	0.0665	0.0200	2.0287	0.6699
**rs9524862**	A	0.0351	0.0037	-0.0080	0.0167	-0.2280	0.4765
**rs9809116**	G	0.0265	0.0037	-0.0026	0.0172	-0.0981	0.6492
**rs9810259**	G	-0.0409	0.0037	-0.0138	0.0171	0.3372	0.4189
**rs9908158**	C	0.0312	0.0038	-0.0143	0.0167	-0.4587	0.5386
**rs9974653**	C	-0.0295	0.0039	0.0282	0.0197	-0.9549	0.6792

EA: effect allele; GX: SNP-platelet count association; GY: SNP-small vessel stroke; MR: Mendelian randomization; SE: standard error; SNP: single nucleotide polymorphism.

Supplementary Table IX: SNPs excluded from the outlier-corrected MR-PRESSO analysis (Verbanck et al, 2018)	
Coronary artery disease	
SNP	p-value
rs11556924	<0.0207
rs2068888	<0.0207
rs216191	<0.0207
rs2255531	<0.0207
rs3184504	<0.0207
rs429358	<0.0207
rs7665147	<0.0207
rs9462031	0.0414
Myocardial infarction	
rs11556924	<0.0206
rs2068888	0.0412
rs216191	0.0206
rs2255531	<0.0206
rs3184504	<0.0206
rs429358	<0.0206
rs7665147	0.0412
Ischemic stroke	
rs174548	<0.0203
rs2068888	<0.0203
rs3184504	<0.0203
rs35427	<0.0203
Large-artery atherosclerotic stroke	
rs3184504	<0.0206
Small vessel stroke	
rs3184504	<0.0183

MR-PRESSO: mendelian randomisation-pleiotripy residual sum and outlier; SNP: single nucleotide polymorphism.

Supplemental Table X: Heterogeneity statistics in the examined associations between platelet count and the related outcomes

Outcome	I^2_{MR} (%)	95% CI (%)	Cochran Q	p [Q]	Rucker Q'	p [Q']	Q-Q'	p [Q-Q']
Coronary artery stroke	53.7	45.7-60.4	444.6705	**1.31E-19**	436.5756	**7.96E-19**	8.0949	**0.004439**
Myocardial infarction	47.8	38.5-55.6	392.4631	**6.72E-14**	387.7053	**1.53E-13**	4.7578	**0.029166**
Ischemic stroke	39.2	27.9-48.8	332.396	**2.02E-08**	329.2274	**2.96E-08**	3.1686	0.075067
Large artery atherosclerotic stroke	19.3	3.3-32.8	282.9321	**0.000252**	282.0936	**0.000239**	0.8385	0.359826
Cardioembolic stroke	27.5	13.5-39.3	252.9402	**0.011161**	251.5832	**0.011473**	1.357	0.244058
Small vessel stroke	22.2	5.8-35.7	233.9199	**0.005658**	233.7813	**0.004983**	0.1386	0.709677

Bold indicates statistical significance ($p<0.05$).

Supplementary Table XI: Key findings from observational studies investigating platelet count and risk of CVD					
Study	Population	Exposure	CVD subtype investigated	Mean follow-up (years)	Result
Ischemic stroke					
Chen et al, 2015[2]<br>Case-control	N=122 cases (53 large-artery atherosclerotic and 59 small artery occlusion)<br>N=35 controls<br>Country: China<br>Age (mean±SD):<br>Cases – 65.7±1.2 years<br>LAA – 67.7±1.4 years<br>SAO – 68.0±1.3 years<br>(p-value>0.05)	Platelet count measured on admission with stroke, admitted within 48 hours of symptom onset	Acute ischemic stroke	-	No significant difference in platelet count between cases and controls, P>0.05, raw data not reported
Du et al, 2016[3]<br>Case-control	N=281 cases<br>N=200 controls<br>Country: China<br>Age (mean±SD):<br>Cases – 66.3±11.0 years<br>Controls – 65.8±13.0 years<br>(p-value>0.05)	Platelet count measured within 2 hours of admission with stroke, average of 6 hours between symptom onset and admission	First ischemic stroke	-	OR (95% CI) of ischemic stroke for stated platelet count compared with platelet count of 100-300x10^9/L:<br><100x10^9/L = 0.76 (0.43-1.36) (p=0.36)<br>>300x10^9/L = 4.49 (1.49-13.53) (p=0.008)
Mayda-Domaç et al, 2010[4]<br>Case-control	N=384 cases<br>N=208 controls<br>Country: Turkey<br>Age (mean±SD):<br>Cases – 65.6±12.6 years<br>Controls – 63.2±13.7 years<br>(p-value=0.061)	Platelet count measured within 24 hours of admission with stroke, admitted with 24 hours of symptom onset	Ischemic stroke	-	Platelet count significantly higher in cases versus controls (281±93 versus 269±70x10^9/L, (281±93 versus 269±70x10^3/mL) P<0.001)
Szikszai et al, 2003[5]<br>Case-control	N=33 cases<br>N=33 controls<br>Country: Hungary<br>Age (mean±SD):<br>Cases: 64±11 years<br>Controls: 62±10 years	Platelet count measured during admission (after overnight fast and within 72 hours of symptom onset)	Ischemic stroke	-	Platelet count in cases versus controls: 275±88x10^9/L (g/L) versus 200±63x10^9/L (g/L), p-value<0.01
CAD					

Meade et al, 1997[6]<br>Cohort	N=1369 total cohort<br>N=181 cases<br>Country: UK, Caucasians (Northwick Park Heart Study)<br>Age: 40-64 years (raw data not available)	Platelet count	CAD	16.1	No significant association between platelet count and risk of CAD (P>0.05), raw data not reported
Molnar et al, 2011[7]<br>Cohort	N=40,787 total cohort<br>N=11,051 cardiovascular deaths<br>Country: USA (hemodialysis patients)<br>Age (mean±SD): 61±15years	Platelet count	CAD<br>Myocardial infarction	2.9	Number of patients with CAD for the stated range of platelet count:<br>$<150x10^9/L$ ($<150x10^3/\mu L$): 19<br>$150\text{-}199x10^9/L$: 18<br>$200\text{-}249x10^9/L$: 17<br>$250\text{-}299x10^9/L$: 17<br>$300\text{-}349x10^9/L$: 17<br>$350\text{-}399x10^9/L$: 16<br>$\geq 400x10^9/L$: 17<br>(p-value=0.012)<br><br>Number of patients with myocardial infarction for the stated range of platelet count:<br>$<150x10^9/L$: 6<br>$150\text{-}199x10^9/L$: 5<br>$200\text{-}249x10^9/L$: 5<br>$250\text{-}299x10^9/L$: 5<br>$300\text{-}349x10^9/L$: 5<br>$350\text{-}399x10^9/L$: 5<br>$\geq 400x10^9/L$: 5<br>(p-value=0.22)
Panwar et al, 2011[8]<br>Case-control	N=165 cases<br>N=199 controls<br>Country: India<br>Age (mean±SD):<br>Cases – 43.3±5.6 years<br>Controls – 43.1±5.9 years<br>(p-value>0.05)	Platelet count measured within 24 hours of admission for acute coronary event, unspecified time since symptom onset	Myocardial infarction<br>Unstable angina	-	Platelet count significantly higher in cases versus controls (P<0.05), raw data not reported
Yaghoubi et al, 2013[9]<br>Retrospective case-control study	N=210 myocardial infarction<br>N=211 unstable angina<br>N=210 controls<br>Country: Iran<br>Age (mean±SD):	Platelet count measured during admission with chest pain, admitted within	Myocardial infarction<br>Unstable angina	-	Platelet count significantly lower in patients versus controls (p-value<0.001):<br>Myocardial infarction: $207.9\pm58.4x10^9/L$<br>Unstable angina: $220.2\pm65.8x10^9/L$<br>Controls: $238.0\pm56.1x10^9/L$

	Myocardial infarction: 62.7±14.2 years<br>Unstable angina: 63.5±13.1 years<br>Controls: 60.6±12.8 years (no test for difference between groups)	24 hours of symptom onset			
Khode et al, 2012[10]<br>Case-control	N=63 CAD (39 acute myocardial infarction, 24 stable CAD)<br>N=65 controls<br>Country: India<br>Age (mean±SD):<br>Cases: 55.1±9.8 years<br>Controls: 54.5±8.7 years (p-value=0.677)	Platelet count (measured within 4 hours of symptom onset)	CAD<br>Acute myocardial infarction<br>Stable CAD	-	Platelet count in CAD cases versus controls: 288.0±108.6 versus 282.4±87.8x10^{9}/L (p-value=0.747)<br>Platelet count in acute myocardial infarction (291.1±104.7x10^{9}/L) versus stable CAD (283.1±116.7x10^{9}/L) and controls (282.4±87.8x10^{9}/L) (p-value=0.904)
All CVD					
van der Bom et al, 2009[11]<br>Cohort	N=5766 total cohort<br>N= 821 myocardial infarction<br>N=807 ischemic strokes<br>Country: USA (Cardiovascular Health Study)<br>Age (mean): 73 years (SD not reported)	Platelet count	Myocardial infarction<br>Ischemic stroke	12-15*	Hazard ratio (95% CI) of myocardial infarction for stated platelet count relative to 200-299 x10^{9}/L:<br>48-99x10^{9}/L: 0.40 (0.10-1.61)<br>100-199x10^{9}/L: 0.95 (0.80-1.12)<br>300-399x10^{9}/L: 0.92 (0.75-1.14)<br>400-1223x10^{9}/L: 0.55 (0.31-0.98)<br><br>Hazard ratio (95% CI) of ischemic stroke for stated platelet count relative to 200-299 x10^{9}/L:<br>48-99x10^{9}/L: 0.97 (0.36-2.61)<br>100-199x10^{9}/L: 1.13 (0.95-1.34)<br>300-399x10^{9}/L: 0.98 (0.80-1.20)<br>400-1223x10^{9}/L: 1.10 (0.72-1.69)
Vinholt et al, 2016[12]<br>Cohort	N=21,252 total cohort<br>N= 618 cases<br>Country: Denmark (Danish General Suburban Population Study)<br>Age (mean±SD): 53±13.6 years	Platelet count	CVD (including myocardial infarction, ischemic stroke and peripheral vascular disease)	3.5	Hazard ratio (95% CI) of CVD for stated platelet count relative to 201-250x10^{9}/L for all subjects:<br>100-200 x10^{9}/L: 1.10 (0.88-1.39)<br>251-300 x10^{9}/L: 1.20 (0.98-1.49)<br>301-450 x10^{9}/L: 1.32 (1.03-1.69)
Lassale et al, 2018[13]<br>Cohort	N=14,362 total cohort<br>N=922 CVD (196 stroke, 589 CHD)	Platelet count	CVD (including CAD, cardiac arrest, heart failure, stroke,	11.4	HR (95% CI) of CVD in stated platelet count range compared with 150-400x10^{9}/L:<br><150x10^{9}/L: 1.01 (0.66-1.52)

	Country: Netherlands (EPIC-NL)<br>Age (mean±SD):<br>All: 47.8±11.7 years<br>CVD: 54.7±8.8 years<br>Stroke: 56.1±9.8 years<br>CHD: 54.3±8.2 years<br>Significant difference between cases and non-cases, p-value<0.05)		pulmonary embolism and infarction, peripheral vascular disease, atherosclerosis, aortic aneurysm and dissection, other aneurysms, arterial embolism and arterial thrombosis)<br>Stroke<br>CAD		≥400x10^9/L: 1.49 (1.00-2.22)<br><br>HR (95% CI) of stroke in stated platelet count range compared with 150-400x10^9/L:<br><150x10^9/L: 1.91 (0.93-3.93)<br>≥400x10^9/L: 1.16 (0.43-3.15)<br><br>HR (95% CI) of CAD in stated platelet count range compared with 150-400x10^9/L:<br><150x10^9/L: 0.74 (0.39-1.39)<br>≥400x10^9/L: 1.69 (1.03-2.80)
Zakai et al, 2007[14]<br>Cohort	N=4510 total cohort<br>N=1700 cases<br>Country: USA (Cardiovascular Health Study)<br>Age (median±SD): 68±3.8 years	Platelet count	CVD events (myocardial infarction, angina, angioplasty, coronary artery bypass grafting, stroke, transient ischemic attack)	9.2	HR (95% CI) of cardiovascular events per SD increase in platelet count: 1.02 (0.97-1.08)

CAD: coronary artery disease; CI: confidence interval; CVD cardiovascular disease; EPIC-NL: European Prospective Investigation into Cancer and Nutrition-Netherlands; HR: hazard ratio; OR: odds ratio; SD: standard deviation
*Range of duration of follow-up, mean not reported

Supplemental Figures

Supplementary Figure I: Funnel plot (A) and radial plot (B) for the association between genetically determined platelet count and risk of coronary artery disease.

A) B)

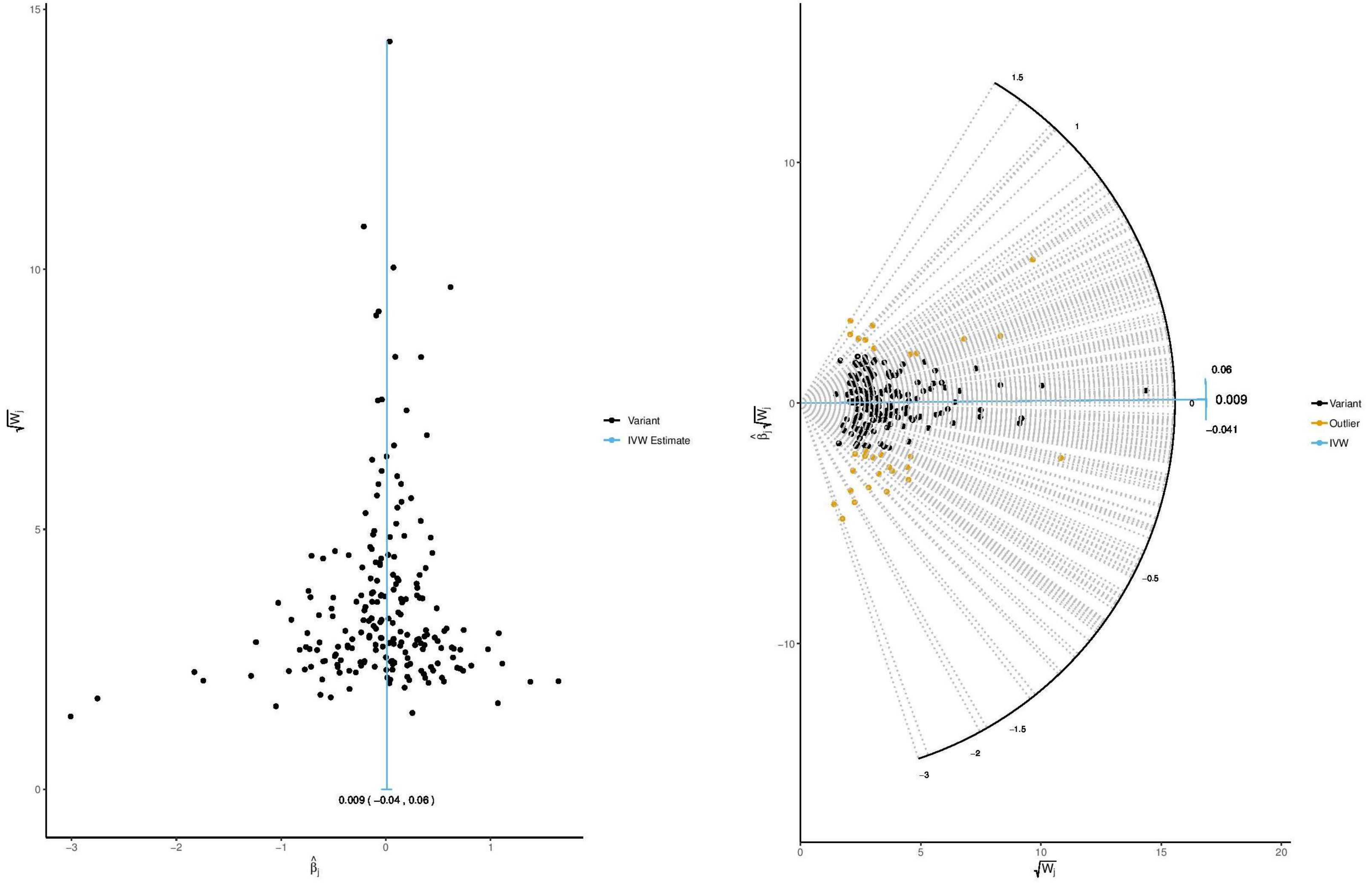

Supplementary Figure II: Funnel plot (A) and radial plot (B) for the association between genetically determined platelet count and risk of myocardial infarction.

A) B)

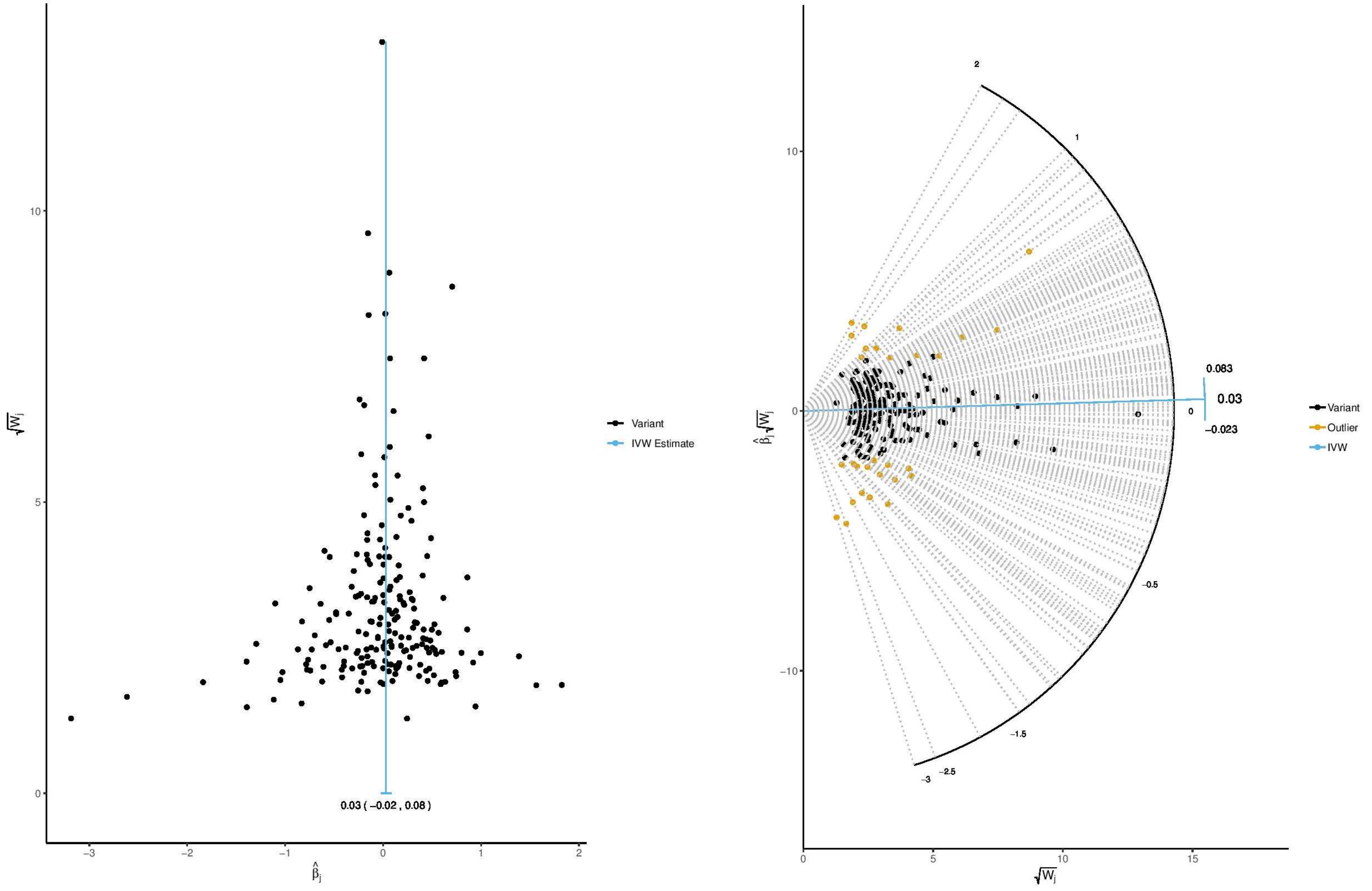

Supplementary Figure III: Funnel plot (A) and radial plot (B) for the association between genetically determined platelet count and risk of ischemic stroke.

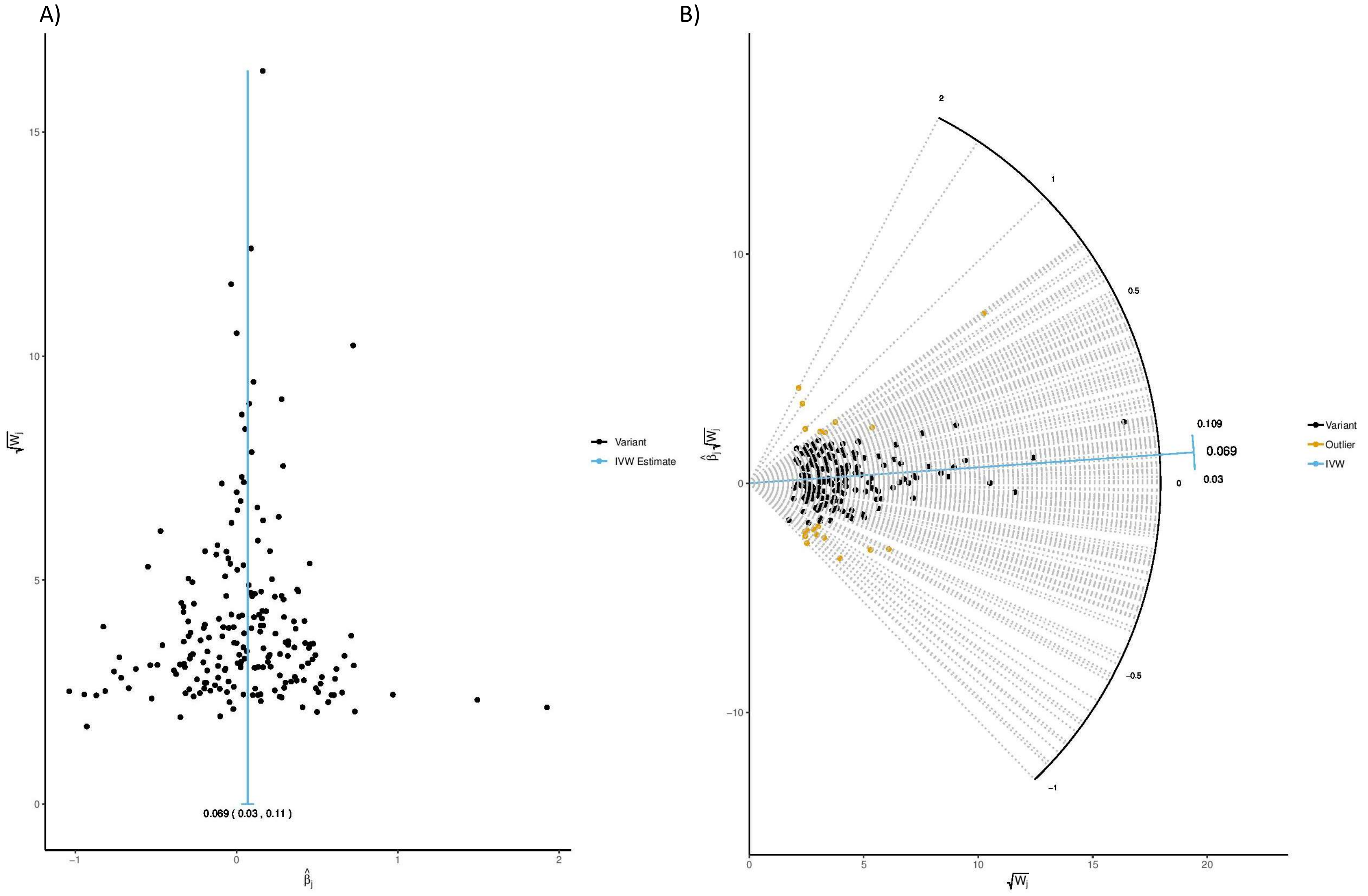

Supplementary Figure IV: Funnel plot (A) and radial plot (B) for the association between genetically determined platelet count and risk of large-artery atherosclerotic stroke.

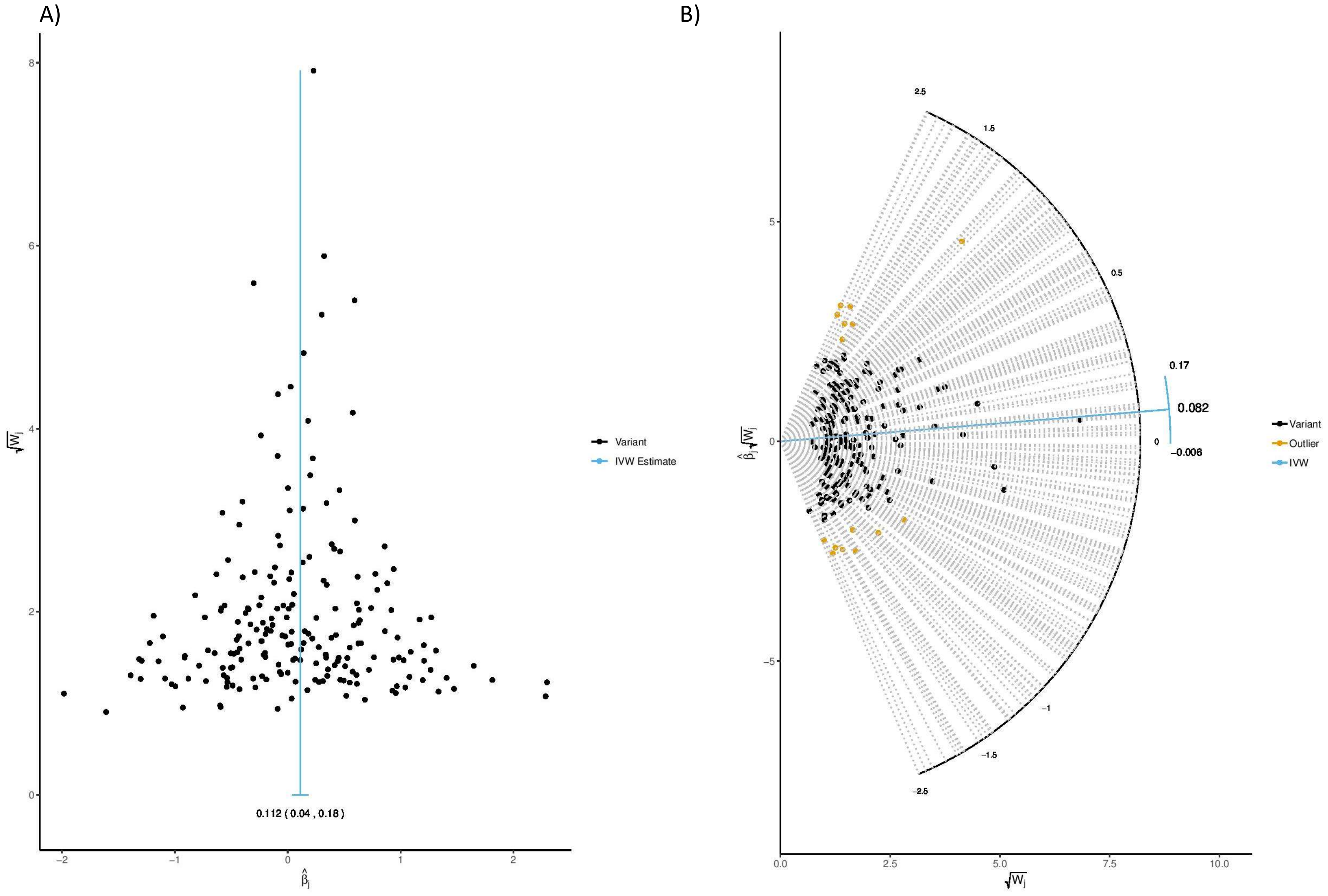

Supplementary Figure V: Funnel plot (A) and radial plot (B) for the association between genetically determined platelet count and risk of cardioembolic stroke.

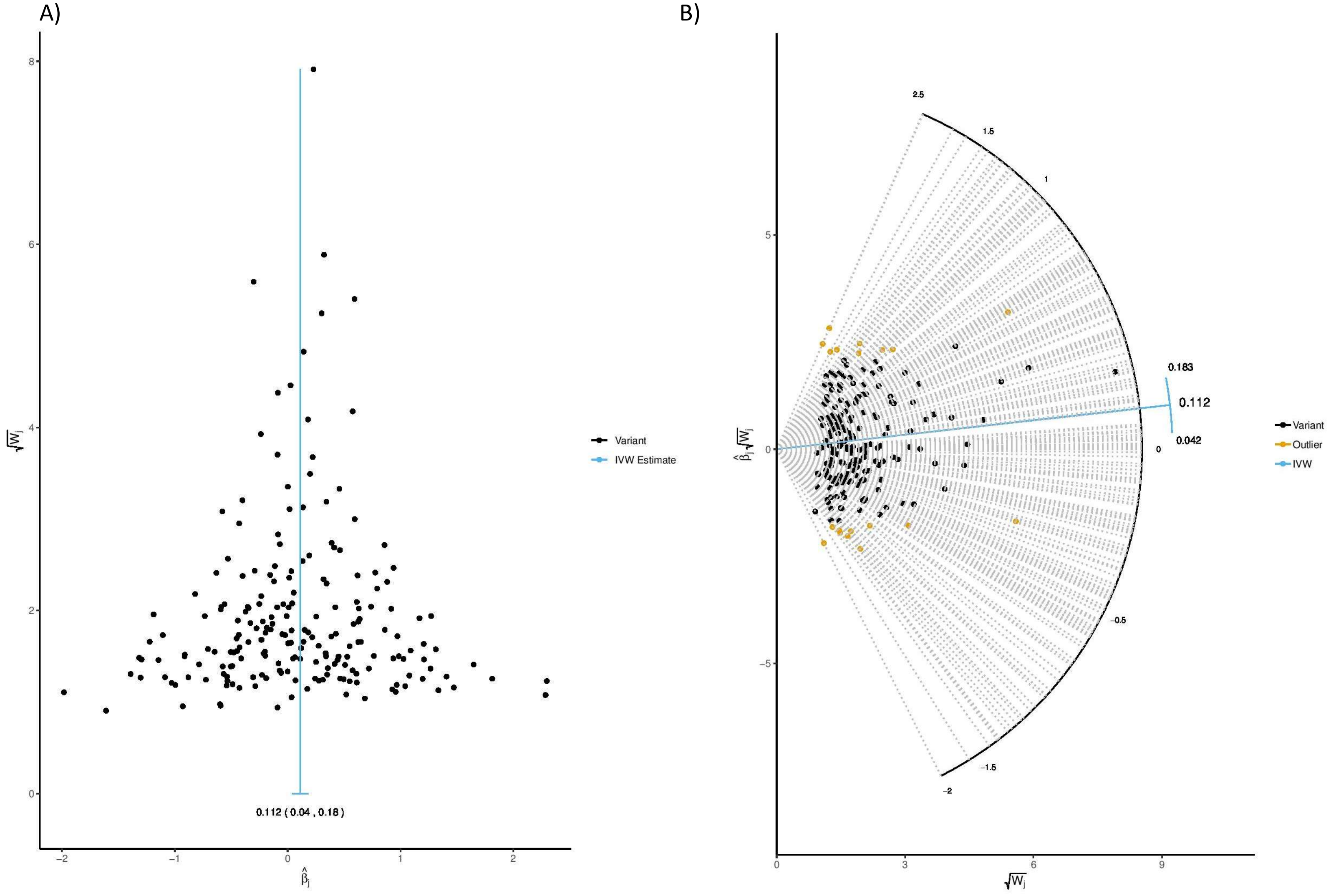

Supplementary Figure VI: Funnel plot (A) and radial plot (B) for the association between genetically determined platelet count and risk of small vessel stroke.

A)

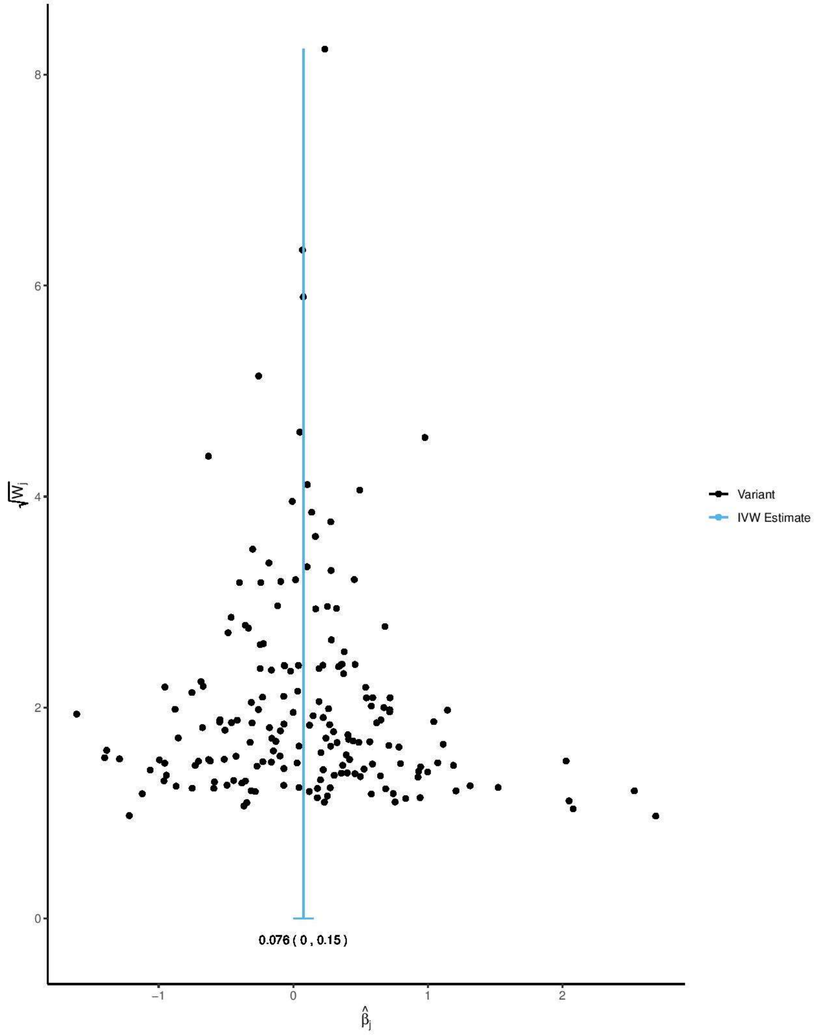

B)

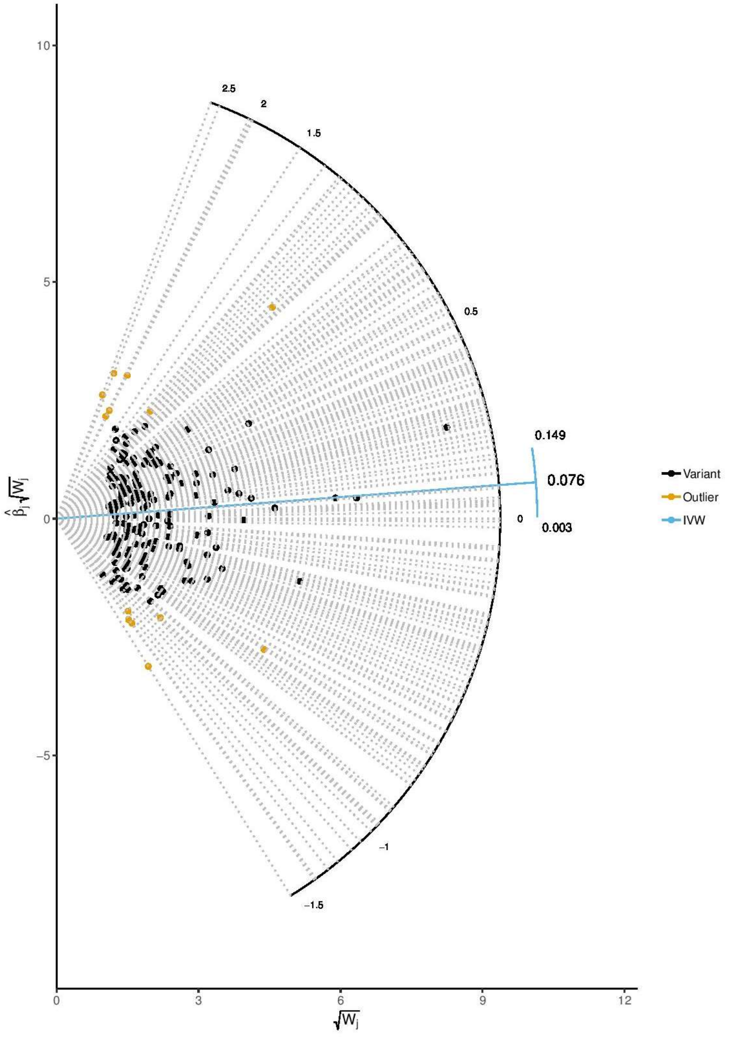

Supplemental References

1. Malik R, Chauhan G, Traylor M, Sargurupremraj M, Okada Y, Mishra A, et al. Multiancestry genome-wide association study of 520,000 subjects identifies 32 loci associated with stroke and stroke subtypes. *Nat Genet*. 2018;50:524-537
2. Chen Y, Xiao Y, Lin Z, Xiao X, He C, Bihl JC, et al. The role of circulating platelets microparticles and platelet parameters in acute ischemic stroke patients. *J Stroke Cerebrovasc Dis*. 2015;24:2313-2320
3. Du J, Wang Q, He B, Liu P, Chen JY, Quan H, et al. Association of mean platelet volume and platelet count with the development and prognosis of ischemic and hemorrhagic stroke. *Int J Lab Hematol*. 2016;38:233-239
4. Mayda-Domaç F, Misirli H, Yilmaz M. Prognostic role of mean platelet volume and platelet count in ischemic and hemorrhagic stroke. *J Stroke Cerebrovasc Dis*. 2010;19:66-72
5. Szikszai Z, Fekete I, Imre SG. A comparative study of hemorheological parameters in transient ischemic attack and acute ischemic stroke patients: Possible predictive value. *Clin Hemorheol Microcirc*. 2003;28:51-57
6. Meade TW, Cooper JA, Miller GJ. Platelet counts and aggregation measures in the incidence of ischaemic heart disease (ihd). *Thromb Haemost*. 1997;78:926-929
7. Molnar MZ, Streja E, Kovesdy CP, Budoff MJ, Nissenson AR, Krishnan M, et al. High platelet count as a link between renal cachexia and cardiovascular mortality in end-stage renal disease patients. *Am J Clin Nutr*. 2011;94:945-954
8. Panwar RB, Gupta R, Gupta BK, Raja S, Vaishnav J, Khatri M, et al. Atherothrombotic risk factors & premature coronary heart disease in india: A case-control study. *Indian J Med Res*. 2011;134:26-32
9. Yaghoubi A, Golmohamadi Z, Alizadehasl A, Azarfarin R. Role of platelet parameters and haematological indices in myocardial infarction and unstable angina. *J Pak Med Assoc*. 2013;63:1133-1137
10. Khode V, Sindhur J, Kanbur D, Ruikar K, Nallulwar S. Mean platelet volume and other platelet volume indices in patients with stable coronary artery disease and acute myocardial infarction: A case control study. *J Cardiovasc Dis Res*. 2012;3:272-275
11. van der Bom JG, Heckbert SR, Lumley T, Holmes CE, Cushman M, Folsom AR, et al. Platelet count and the risk for thrombosis and death in the elderly. *J Thromb Haemost*. 2009;7:399-405
12. Vinholt PJ, Hvas AM, Frederiksen H, Bathum L, Jørgensen MK, Nybo M. Platelet count is associated with cardiovascular disease, cancer and mortality: A population-based cohort study. *Thromb Res*. 2016;148:136-142
13. Lassale C, Curtis A, Abete I, van der Schouw YT, Verschuren WMM, Lu Y, et al. Elements of the complete blood count associated with cardiovascular disease incidence: Findings from the epic-nl cohort study. *Sci Rep*. 2018;8:3290
14. Zakai NA, Katz R, Jenny NS, Psaty BM, Reiner AP, Schwartz SM, et al. Inflammation and hemostasis biomarkers and cardiovascular risk in the elderly: The cardiovascular health study. *J. Thromb. Haemost*. 2007;5:1128-1135
