# Supplementary figures and images for: Genetically Determined Platelet Count and Risk of Cardiovascular Disease: Mendelian Randomization Study
Source: Arterioscler Thromb Vasc Biol. 2018 Oct 11;38(12):2862–9. doi: 10.1161/ATVBAHA.118.311804 (PMC6250250; doi:10.1161/ATVBAHA.118.311804)

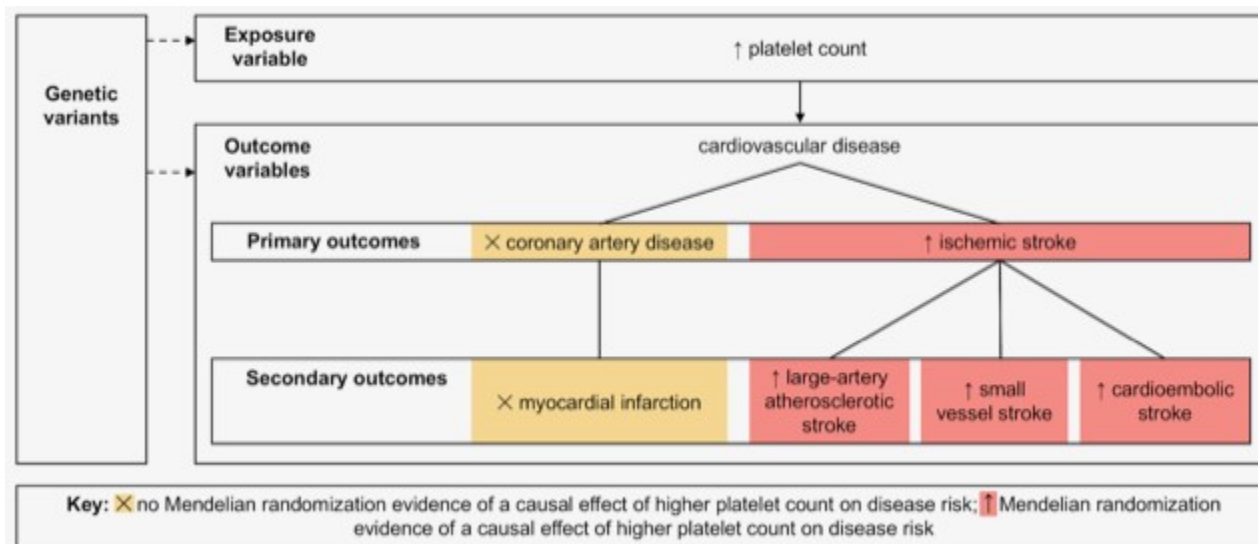

Supplement: Supplementary file 2 [file atv-38-2862-s002.pdf]
